# Use of the Smartphone App WhatsApp as an E-Learning Method for Medical Residents: Multicenter Controlled Randomized Trial

**DOI:** 10.2196/12825

**Published:** 2019-04-09

**Authors:** Thomas Clavier, Julie Ramen, Bertrand Dureuil, Benoit Veber, Jean-Luc Hanouz, Hervé Dupont, Gilles Lebuffe, Emmanuel Besnier, Vincent Compere

**Affiliations:** 1 Department of Anesthesiology and Critical Care Rouen University Hospital Rouen France; 2 Department of Anesthesiology and Critical Care Caen University Hospital Normandie Université Caen France; 3 Department of Anesthesiology and Critical Care Amiens University Hospital Amiens France; 4 Pôle d’Anesthésie-Réanimation Lille University Hospital Lille France

**Keywords:** education, medical, graduate/methods, educational measurement, anesthesiology, internship and residency, trauma, hemorrhage, mobile applications, WhatsApp, smartphone, teaching materials, mobile phone

## Abstract

**Background:**

The WhatsApp smartphone app is the most widely used instant messaging app in the world. Recent studies reported the use of WhatsApp for educational purposes, but there is no prospective study comparing WhatsApp’s pedagogical effectiveness to that of any other teaching modality.

**Objective:**

The main objective of this study was to measure the impact of a learning program via WhatsApp on clinical reasoning in medical residents.

**Methods:**

This prospective, randomized, multicenter study was conducted among first- and second-year anesthesiology residents (offline recruitment) from four university hospitals in France. Residents were randomized in two groups of online teaching (WhatsApp and control). The WhatsApp group benefited from daily delivery of teaching documents on the WhatsApp app and a weekly clinical case supervised by a senior physician. In the control group, residents had access to the same documents via a traditional computer electronic learning (e-learning) platform. Medical reasoning was self-assessed online by a script concordance test (SCT; primary parameter), and medical knowledge was assessed using multiple-choice questions (MCQs). The residents also completed an online satisfaction questionnaire.

**Results:**

In this study, 62 residents were randomized (32 to the WhatsApp group and 30 to the control group) and 22 residents in each group answered the online final evaluation. We found a difference between the WhatsApp and control groups for SCTs (60% [SD 9%] vs 68% [SD 11%]; *P*=.006) but no difference for MCQs (18/30 [SD 4] vs 16/30 [SD 4]; *P*=.22). Concerning satisfaction, there was a better global satisfaction rate in the WhatsApp group than in the control group (8/10 [interquartile range 8-9] vs 8/10 [interquartile range 8-8]; *P*=.049).

**Conclusions:**

Compared to traditional e-learning, the use of WhatsApp for teaching residents was associated with worse clinical reasoning despite better global appreciation. The use of WhatsApp probably contributes to the dispersion of attention linked to the use of the smartphone. The impact of smartphones on clinical reasoning should be studied further.

## Introduction

Many computer-based teaching materials have been developed in recent years, and electronic learning (e-learning) is becoming increasingly popular in medical schools, with the appearance of guides on e-learning deployment [1]. E-learning has many organizational advantages over face-to-face teaching: temporal and spatial flexibility for learners, live updates, and easy and uniform dissemination of teaching resources for teachers. Moreover, the emergence of social networks facilitates personal and professional communication and exchange [2,3]. The time spent on mobile phone screens per day (“screen time”) has increased exponentially since the introduction of the latest-generation phones known as smartphones, in particular, among young people, leading to a growing interest of mobile learning (m-learning) among teachers [1,4-6].

The WhatsApp smartphone app, developed by WhatsApp Inc (owned by Facebook Inc, Menlo Park, CA), is the most widely used instant messaging app in the world, with more than one billion active users per month and more than 40 billion WhatsApp messages exchanged each day in 2016 [7]. It allows communication between group participants without the need for unity in place or time. Participants are free to choose when they want to access the information posted and can view and interact with other group members regarding the information delivered at any time. In view of its popularity with medical students, it seems interesting to envisage a new use of WhatsApp, by orienting it toward an educational objective (with the opportunity to recover screen time from students) [8,9]. The first reports of the use of this app for educational purposes date to the early 2017, for teaching medical students or training pathology residents [10,11]. Both of these observational studies showed satisfaction among WhatsApp participants and highlighted the ease of use and the quick access to lessons through the app. However, there is no prospective study comparing WhatsApp to any other teaching modality.

Residents involved in tutored practice exchange groups have better medical reasoning, as evaluated by the script concordance test (SCT), which is a well-validated medical reasoning–assessment tool for residents [12-14]. Similar to practice exchange groups, WhatsApp allows direct communication between teachers and students with the possibility of discussing real clinical cases and commenting on residents’ management of the case. Thus, we hypothesized that WhatsApp could have the same effect as practice exchange groups on clinical reasoning. The main objective of this study was to measure the impact of WhatsApp on clinical reasoning by using the SCT. As severe trauma is one of the leading causes of death in the world, with more than 5 million deaths, and posttraumatic hemorrhage is the leading cause of mortality, we selected posttraumatic hemorrhage management as the topic for our teaching and evaluation [15].

## Methods

### Population Selection

This prospective, randomized, unblinded, multicenter study was conducted among first- and second-year anesthesiology residents from four French university hospitals with trauma center (Amiens, Caen, Lille, and Rouen) comparing WhatsApp to a control group. Since computer-based e-learning did not show any noninferiority compared to traditional teaching, it was chosen as a control teaching platform for this study [16,17]. The Ethics and Evaluation Committee for Non-Interventional Research of Rouen University Hospital approved the study (E2017-37). All participants received information before any study procedures were undertaken, and residents were invited to participate as subjects in the study. All information about and during the trial was sent by email, and participants knew that the WhatsApp group was the “intervention” group. Agreement to participate was provided online by email or telephone by each resident who could stop participating at any time.

The inclusion criteria were ongoing medical residency, possession of a mobile phone that could download the WhatsApp app, and attestation for agreement to use WhatsApp for this study. Noninclusion criteria were refusal to participate, noncompatibility of a mobile phone to download WhatsApp, and failure to download WhatsApp. This study was carried out in addition to the official teaching program of the residents and was not integrated into usual teaching nor did it replace previous teaching.

The primary measure was medical reasoning evaluated by the SCT. The secondary parameters were medical knowledge measured by multiple-choice questions (MCQ); feasibility and acceptability of using WhatsApp, assessed by collecting resident testimonials; the Cronbach coefficient alpha, calculated after optimizing the test for SCT [18]; and self-assessment (by quantitative and semiquantitative numerical scales using a satisfaction questionnaire) of time spent working, quality of teaching, global satisfaction, teachers’ availability, impression that the teaching met the learning objectives, relevance of clinical cases, and volume of teaching documents used.

### Study Procedures

After inclusion, residents were randomized with their last names in two groups (WhatsApp and control) by author TC, using an online open-access app for stratification according to the student’s hospital and year of residency [19]. Concerning intervention or evaluation, this was a purely app- or Web-based trial without face-to-face components between resident and teachers. After randomization and before the beginning of the course, all residents were emailed a short evaluation with 10 SCTs and 10 MCQs on basic knowledge of anesthesiology (intensive care, regional anesthesia, obstetrics, etc) to check the initial comparability of the groups and to familiarize first-year residents with the SCT. Students returned the SCTs and MCQs by email after completing them. After this short evaluation, the WhatsApp group benefited from daily delivery of teaching documents specially prepared for easy readability on a smartphone (from Monday to Thursday, morning and afternoon; 2-4 documents/day; Multimedia Appendix 1) through the WhatsApp app. These documents were inspired by the most recent guidelines on the management of traumatic hemorrhagic shock and were validated by anesthesiology teachers (VC, BV, and BD) [20,21]. It is strongly suggested that resolution of clinical cases have a significant role in the acquisition of medical reasoning [22,23]. Thus, every Friday, residents were given a “step-by-step” clinical case on WhatsApp for 3-4 hours (Multimedia Appendix 2), supervised by a senior anesthesiologist (TC) who questioned the residents (to create an interest in the clinical cases) and provided them with feedback and validation or correction, if necessary, as described in the practice exchange groups [12]. Several screenshots of the use of WhatsApp for learning purposes during the protocol are presented in the Multimedia Appendix 3. The total duration of teaching was 3 weeks, and the choice of the length of the teaching period was based on both the availability of teachers and the estimated acceptability of students. In the control group, residents had access to the same documents via a computer e-learning platform, and the senior anesthesiologist teacher was available by email. They had access to the three clinical cases with their answers but had no live interaction with a teacher. The two groups had the same program and learning objectives. Participants did not receive any documents during the weekends and were free to stop the courses at any time. The characteristics of WhatsApp-assisted m-learning and traditional e-learning used in this study are summarized in Table 1. At the end of the teaching period, the two groups had the same formative evaluation by 29 SCTs and 30 MCQs sent by email and completed during the month following the end of the teaching period (Multimedia Appendices 4 and 5). Students returned the SCTs and MCQs by email after completing them. In case of nonresponse, residents were sent two reminders by email before being considered lost to follow-up. The residents of the two groups who responded to the final evaluation completed an online satisfaction questionnaire specifically created for this study (not previously validated in the literature; Multimedia Appendix 6).

**Table 1:** Characteristics of WhatsApp-assisted m-learning and control e-learning used in this study. The two groups had the same program, learning objectives, and educational documents.

Characteristics	WhatsApp group (m-learning^a^)	Control group (traditional e-learning^b^)
Length of teaching	3 weeks	3 weeks
Accessibility of educational documents	Sent daily on WhatsApp from Monday to Friday	Available on a computer e-learning platform
Teacher availability	Available and can be contacted by WhatsApp	Available and can be contacted by email
Conduct of clinical cases	Live on Friday on WhatsApp, with questions and answers from the teacher as the case progresses	Cases accessible on the platform with their answers. Teacher available if the student has any questions.

^a^m-learning: mobile learning.

^b^e-learning: electronic learning.

### Design of the Script Concordance Test and Multiple-Choice Questions

The MCQs and SCTs were written by one of the teachers (TC). They were directly related to issues covered during teaching and were reviewed (and possibly modified, if needed) by two other teachers (JR and VC). The SCTs were designed as previously described [12,24]. The SCT confronted the residents with authentic uncertain clinical situations concerning traumatic hemorrhagic shock, which were described in vignettes, each corresponding to one of the previously set objectives. The clinical situations were problematic even for experienced clinicians, either because there were not enough data or the situations were ambiguous. There were several options for diagnosis, investigation, or treatment. The items (questions) were based on a panel of questions that an experienced clinician would consider relevant to this type of clinical setting. The item was consistent with the presentation of relevant options and new data (not described in the vignette). The task for the student was to determine the effect these new data on the status of the option. The resident’s task was to assess, using a 5-point Likert scale, the influence of this new element on the diagnostic hypothesis, the plan for investigation, or the treatment. The different points on the scale corresponded to positive values (the option was enhanced by the new data), neutral values (the data did not change the status of the option), or negative values (this option was ruled out by the data). The scoring system was based on the principle that any answer given by one expert had an intrinsic value, even if that answer did not coincide with those of other experts. In the present study, a group of 13 anesthesiologist practitioners regularly involved in the management of traumatic hemorrhagic shock formed the expert panel. The principles of SCT are that for each item, the answer entitled the resident to a credit corresponding to the number of experts who had chosen it. All items had the same maximum credit, and raw scores were transformed proportionally to obtain a one-point credit for the answer that was chosen by most experts. Other choices received a partial credit. Thus, to calculate the scores, all results were divided by the number of individuals who had given answers chosen by the largest number of respondents. The total score for the test was the sum of all credits earned for each item. The total score was then transformed into a percentage score. An automatic correction software (freely accessible on the website of the University of Montreal) was used for scoring [25]. Each MCQ was worth one point, and it was possible for an MCQ to have several correct answers. To obtain a point for an MCQ, the resident had to tick all the correct answers and none of the incorrect ones. Otherwise, the student did not receive any points. The final rating was based on the total number of proposed MCQs.

### Statistical Analysis

With regard to our previous publication on the use of SCT by anesthesiology residents, we assumed that a difference of 6% between the two groups would be clinically significant [12,24]. Based on these findings, assuming that the SD was the same between the populations and using a power of 0.90 with a level of statistical significance at .05, it was estimated that 22 students should be analyzed in each group. A randomized study on e-learning showed that about a quarter of the students included do not participate or are lost to follow-up [26]. Based on these findings, it was estimated that a minimum of 28 students should be included in each group to be able to analyze 22 students.

The values are presented as number and percentage values for qualitative variables, as mean and SDs for quantitative variables with a normal distribution, and as median and interquartile range for quantitative variables with a non-normal distribution. Residents who did not respond to the final evaluation were excluded from the final analysis (lack of analyzable parameters). After performing a Shapiro-Wilk normality test, the quantitative variables were compared using a Student *t* test (if the distribution was normal) or a Mann-Whitney test (if the distribution was not normal). The qualitative variables were analyzed using a Fischer or a chi-square test. The significance threshold was set at .05. All statistics were analyzed using GraphPad PRISM software (v 5.0; GraphPad Software Inc, San Diego, CA).

## Results

### Residents’ Characteristics

Among 142 eligible anesthesiology residents, 62 (44%) agreed to participate and were randomized as follows: 32 to the WhatsApp group and 30 to the control group. Their main characteristics are summarized in Table 2. Two students randomized to the WhatsApp group were excluded after randomization. The first withdrew from the study for personal reasons, and the second was excluded following failure to download WhatsApp.

### Results of the Script Concordance Tests and Multiple-Choice Questions

**Figure 1:**
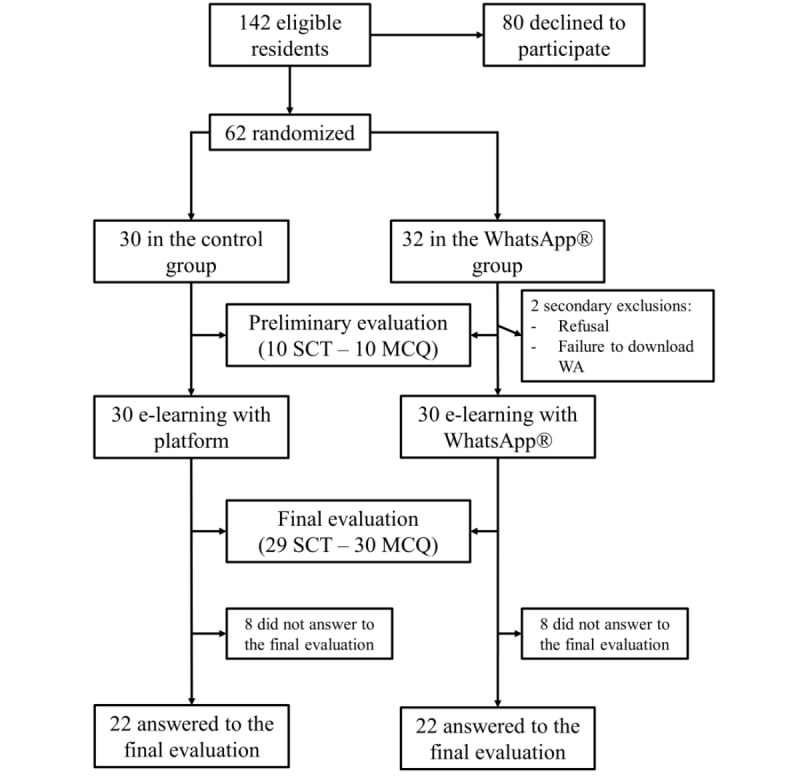
Flowchart of the study. e-learning: electronic learning; MCQ: multiple-choice question; SCT: script concordance test.

The lessons took place from March 12 to 30, 2018. For final evaluation, SCTs including 12 scenarios for a total of 36 items were submitted to a panel of 13 experts. Thereafter, 7 items of the SCT were excluded (not enough variability in replies), leaving 29 items of SCT spread over 12 clinical situations. According to the recommendations of Lubarsky et al, we optimized SCT by performing a post-hoc analysis [18]. Items with high variability, low variability, or binomial responses were excluded. We obtained a final version with 10 scenarios and 24 items. After this optimization, Cronbach coefficient alpha was .55. In the WhatsApp group, 20 residents answered the preliminary evaluation, 1 resident who responded to the preliminary evaluation did not answer the final evaluation, and 3 residents who did not respond to the preliminary evaluation answered the final evaluation. In the control group, 22 residents answered the preliminary evaluation, 1 resident who responded to the preliminary evaluation did not answer the final evaluation, and 1 resident who did not answer the preliminary evaluation answered the final evaluation. There was no demographic disparity between the residents who answered and those who did not answer the final evaluation. Their main characteristics are summarized in Tables 3 and 4. The flow chart of the study is presented in Figure 1.

On the preliminary evaluation (before teaching), there was no significant difference between the WhatsApp and control groups for SCT (64% [SD 7%] vs 62% [SD 6%]; *P*=.41) or MCQ (8/10 [SD 1] vs 7/10 [SD 2]; *P*=.33), showing no difference in clinical reasoning or medical knowledge. For the final evaluation (after teaching), we found a significant difference between the WhatsApp and control groups for SCT (60% [SD 9%] vs 68% [SD 11%]; *P*=.006) but not for MCQs (18/30 [SD 4] vs 16/30 [SD 4]; *P*=.22). In the WhatsApp group, there was no difference in the SCT between the initial evaluation and the final evaluation (*P*=.14). In the control group, the SCT scores of the final evaluation were significantly higher than those of the initial evaluation (*P*=.02).

**Table 2:** Demographic characteristics of the residents.

Characteristic		Control group (n=30), n (%)	WhatsApp group (n=32), n (%)
**Year of residency**			
	First	14 (47)	14 (44)
	Second	16 (53)	18 (56)
**Sex**			
	Male	17 (57)	23 (72)
	Female	13 (43)	9 (28)
**University hospital**			
	Rouen	10 (33)	11 (34)
	Lille	10 (33)	11 (34)
	Caen	6 (20)	5 (16)
	Amiens	4 (14)	5 (16)

**Table 3:** Demographic characteristics of residents who answered the final evaluation.

Characteristic		Control group (n=22), n (%)	WhatsApp group (n=22), n (%)
**Year of residency**			
	First	9 (41)	9 (41)
	Second	13 (59)	13 (59)
**Sex**			
	Male	14 (64)	16 (73)
	Female	8 (36)	6 (27)
**University hospital**			
	Rouen	10 (45)	10 (45)
	Lille	6 (27)	5 (23)
	Caen	5 (23)	4 (18)
	Amiens	1 (5)	2 (10)

**Table 4:** Demographic characteristics of residents who did not answer the final evaluation.

Characteristic		Control group (n=8), n (%)	WhatsApp group (n=8), n (%)
**Year of residency**			
	First	5 (63)	3 (37)
	Second	3 (37)	5 (63)
**Sex**			
	Male	3 (37)	5 (63)
	Female	5 (63)	3 (37)
**University hospital**			
	Rouen	0 (0)	0 (0)
	Lille	4 (50)	4 (50)
	Caen	1 (13)	1 (13)
	Amiens	3 (37)	3 (37)

### Use of WhatsApp and Residents’ Satisfaction

The residents of the two groups who filled the final evaluation were asked to fill an online satisfaction questionnaire. Twenty (67%) residents in the WhatsApp group and 13 (43%) residents in the control group answered this questionnaire. All the scores from the satisfaction evaluation had a non-normal distribution. There was a difference between the WhatsApp and control groups, with the WhatsApp group showing a better global satisfaction rate (8/10 [interquartile range, 8-9] vs 8/10 [interquartile range 8-8]; *P*=.049), a better feeling that the lessons met the learning objectives (10/10 [interquartile range 8-10] vs 8/10 [interquartile range 7-10]; *P*=.03), and a feeling that the teachers were more available (10/10 [interquartile range 9-10] vs 9/10 [interquartile range 8-10]; *P*=.007). We found no differences between the WhatsApp and control groups in terms of the perceived quality of educational materials (9/10 [interquartile range 8-10] vs 8/10 [interquartile range 8-10]; *P*=.15), the usefulness and relevance of clinical cases (10/10 [interquartile range 8-10] vs 9/10 [interquartile range 7-10]; *P*=.40), the quantity of teaching documents used by the residents (in the WhatsApp group, 14 residents [70%] used more than 50% of the documents and 6 [30%] used less than 50% of the documents; in the control group, 10 residents [77%] used more than 50% of the documents and 3 [23%] used less than 50% of the documents; *P*=.66), or the time spent working on the program (in the WhatsApp group, 2 residents [10%] spent between 5 h and 10 h and 18 [90%] spent between 1 h and 5 h; in the control group, 4 residents [31%] spent between 5 h and 10 h and 9 [69%] spent between 1 h and 5 h; *P*=.18). Textbox 1 presents quotes from the free comments section of the satisfaction questionnaire of the WhatsApp group.

**Quotes from the free comments section of the satisfaction questionnaire (WhatsApp group residents).**

“For participation to the Friday clinical case it depends on the availability of everyone. The fact that it is on whatsapp makes it easier to communicate and ask questions. Having notifications is more motivating to consult documents than on a platform.”

“Having what's app notifications allows me to be more assiduous, the possibility to ask questions directly in the conversation is a big advantage, it sometimes allows small discussions, so very useful. Great classes, interesting cases, and not feeling evaluated is fun. Suggestion: a new session.”

“The documents were very well done, difficult on Friday to answer all the questions of the clinical cases online according to our occupations in the ward.”

“Very good idea to teach via WhatsApp, which allows to be informed quickly of the presence of new educational documents and to have regular reminder shots since the notifications are displayed. Doing clinical cases on the application during a day with the participation of several people is very instructive. The only problem is that the documents are difficult to consult on a small telephone, perhaps it would be necessary to adapt the documents in the form of slides format telephone. Otherwise it was great! High quality educational documents. Thank you!”

“Interesting to be able to consult documents via whatsapp. As far as Friday clinical cases are concerned, it is quite difficult to switch between ward presence or other obligations and whatsapp.”

“Very nice project. I think it's useful to have cards per whatsapp but the flow was too high: 4 documents per day, we end up having too much delay in the readings.”

“The idea is good but it's quite laborious to read lessons on a mobile phone, especially long pdf. The well ventilated and clear synthetic dcouments [sic] are on the other hand interesting. It is also interesting to be able to ask questions directly and get quick answers. But it can't replace classical education.”

## Discussion

### Principal Results

This randomized, multicenter study is the first to focus on the impact of WhatsApp on clinical reasoning in medical students. We found that the use of WhatsApp, instead of a traditional e-learning platform, to teach a specific topic was associated with worse clinical reasoning despite better global appreciation.

### Comparison With Prior Work

Several recent studies have reported the potential interest of specific smartphone apps in medical education, but our objective was to assess the interest of a very widely used nonmedical app (thus easily usable by all) for teaching [27-29]. Given that WhatsApp allows interaction between teachers and students, with the possibility of discussing clinical cases, we believed its use would improve medical reasoning, as previously described for face-to-face practice exchange groups [12]. We did not find any difference in the global amount of work or the number of educational documents consulted, which is consistent with similar personal work between the two groups. It has been shown that e-learning methods improve the medical knowledge of health care professionals [28]. The absence of a difference in the medical knowledge assessed by MCQs shows that the weakness of clinical reasoning related to WhatsApp is not related to less knowledge of the subject. We can therefore assume that this decrease in the quality of reasoning is directly related to WhatsApp or the use of a smartphone. It is likely that reading on WhatsApp between two other activities was less effective than time spent solely on an e-learning platform. A recent study showed that a smartphone app dedicated to teaching medical students Dermatology, in combination with traditional teaching, improved medical knowledge measured by MCQs [27]. Although we did not find any improvement in medical knowledge in our work, the smartphone was seen as an alternative to conventional e-learning and not as a complement. It is interesting to note that in the literature, most of the educational benefits reported with smartphone use stem from very “visual” specialties (Dermatology or Pathology) and that this tool, which allows easy communication of iconography, is probably more relevant in this context than in “less visual” medical specialties [10,27].

Residents pointed out two limitations: the difficulty of participating in clinical cases on Friday in parallel with their usual activities and the difficulty in referring to documents on small smartphone screens. Unlike for practice exchange groups, there was no time dedicated specifically to clinical case resolution on WhatsApp, and residents had to respond in addition to their usual activities [12]. This probably favored a multitasking activity with a difficulty to focus on the pedagogical content. However, it is interesting to note that the comments from WhatsApp residents were very positive, with a higher overall satisfaction rating. The novelty and originality of the concept probably contributed to this satisfaction, but it underlines the fact that the students were not aware of the possible negative impact of the use of WhatsApp. A recent randomized pedagogic study assessed the impact of learning modules using m-learning on knowledge gain, skill gain, and satisfaction for otorhinolaryngology and head and neck surgery disorders in undergraduate medical students [28]. Despite the absence of differences in knowledge gain in the mobile interactive multimedia group, satisfaction was higher in the mobile group (like in our cohort). Therefore, we can assume that our data are concordant with the literature on m-learning.

Finally, the daily use of WhatsApp for medical education probably contributes to the dispersion of attention linked to the use of the smartphone. In view of these results, it does not seem justified to continue to develop WhatsApp for teaching medical reasoning to medical residents. However, the targeted use of WhatsApp with other educational objectives (eg, medical imaging or video) remains to be evaluated and should be the subject of future randomized studies. It is known that blended learning can have a beneficial effect on knowledge acquisition in health professions [30]. Thus, it might also be interesting to study the use of WhatsApp as a complement to another form of teaching. Given the increasing use of smartphones by health workers, it also seems appropriate to consider future work to assess the quality of clinical reasoning between two populations of physicians with or without usual smartphone use in hospitals.

### Limitations

Our study has several limitations. First, the Cronbach coefficient alpha in our SCT evaluation was low. The minimum coefficient usually retained for normative evaluations is 0.7, but in our work the evaluation was only formative and integrated into teaching. The limited number of SCTs probably explains this low coefficient. However, teaching in a specific and specialized area made it difficult to find at least 60 SCTs (as is usually recommended) without redundancy [18]. Second, residents’ participation in our work was limited: Only 62 of 142 residents participated. As previously observed, self-training with e-learning is impacted by a significant dropout rate [26]. In our work, only 22 of the 30 residents participated in the final evaluation. As this teaching was optional, participation in our study represented additional personal work for the residents. It is therefore possible that the majority of residents were discouraged by this prospect. In addition, residents without smartphones or those who did not wish to use WhatsApp logically refused to participate. Third, we could not prevent cross-communication among students while they answered the SCTs and MCQs, and the residents could have communicated with each other during the final evaluation. The fact that this evaluation was not sanctioned and had no value, as it was not integrated into usual teaching methods, probably limited this communication. Finally, we did not use a prevalidated questionnaire to measure satisfaction. As we wanted to evaluate specific points related to the use of WhatsApp in our population, we created a new dedicated questionnaire, but this choice made it more difficult to compare our satisfaction results to those of others.

### Conclusions

Compared to traditional e-learning, the use of WhatsApp as an m-learning method for residents teaching is associated with worse clinical reasoning despite better global appreciation. The use of the WhatsApp app probably contributes to the dispersion of attention linked to the use of the smartphone.  

## Appendix

Multimedia Appendix 1 Teaching documents especially prepared for easy readability on a smartphone (in French with English translation).

Multimedia Appendix 2 "Step-by-step" clinical cases (in French with English translation).

Multimedia Appendix 3 Several examples of the use of WhatsApp for learning purposes during the protocol (screenshots in French with English translation).

Multimedia Appendix 4 Script concordance test used for the final evaluation (in French with English translation).

Multimedia Appendix 5 Multiple-choice questions used for the final evaluation (in French with English translation).

Multimedia Appendix 6 Online satisfaction questionnaire (in French with English translation).

Multimedia Appendix 7 CONSORT‐EHEALTH checklist (V 1.6.1).

## Abbreviations

e-learning: electronic learning
MCQ: multiple-choice questions
m-learning: mobile learning
SCT: script concordance test
