# Supplementary material for: Use of the Smartphone App WhatsApp as an E-Learning Method for Medical Residents: Multicenter Controlled Randomized Trial
Source: JMIR Mhealth Uhealth. 2019 Apr 9;7(4):e12825. doi: 10.2196/12825 (PMC6477573; doi:10.2196/12825)
Supplement: Multimedia Appendix 1 [file mhealth_v7i4e12825_app1.pdf]

Endothélium sain : *thromborésistant = protège de l'activation des plaquettes (prostacycline PgI2 puissant antiagrégant plaquettaire), régule négativement la coagulation (glycosaminoglycanes et* ***thrombomoduline TM****), synthétise protéines fibrinolytiques (t-PA/ PAI-1)*
Propriétés ***antiagrégantes, anticoagulantes et pro fibrinolytiques*** *de l'endothélium sain*

HÉMOSTASE PRIMAIRE = obturation de la brèche vasculaire par un clou plaquettaire

Atteinte endothéliale ou brèche vasculaire => vasoconstriction immédiate réflexe

- ↘ pertes sanguines par ↘ flux sanguin
- Interaction + efficace des plaquettes circulantes avec la matrice sous-endothéliale (collagène, facteur Willebrand)

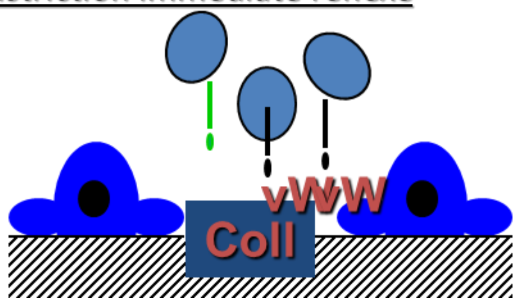

Adhésion des plaquettes au sous-endothélium

Exposition du facteur Willebrand

Fixation des plaquettes sur le facteur Willebrand grâce à la **GPIbIX plaquettaire**

Fixation des plaquettes sur le collagène par la **GPIaIIa plaquettaire**

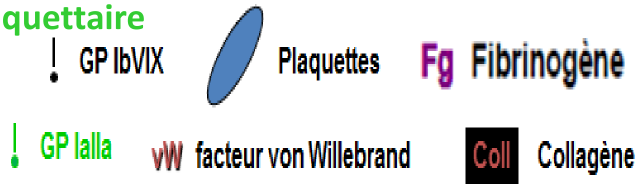

Activation et sécrétion des plaquettes

Changement de morphologie plaquettes discoïdes => sphériques
+ émission de pseudopodes
+ sécrétion du contenu des granules plaquettaires :
- ADP (pro agrégant)
- sérotonine (pro agrégant et VC)
- **fibrinogène Fg**, facteur V, **calcium**

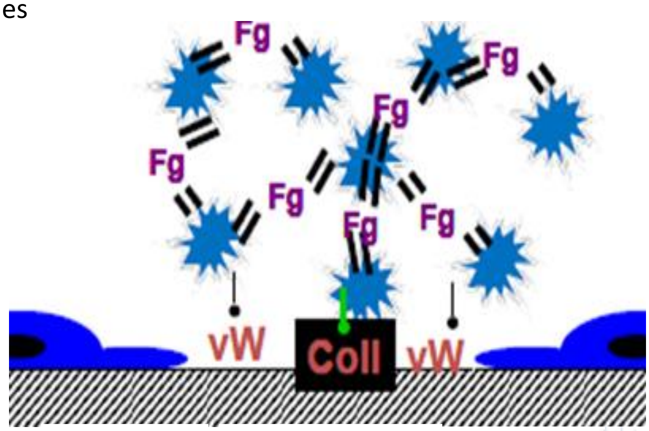

Mise à la surface de GP IIbIIIa
Flipflop membranaire avec passage à l'extérieur des phospholipides anioniques = activation du cycle des prostaglandines = pro-agrégant (TXA2)

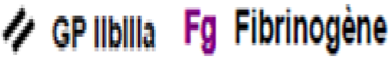

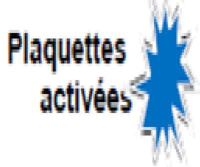

Agrégation des plaquettes

Plaquettes activées attirent (TXA2) et activent (ADP) d'autres plaquettes
Elles s'accrochent les unes aux autres par le **fibrinogène Fg** et GP IIbIIIa
en présence de **calcium**

Agrégat plaquettaire arrête **partiellement l'hémorragie**.
Clou plaquettaire arrête un saignement que dans un vaisseau de tout petit calibre (capillaire).

Dès que l'on est dans un vaisseau de plus gros calibre, l'intervention de la **COAGULATION** est nécessaire à la solidification du caillot par **transformation du fibrinogène en fibrine**

COAGULATION : Voie intrinsèque/ Voie extrinsèque / Tronc commun

Mécanisme initiateur qui résulte de la lésion vasculaire

Clou plaquettaire ⇒ Réseau de fibrine insoluble

Mécanismes activateurs : les facteurs de la coagulation (synthèse hépatique) sont activés par clivage enzymatique (pro-enzymes)

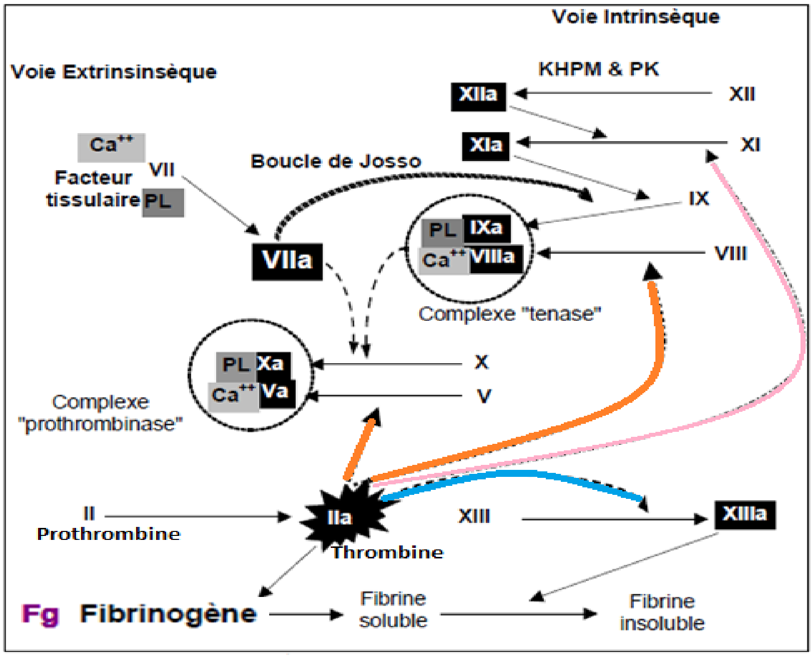

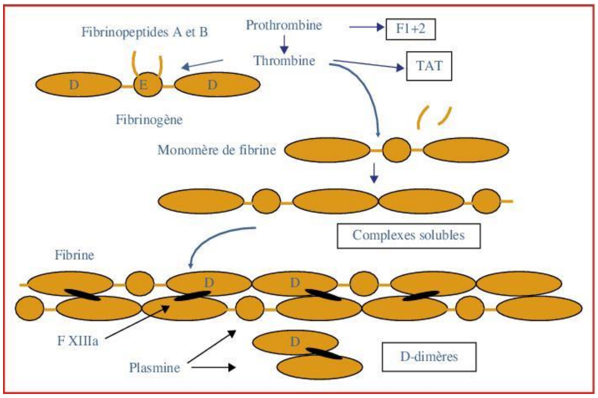

VOIE EXTRINSEQUE : lésion endothélium démasque Facteur tissulaire

Facteur tissulaire + VII + Ca^{2+} ⇒ VIIa

VIIa + Ca^{2+} + PL plaquettes activées + X ⇒ Xa

VOIE INTRINSEQUE : collagène du sous-endothélium à nu entre en contact avec facteurs XII et XI (dits "facteurs contacts")

XII + KHPM + kallicréine ⇒ XIIa

XIIa active XI ⇒ XIa

XIa active IX ⇒ IXa

IXa + VIII + Ca^{2+} + PL plaquettes activées + X ⇒ Xa

TRONC COMMUN : au niveau des phospholipides des plaquettes activées en présence de Ca++

Xa + V + II (prothrombine) ⇒ IIa (thrombine)

IIa protéolyse Fg **Fibrinogène** en monomères de fibrine

ces monomères s'associent en polymères ⇒ fibrine soluble

IIa + XIII + Ca^{2+} ⇒ XIIIa

XIIIa va stabiliser la fibrine soluble en réseau insoluble

Premières traces IIa (thrombine) activent V et VIII ⇒ Va et VIIIa ⇒ X 10 000 génération de thrombine

caillot formé reste en place 10-14j le temps de la cicatrisation puis fibrinolyse...

Mécanismes inhibiteurs de la COAGULATION

Comment le caillot de fibrine insoluble est-il limité à la brèche vasculaire?

Thrombine sur le caillot de fibrine = **procoagulante** + auto-amplification de sa génération

Thrombine en excès hors du caillot de fibrine = **anticoagulante** par auto inhibition (AT et système PC/PS).

L'antithrombine (AT) = principal inhibiteur de la coagulation

La thrombine en excès du caillot de fibrine va être neutralisée par l'antithrombine AT.
Formation d'un [**complexe antithrombine-thrombine**] **très stable = inhibition irréversible.**

De plus : l'AT peut aussi neutraliser l'activité de la majorité des autres enzymes de la coagulation : Xa, IXa, XIa, XIIa, kallikréine. Elle ne neutralise pas le VIIa

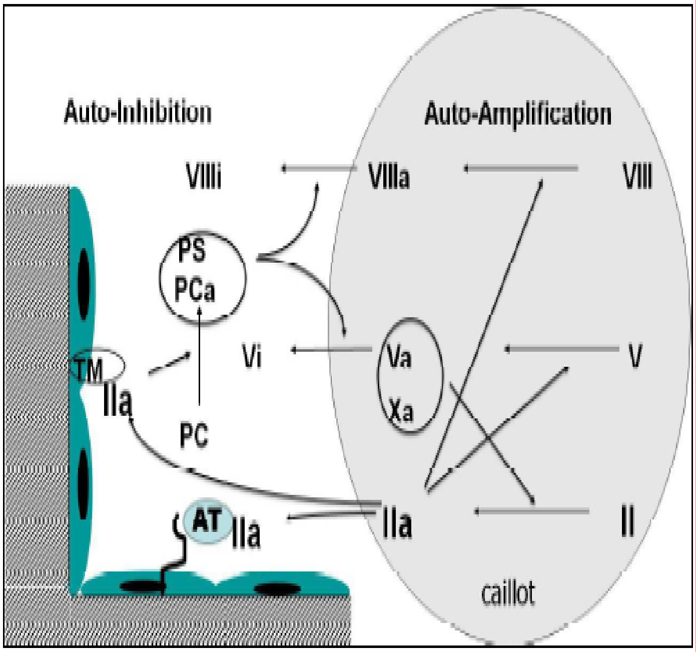

Le système des Protéines C et S

La thrombine en excès du caillot de fibrine se fixe sur un récepteur très spécifique de l'endothélium: la thrombomoduline TM.

Après fixation sur la TM, la thrombine et perd ses propriétés procoagulantes et va être **capable d'activer la protéine C.**

La **protéine C activée** en présence de phospholipides, de calcium et de son cofacteur la protéine S (PS), se fixe sur le caillot et y **dégrade les facteurs Va et VIIIa**, **décélérant la coagulation**.

THROMBINE ANTICOAGULANTE
HORS DU CAILLOT ="DECELERATION"

THROMBINE PROCOAGULANTE
DANS LE CAILLOT = "ACCELERATION"

Système inhibiteur de la voie extrinsèque : l'inhibiteur de la voie du facteur tissulaire (TFPI)

1/ TFPI se fixe au facteur Xa
2/ puis fixation au complexe [Facteur tissulaire – FVIIa]
=> complexe quaternaire.
Dès lors le TFPI **inhibe rapidement l'activité du [complexe FT/VIIa].**

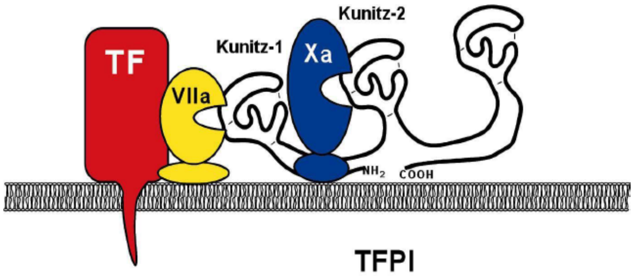

TFPI

- Inhibe le Xa et le complexe TF-VIIa.

Se complexe au Xa, complexe Xa-TFPI puis se lie au complexe membranaire TF-VIIa
⇨ complexe quadrimoléculaire ⇨ neutralisation du VIIa.

FIBRINOLYSE = Dégradation du caillot de fibrine intravasculaire par la Plasmine

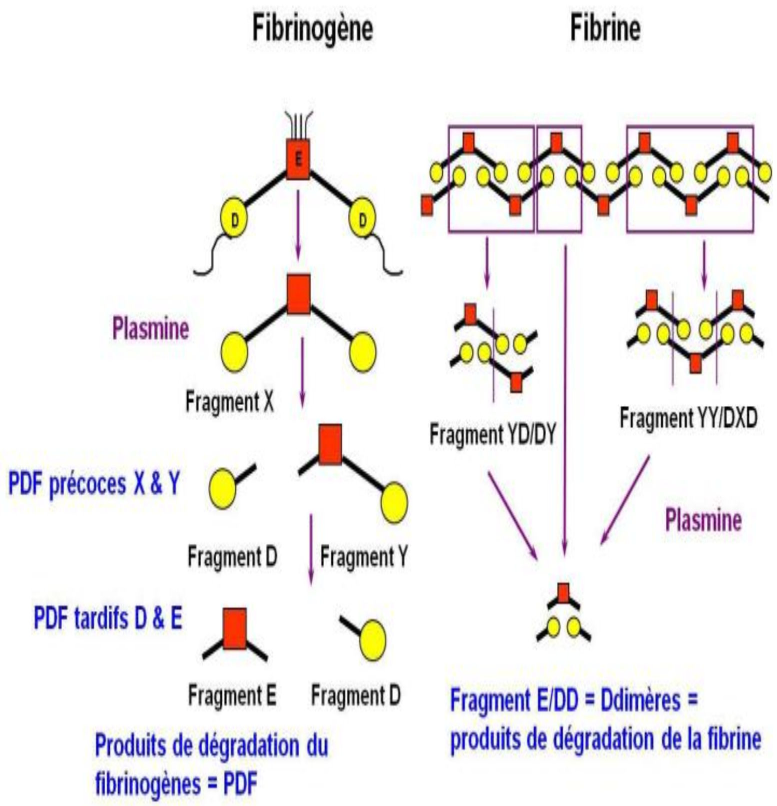

Dégradation de la fibrine par la plasmine

La plasmine découpe les filaments de fibrine **en produits dits de dégradation de la fibrine**, parmi eux les Ddimères

Dégradation du fibrinogène par la plasmine

La plasmine est aussi capable de dégrader le fibrinogène avec apparition de **produits de dégradation du fibrinogène (PDF)**

HORS il n'y a pas de plasmine circulante à l'état physiologique. La plasmine résulte de l'activation du plasminogène (inactif).

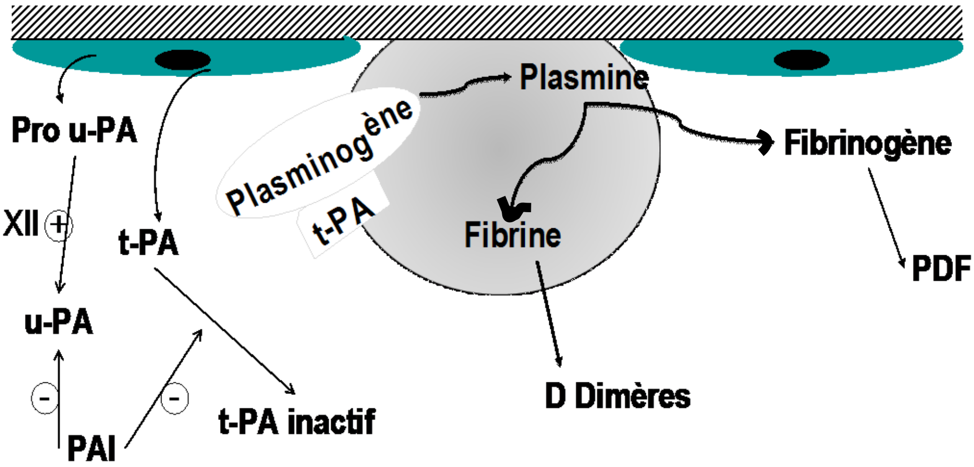

Deux activateurs principaux du plasminogène :

t-PA = activateur tissulaire du plasminogène

- Synthétisé par la cellule endothéliale
- Non efficace dans la circulation car il est complexé à son inhibiteur le PAI-1

En revanche, comme il a une très grande affinité pour la fibrine, il se fixe lui aussi sur la fibrine du caillot. Ainsi il échappe à son inhibiteur et au sein du caillot, il peut transformer le plasminogène en plasmine active.

pro-urokinase (Pro u-PA) est un second système d'activation du plasminogène.

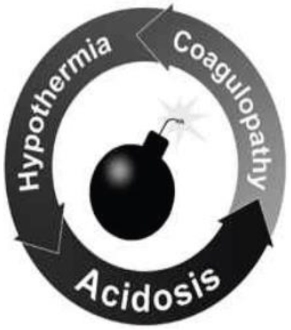

Traumatisme + Hypoperfusion (état de choc)

« Trauma-induced coagulopathy »

Gravité du traumatisme et durée du choc = Gravité coagulopathie

= **HYPOCOAGULATION** et **HYPERFIBRINOLYSE**

« Only patients who are in shock are coagulopathic on admission »

Hypothèse 1 : Coagulopathie de consommation

1 / La blessure expose le collagène de la matrice sous-endothéliale et le **facteur Willebrand** aux Glycoprotéines plaquettaires (GP)

↓

ACTIVATION, AGREGATION PLAQUETTAIRE = **clou plaquettaire** (hémostase primaire)

2/ La lésion expose également le **Facteur Tissulaire** à la circulation (activation voie extrinsèque)

↓

GENERATION DE THROMBINE (IIa) + formation du **caillot de fibrine** (coagulation)

+ AMPLIFICATION de la génération de thrombine et de la coagulation

↓

Consommation des facteurs de la coagulation => HYPOCOAGULATION
(les facteurs les plus appauvris sont le fibrinogène et facteur V)

3/ LIBERATION de **l'activateur tissulaire du plasminogène t-PA** : le plasminogène est transformé en plasmine dans la circulation = **HYPERFIBRINOLYSE**

Hypothèse 2 : Activation de la Protéine C (PC)

↗ de L'expression de la **thrombomoduline TM**

→ Liaison de la thrombine à la TM

→ **ACTIVATION de la PC**

→ Inhibition facteurs Va et VIIIa = **HYPOCOAGULATION**

→ Inhibition de l'inhibiteur de l'activateur du plasminogène (PAI-1) = **PRO-FIBRINOLYSE**

Transfusion : Que prescrire en urgence si choc hémorragique ?

VOLUME SANGUIN TOTAL : femme = 60-65 mL/kg et homme 70-75 mL/kg
1g/dL = 3% hématocrite // 1 CGR = 250 mL environ à 60% hématocrite
De façon très schématique, la transfusion 1 CGR augmente l'hémoglobine de 1 g/dL

En l'absence de données immuno-hématologiques :

TRANSFUSION CGR O RH:1 KEL:-1 soit O positif

Sauf femme de la naissance jusqu'à la fin de la période procréatrice (= CGR O négatif)

En cas de transfusion massive, la disponibilité des CGR prime sur la compatibilité dans les systèmes de groupes sanguins hors système ABO

Objectif d'hémoglobine entre **7 et 9 g/dL**
Particularités : **Coronarien, BBloquants, Traumatisé crânien : 9-10 g/dL**

Transfuser le PFC en association avec les CGR avec un **ratio PFC:CGR compris entre 1/ 2 et 1/1**

PLASMA FRAIS CONGELE

Débuter la transfusion de **plasma rapidement**, idéalement **en même temps que celle des CGR, AVANT** l'obtention des résultats biologiques

En cas d'indisponibilité de plasma isogroupe ABO OU en situation d'urgence vitale : Le **plasma AB** est utilisable quel que soit le groupe du receveur

Transfusion plaquettaire précoce, généralement lors de la **deuxième prescription transfusionnelle**

Objectif : Numération des plaquettes **au-dessus de 50 G/L**

↓

- Si persistance du saignement
- Si traumatisme crânien associé

↓

Objectif : Numération des plaquettes **au-dessus de 100 G/L**

Faut-il monitorer la concentration de calcium ionisé chez les patients en choc hémorragique ?

TRANSFUSION MASSIVE

⇩

Une hypocalcémie ionisée peut survenir lors d'une transfusion massive en raison du citrate utilisé comme anticoagulant dans les produits sanguins labiles (surtout le PFC). L'utilisation de colloïdes peut aussi jouer un rôle.

+

L'existence d'un bas-débit sanguin hépatique au cours de l'état de choc entrave le métabolisme du citrate.

=

Le **monitorage** de la concentration de **calcium ionisé** est recommandé en cas de transfusion massive.

La **concentration de calcium ionisé doit être maintenue > 0,9 mmol/L** par un apport de chlorure de calcium circulant sur une voie veineuse indépendante de la transfusion.

CAR

La baisse du calcium ionisé, biologiquement actif, expose à une aggravation de la **défaillance cardiovasculaire voire un arrêt circulatoire**.

Il pourrait aggraver aussi une **défaillance hémostatique.**

EXACYL® : Acide tranexamique et choc hémorragique

Médicament ANTI- fibrinolytique Analogue synthétique de la lysine = **Diminution de la transformation de plasminogène en plasmine**

Demi-vie d'élimination plasmatique courte : 80–120 min
après injection intraveineuse

Pas d'augmentation du risque de thrombose veineuse ou artérielle

Dès que possible à la dose **de 1 g en bolus intraveineux en 10 min**
suivi de 1 g perfusé sur 8 h
chez les patients traumatisés en choc hémorragique.

L'administration d'acide tranexamique ne doit pas être initiée au-delà de la 3e heure suivant la survenue d'un traumatisme avec choc hémorragique.
Augmentation de la mortalité si administré après 3H.

Réf : [Effects of tranexamic acid on death, vascular occlusive events, and blood transfusion in trauma patients with significant haemorrhage (CRASH-2): a randomised, placebo-controlled trial – Lancet - 2010]
Réf : [The importance of early treatment with tranexamic acid in bleeding trauma patients: an exploratory analysis of the CRASH-2 randomised controlled trial – Lancet - 2011]

Produits dérivés du sang

**Fibrinogène** CLOTTAFACT®, LFB; RIASTAP®, CSL Behring<br><br>Substrat de la prothrombine (IIa) qui va être transformé en fibrine = **stabilisation du caillot de fibrine**<br>- **3 g chez un adulte de 70 kg**<br>- PAS de bolus<br>- IVL sur 10-20 min<br>- sur VVP (risque accumulation fibrinogène dans OG si VVC)	Recommandée en cas de **fibrinogénémie < ou = 1,5 g/L**, ou de paramètres thromboélastographiques (-métriques) de déficit en fibrinogène fonctionnel.	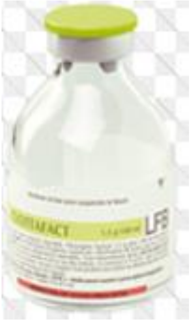
**CCP non activés** KANOKAD® LFB; OCTAPLEX® Octapharma ; CONFIDEX®, CSL Behring<br>**50 U/kg**<br>Concentré des facteurs :<br>- **II** (prothrombine)<br>- **VII** (proconvertine)<br>- **IX** (facteur antihémophilique B)<br>- **X** (facteur Stuart)	Indication : **Uniquement** en cas de choc hémorragique survenant **chez un patient traité par AVK**<br><br>Certaines équipes européennes utilisent les CCP non activés en substitut de la transfusion de PFC dans le cadre du choc hémorragique en dehors de tout traitement par AVK. Ces pratiques ne sont pas recommandées à l'heure actuelle en France.	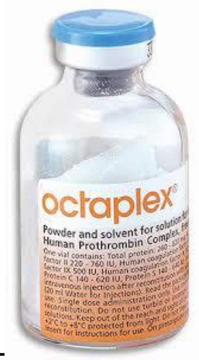
**CCP activés** Factor Eight Inhibitor Bypassing Activity ou FEIBA®, Baxter<br>**30–50 U/kg**<br>Concentré des facteurs :<br>- **II** (prothrombine)<br>- **VII** (proconvertine)<br>- **IX** (facteur antihémophilique B)<br>- **X** (facteur Stuart)	Indication : **Uniquement** en cas de choc hémorragique survenant **chez un patient traité par anticoagulants oraux directs** (dabigatran, rivaroxaban, apixaban)<br><br>= neutralisation immédiate de l'effet anticoagulant	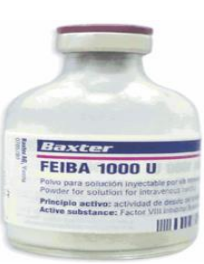
**Facteur VII recombiné activé** NOVOSEVEN®, Novo Nordisk<br><br>La posologie initiale recommandée est de 80 à 200 µg/kg.	En cas de choc hémorragique, le **rFVIIa ne doit PAS être utilisé** en 1re intention.<br><br>Il ne doit être envisagé **que si le saignement ne peut pas être contrôlé malgré :**<br>- interventions hémostatiques mécaniques ET<br>- utilisation d'acide tranexamique ET<br>- la transfusion de produits sanguins labiles ET<br>- la transfusion de concentrés de fibrinogène ET<br>- la correction d'une hypothermie sévère ET<br>- la correction d'une acidose sévère	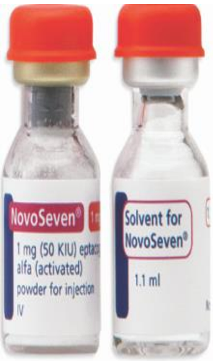

Accueil Polytraumatisé
Bilan lésionnel et Orientation

Patient qui a souffert d'un traumatisme violent quelles que soient les lésions apparentes : **1 critère de Vittel = Polytraumatisé GRAVE**

Examen initial du patient	Glasgow < 13<br>$SaO_2 < 90\%$<br>Pression artérielle systolique < 90 mmHg
Circonstances de l'accident	Victime éjectée – projetée - écrasée<br>Décès dans l'accident<br>Chute > 6m – explosions - blast
Prise en charge préhospitalière	Ventilation assistée<br>Remplissage > 1000 mL<br>Catécholamines
Lésions observées ou suspectées	Trauma pénétrant – volet thoracique – trauma bassin<br>Amputation de membre – ischémie aiguë de membre<br>Brûlure – suspicion de lésion médullaire
Caractéristiques du patient	Age > 65 ans<br>Grossesse au 2ème et 3ème trimestre<br>Tares associées

ACCUEIL

- → Instabilité extrême → Chirurgie / Artériographie
- → Stabilisation par la réanimation → Scanner corps entier
- → Stabilité hémodynamique → Scanner corps entier

Chirurgie / Artériographie ↔ Scanner corps entier

Monitoring
2 VVP bon calibre
Bilan sanguin
FAST ECHO
Radiographie thoracique
Radiographie de bassin
Lutte contre hypothermie

PRIORITE dans la prise en charge des défaillances :
1/ HEMORRAGIE 2/ CRANE 3/ ORTHOPEDIE

Objectifs de PA, Expansion volémique, Amines

De façon très schématique :
« Tachycardie à partir d'une perte de 20-25% masse sanguine et Hypotension à partir d'une perte de 40% masse sanguine »

Tant que le saignement n'est pas contrôlé : **Limiter le remplissage** au **strict maintien des objectifs de PA**

En l'absence de TC grave

Tolérer Hypotension artérielle
objectif **PAS 80–90 mmHg**
ou
PAM 60– 65 mmHg

Si TC grave (Coma Glasgow Score inf ou égal à 8)

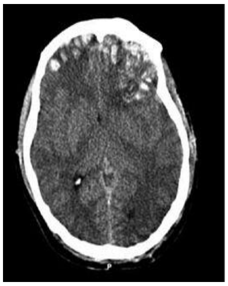

PAM > 80 mmHg, avant de disposer d'un monitorage cérébral, en dépit du risque d'aggravation du saignement

Pas de solutés hypotoniques

Pré hospitalier
Intra-osseux >> KTC si VVP impossible

1ere intention
Solutés cristalloïdes
Ne pas utiliser l'albumine

Si VVP disponible rapidement
Pose KTc ne retarde pas le traitement étiologique et la stabilisation hémodynamique

HEA si **cristalloïdes seuls** sont **insuffisants** pour maintenir la volémie.
Dose **la plus faible possible**
Durée **la plus courte possible**

Semi-synthétiques? Pas d'études

Pour apprécier le degré d'hypoperfusion et d'hypoxie tissulaire : suivre l'évolution de la ***[lactate] artériel***

Administrer un vasopresseur si hypo TA persistante (PAS < 80 mmHg)
après début du remplissage vasculaire.
Noradrénaline en première intention
L'administration de Noradrénaline est recommandée sur une VVC. Cependant, ***dans un contexte d'urgence****, et dans l'attente de la pose d'une VVC,*
il est recommandé d'utiliser une VVP

Traumatisme du bassin et CHOC HEMORRAGIQUE

CHOC HEMORRAGIQUE + Traumatisme du bassin
+ épanchement péritonéal à l'échographie (FAST ECHO)

Patient instable malgré remplissage vasculaire et catécholamines

→ Hémopéritoine massif → Laparotomie → Artériographie

→ Épanchement minime à modéré → Artériographie

Patient stabilisé par remplissage vasculaire +/- catécholamines

→ TDM injectée → Artériographie

PENSER : **Ceinture contention pelvienne dès le pré hospitalier** pour toute fracture cliniquement instable du bassin

---

CHOC HEMORRAGIQUE + Traumatisme du bassin
SANS épanchement péritonéal massif à l'échographie

Patient instable malgré remplissage vasculaire et catécholamines

→ Artériographie +/- Embolisation en Radiologie interventionnelle

Patient stabilisé par remplissage vasculaire +/- catécholamines

→ TDM injectée avec **hémorragie active dans le pelvis** sur TDM

Artériographie +/- Embolisation en Radiologie interventionnelle
= QUEL QUE SOIT L'ETAT HEMODYNAMIQUE
(recommandations professionnelles EAST 2011)
Hémostase chirurgicale difficile, et délabrante, à réserver uniquement en cas d'impossibilité d'embolisation.

Traumatisme splénique et CHOC HEMORRAGIQUE

CHOC HEMORRAGIQUE + Traumatisme splénique suspecté (anamnèse, épanchement à l'échographie)

Patient instable malgré remplissage vasculaire et catécholamines

LAPAROTOMIE EN URGENCE

Indications chirurgicales
- Instabilité hémodynamique
- Lésions intra abdominales associées
- Lésions extra abdominales chirurgicales urgentes
- Extravasation de PdC au TDM et absence d'accès rapide à l'embolisation

Patient stable ou stabilisé par remplissage vasculaire +/- catécholamines

TDM injectée

Saignement persistant
OU
Nécessité d'anticoagulation
OU
Nécessité d'une chirurgie en décubitus ventral (ex : rachis)
OU
Nécessité d'une chirurgie longue
OU
Présence d'une autre lésion abdominale nécessitant une chirurgie

NON

Traitement conservateur

- Patient stable ou stabilisé + Surveillance en centre spécialisé

Place pour l'embolisation splénique en radiologie interventionnelle

Indication embolisation
- Lésions à risque sur TDM (ex : fuites +)
- Grade élevé seul ? (III – IV – V)

Traumatisme hépatique et CHOC HEMORRAGIQUE

lésions de décélération, traumatisme direct

CHOC HEMORRAGIQUE + Traumatisme hépatique suspecté (anamnèse, épanchement à l'échographie)

Patient instable malgré remplissage vasculaire et catécholamines

LAPAROTOMIE EN URGENCE

Patient stable ou stabilisé par remplissage vasculaire +/- catécholamines

TDM injectée
Temps artériel – portal - veineux

Suspicion de lésion intestinale

Saignement actif sur TDM
OU
Signes cliniques de saignement
OU
Grade élevé (IV – V ?)

Embolisation artérielle en radiologie interventionnelle

Indications chirurgicales :

- Instabilité hémodynamique
- Lésions intra abdominales associées
- Lésions extra abdominales chirurgicales urgentes
- Extravasation de PdC sur TDM et absence d'accès rapide à l'embolisation

Second look systématique 24-72H

Indications radiologie interventionnelle :

- Patient stable ou stabilisé ET **Saignement actif artériel** sur TDM ou faux anévrisme
- De principe si grade élevé (IV – V)?
- En complément d'une chirurgie écourtée avec packing hépatique

Pas intérêt si saignement veineux cave et porte

Traumatisme rénal et CHOC HEMORRAGIQUE

CHOC HEMORRAGIQUE + Traumatisme rénal suspecté (anamnèse, épanchement à l'échographie)
(association fréquente avec traumatisme splénique ++)

Patient instable malgré remplissage vasculaire et catécholamines

→ LAPAROTOMIE EN URGENCE

Indication à une Néphrectomie de décompression

Patient stable ou stabilisé par remplissage vasculaire +/- catécholamines

→ TDM injectée

- Lésion rénale + Hémopéritoine OU Lésion rénale + syndrome du compartiment abdominal → Indication à une Néphrectomie de décompression → LAPAROTOMIE EN URGENCE
- Lésion de bas grade et intermédiaire I – II – III
- Lésion majeures IV - V

→ **Traitement conservateur =** Surveillance simple / Attitude non interventionnelle

Embolisation artérielle en cas d'**extravasation de PdC sur TDM** ou faux anévrisme (hémorragie retardée)

Si atteinte de l'appareil excréteur = extravasation disparait en général sans traitement

Attitude plutôt conservatrice si traumatisme du rein isolé **en absence** de fuite active avec extravasation artérielle sur TDM

Indications de dérivation urinaire (sonde urétérale ou JJ) :

- Caillottage de la voie excrétrice
- Persistance de la fuite et absence d'opacification de l'uretère d'aval

Autres traumatismes à risque hémorragique

FRACTURE FEMUR

Attelle de DONWAY en pré-hospitalier +++

→ **Traitement orthopédique** (immobilisation)

→ **CHIRURGIE**

PLAIE PERIPHERIQUE hémorragique

→ COMPRESSION

+/- GARROT (plaie artérielle)
+/-Hémostatiques locaux

CHIRURGIE

PLAIE DES GROS VAISSEAUX

→ **CHIRURGIE VASCULAIRE**

THORAX : Hémothorax :
choc hémorragique / choc obstructif possible

Secondaire à : Lésions pleurales, lacérations pulmonaires, fractures costales, artères mammaires internes, artère intercostales, gros vaisseaux, lésion de rate + brèche diaphragmatique

DRAINAGE systématique d'un hémothorax
si non drainé : risque d'infection / fibrinolyse locale

Saignement actif artère intercostale **+ patient instable** → **CHIRURGIE : hémostase + DRAINAGE**

Saignement actif artère intercostale + **patient stable ou stabilisé** → **RADIOLOGIE INTERVENTIONNELLE OU CHIRURGIE** selon avis chirurgien thoracique

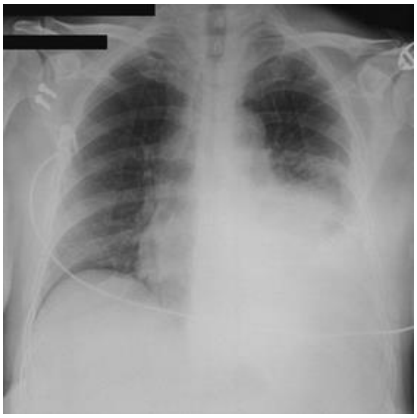

THORAX : Rupture de l'isthme aortique

(lésions de décélération) Décès sur place ++

Radio thorax : images possibles
Elargissement médiatisnal
Effacement bouton aortique
Déviation structures médianes
Hématome dôme pleural
Hémothorax gauche

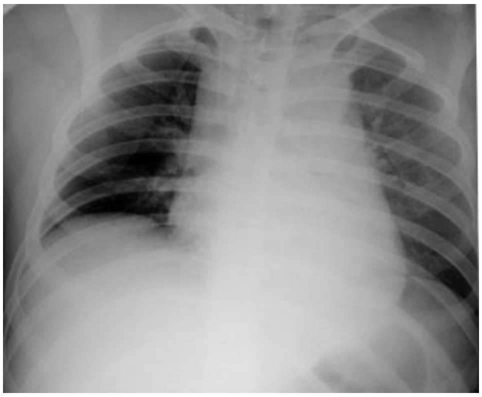

Suspicion rupture de l'isthme sur radiographie thoracique

- Patient instable malgré remplissage vasculaire et catécholamines
  - CHIRURGIE EN URGENCE
- Patient stable ou stabilisé par remplissage vasculaire +/- catécholamines
  - TDM injectée

GRADE 1
Lésion intimale
Thrombus
Hématome pariétal

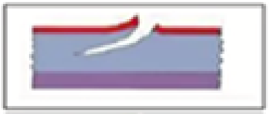

→ **Traitement médical**
Surveillance simple
Attitude non interventionnelle

GRADE 2
Flap intimal
Rupture sous adventicielle
Faux anévrisme

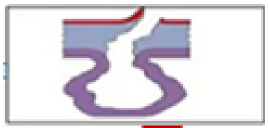

GRADE 3
Transsection aortique
Hémomédiastin
Pseudocoarctation

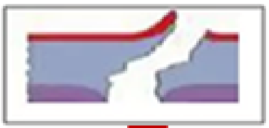

Grades 2 et 3 → ARTERIOGRAPHIE (Endo-prothèse aortique)
OU
CHIRURGIE sous CEC

Synthèse : Radiologie interventionnelle chez le polytraumatisé

Étage thoracique : **patient stabilisé**

- Endo-prothèse si rupture de l'isthme aortique
- Embolisation d'une artère intercostale

Étage abdomino-pelvien :

Hémorragies **artérielles** actives

- **HEPATIQUE**
- **SLPENIQUE**
- **RENALE**
- **AUTRE LOCALISATION**

vues au scanner chez un patient **hémodynamiquement stabilisé**

= RADIOLOGIE INTERVENTIONNELLE pour embolisation

Si instabilité persistante => CHIRURGIE EN URGENCE

Toute hémorragie pelvienne quelque soit l'état hémodynamique = RADIOLOGIE INTERVENTIONNELLE pour embolisation en absence d'épanchement péritonéal important

AVK : anti-vitamines K = anticoagulants oraux

Les AVK bloquent le cycle de régénération de la vitamine K réduite et empêchent la formation des protéines **II, VII, IX** et **X** définitives et fonctionnelles (dites « vitamino-K dépendantes) = altération coagulation = anticoagulants oraux

A noter que la synthèse des protéines C et S (anticoagulant physiologique) est également altérée.

La surveillance de leur efficacité repose sur l'INR qui est une transformation du temps de Quick (TQ) qui n'est utile que pour les patients sous AVK.

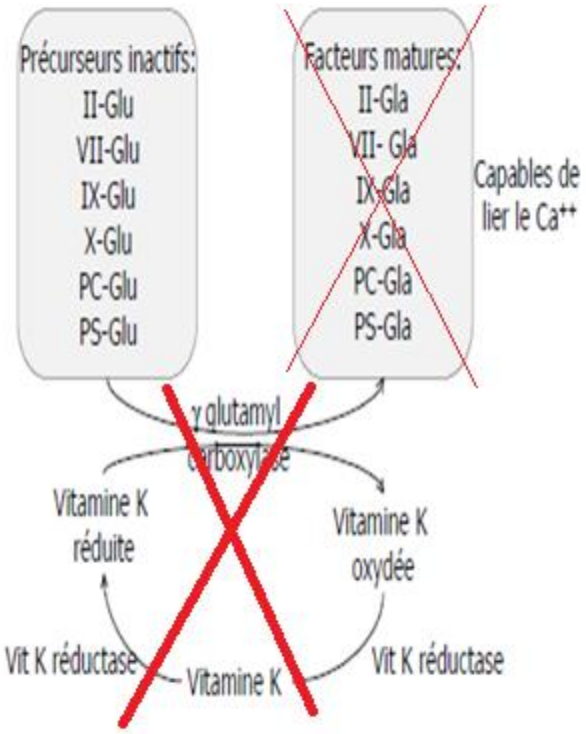

DCI	Nom commercial
WARFARINE	COUMADINE® 2 mg et 5 mg
FLUINDIONE	PREVISCAN ®<br>20 mg
ACENOCOUMAROL	MINI-SINTROM ® 1 mg<br>SINTROM ® 4 mg

Particularités de la prise en charge du choc hémorragique post-traumatique chez le patient traité par AVK

INR en urgence

Si le délai prévisible pour obtenir le résultat est important (au-delà de 30 à 60 min), la réalisation d'un INR par micro méthode au lit du patient est recommandée

Arrêt de l'AVK

+
Traitement usuel
du choc hémorragique
+
Geste hémostatique
chirurgical, endoscopique
ou endovasculaire

La mise en route du traitement **ne doit pas attendre le résultat** de l'INR s'il ne peut pas être obtenu rapidement

Concentrés de complexe prothrombinique (CCP)
25 U/kg
OU
adapté à l'INR

Vitamine K
10 mg
IVL

+

INR 30 min après administration du CCP

si INR > 1,5
Faire une **administration complémentaire de CCP**
adaptée à la valeur de l'INR (en suivant le RCP de la spécialité utilisée)
à titre d'exemple ci-dessous, RCP du CCP OCTAPLEX®

Saignements et prophylaxie péri-opératoire des accidents hémorragiques lors d'un traitement par anti-vitamines K :

La dose nécessaire dépend de l'INR déterminé avant le traitement et de l'INR cible. Le tableau suivant donne les doses approximatives (ml de produit reconstitué/kg de poids corporel) nécessaires pour normaliser l'INR (≤ 1,2 en 1 heure) en fonction de l'INR initial.

INR initial	2 - 2,5	2,5 - 3	3 - 3,5	> 3,5
Dose approximative* (ml OCTAPLEX/kg de poids corporel)	0,9 – 1,3	1,3 – 1,6	1,6 – 1,9	> 1,9

* Une dose unique ne doit pas dépasser 3000 UI (120 ml d'OCTAPLEX).

La correction de la diminution de l'hémostase induite par les anti-vitamines K dure environ 6 à 8 heures.

AOD : Anticoagulants oraux directs

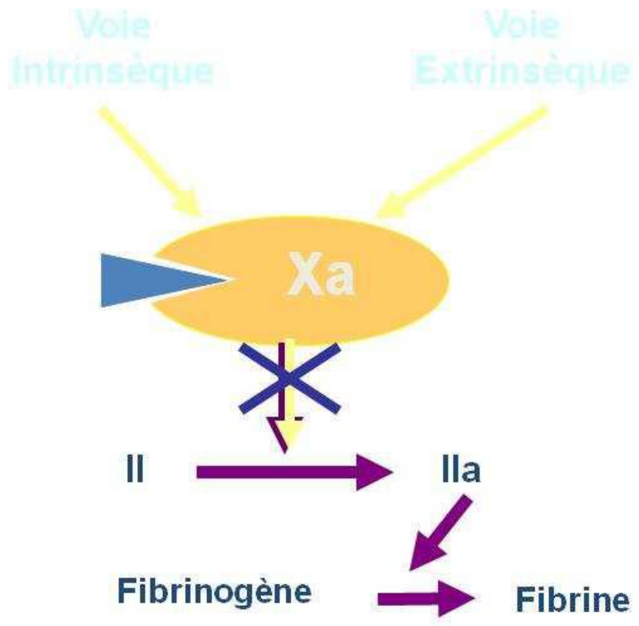

Mécanisme action du Xarelto à titre d'exemple (anti Xa)

	Dabigatran etexilate *Pradaxa®*	Rivaroxaban *Xarelto®*	Apixaban *Eliquis®*	*Edoxaban* *Lixiana®*
**Mécanisme d'action**	Direct **anti-IIa**	Direct **anti-Xa**		
**Délai d'action**	**Efficacité immédiate**			
**Biodisponibilité**	6% (prodrogue)	80-100%	80%	60%
**Elimination**	**Rénale (80%)**	Rénale, fécale et hépatique partielle	Hépatique et rénale partielle (25%)	Rénale 35% Fécale 65%
**½ vie terminale d'élimination**	12-14 h	5-9 h (jeunes) 11-13 h (âgés)	12-13 h	8-10 heures
**Surveillance biologique**	**Pas de surveillance de routine et d'adaptation biologique**			
**Antidote(s)**	**Praxbind®** (idarucizumab)	**Antidote en cours de développement :** Andexanet Alfa		
	Dialyse<br>Novoseven® ?	Feiba®<br>PPSB (Kaskadil®, Kanokad®, Octoplex®)<br>Novoseven® ?		

Prise en charge du choc hémorragique post traumatique chez le patient traité par anti-Xa (rivaroxaban et apixaban)

Neutralisation immédiate de l'effet anticoagulant de l'AOD par :

- soit des concentrés de complexe prothrombinique **activés : FEIBA 30–50 U/kg**

ou

- **CCP non activé 50 U/kg** en absence de FEIBA

éventuellement renouvelés 1 fois à 8 h d'intervalle

Mesure de l'anticoagulant dans le plasma par un test spécifique **si disponible** *(vérification imputabilité)*

+ Avis expert

Prise en charge du choc hémorragique post traumatique chez le patient traité par anti-IIa (dabigatran)

Hémorragie dans un organe critique ou choc hémorragique

↓

Idarucizumab : 5 g IV

↓

si indisponible

↓

Concentrés de complexe prothrombinique activé (**FEIBA** 30–50 U/kg) OU **CCP** non activé (50 U/kg)

Normal endothelium is thromboresistant = it gives protection against platelet activation (by production of prostacyclin PgI2), negative regulation of coagulation (glycosaminoglycans and thrombomodulin TM), and production of fibrinolytic proteins (t-PA/ PAI-1)

PRIMARY HEMOSTASIS is defined as the platelet-vessel wall interaction at the site of a vascular injury that initiates when flowing platelets recognize (though their receptors) and bind (though the adhesive protein von Willebrand) sub endothelial collagen

Vascular breach => Local vascular contraction

To reduce blood flow to the injury site

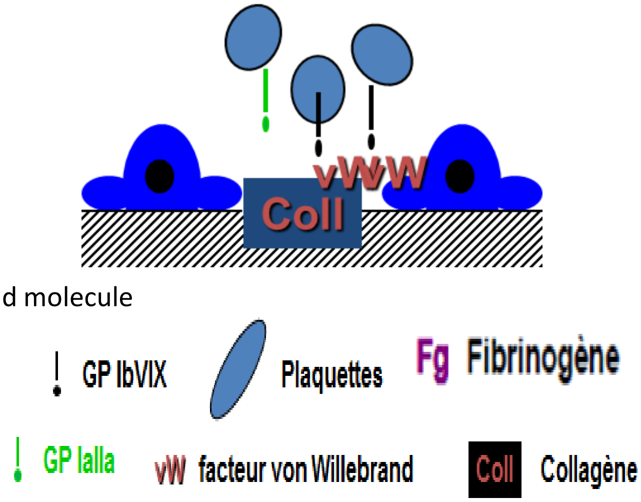

Platelets adhesion to the sub endothelium

Exposition of the von Willebrand factor
Thrombocytes can adhere to a distinct part of the von Willebrand molecule with its GP Ib/V/IX-complex and thereby accumulate at the site of damage

Platelets bind to protein von Willebrand thanks to **GPIbIX**

Platelets bind to collagen thanks to **GPIaIIa**

Platelet activation and production

When platelets come across the injured endothelium cells, they change shape, release granules and ultimately become 'sticky'

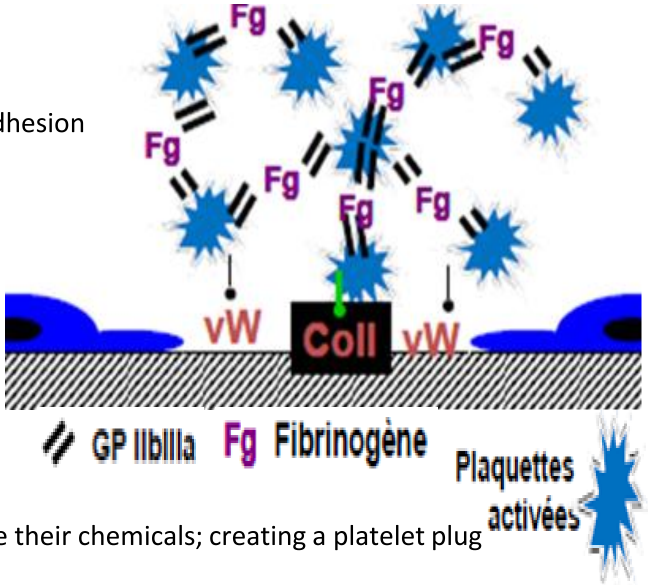

Platelet activation : they express glycoprotein receptors that interact with other platelets, producing aggregation and adhesion

Platelets release cytoplasmic granules such as ADP, serotonin , thromboxane A2.

Platelets express certain receptors, some of which are used for the adhesion of platelets to collagen.

Platelet membrane transformation = prostaglandin activation

Platelet agregation

As more chemicals are released more platelets stick and release their chemicals; creating a platelet plug and continuing the process in a positive feedback loop

They bind to each other thanks to **fibrinogen** and GP IIbIIIa with **calcium**

=> temporary blockage of a breach by a platelet plug

If the vascular breach concerns a large vessel, **COAGULATION** is necessary to solidify the clot by transforming **fibrinogen into fibrin**

COAGULATION : Intrinsic pathway / Extrinsic pathway / Common pathway

Initiating mechanism resulting from vascular injury

Platelet plug → Insoluble fibrin clot

Proteolytic reactions involving the step-wise activation of coagulation factors

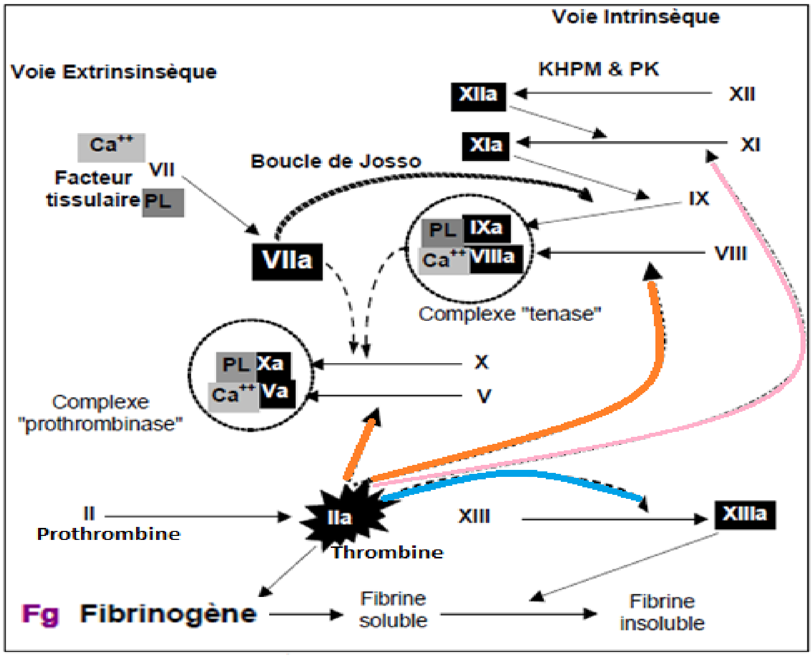

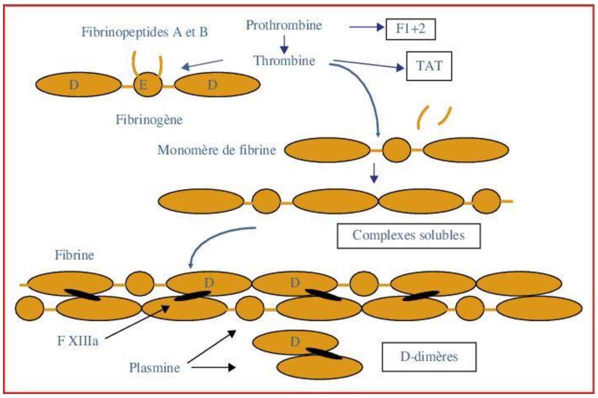

VOIE EXTRINSEQUE : *lésion endothélium démasque Facteur tissulaire*

Facteur tissulaire + VII + Ca^{2+} ⇨ VIIa

VIIa + Ca^{2+} + PL plaquettes activées + X ⇨ Xa

VOIE INTRINSEQUE : *collagène du sous-endothélium à nu entre en contact avec facteurs XII et XI (dits "facteurs contacts")*

XII + KHPM + kallicréine ⇨ XIIa

XIIa active XI ⇨ XIa

XIa active IX ⇨ IXa

IXa + VIII + Ca^{2+} + PL plaquettes activées + X ⇨ Xa

TRONC COMMUN : *au niveau des phospholipides des plaquettes activées en présence de Ca++*

Xa + V + II (prothrombine) ⇨ IIa (thrombine)

IIa protéolyse **Fg Fibrinogène** en monomères de fibrine

ces monomères s'associent en polymères ⇨ fibrine soluble

IIa + XIII + Ca^{2+} ⇨ XIIIa

XIIIa va stabiliser la fibrine soluble en réseau insoluble

Premières traces IIa (thrombine) activent V et VIII ⇨ Va et VIIIa ⇨ X 10 000 génération de thrombine

caillot formé reste en place 10-14j le temps de la cicatrisation puis fibrinolyse…

COAGULATION : Intrinsic pathway / Extrinsic pathway / Common pathway

Initiating mechanism resulting from vascular injury

Platelet plug ⇒ Insoluble fibrin clot

Proteolytic reactions involving the step-wise activation of coagulation factors

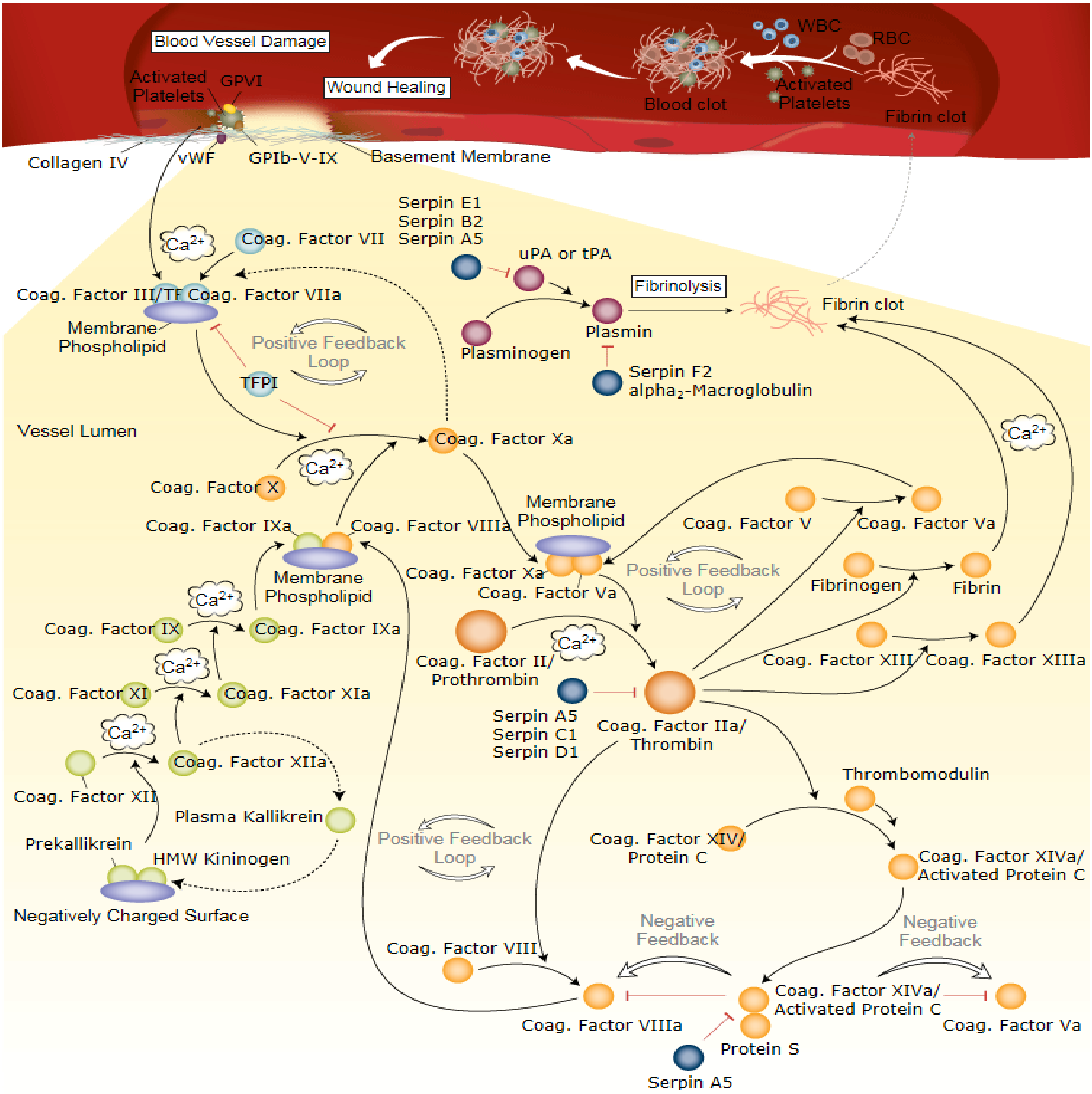

https://www.rndsystems.com/pathways/blood-coagulation-signaling-pathways

COAGULATION INHIBITORS

How is the insoluble clot limited to the vascular breach

Thrombin on fibrin clot = **procoagulant** + Thrombine self-amplification

Excess thrombin out of the firbin clot = **anticoagulant** by self-inhibition (AT and PC/PS system).

Antithrombin (AT) = main coagulation inhibitor

Excess thrombin from fibrin clot will be neutralized by AT

Formation of **stable** [**Antithrombin-Thrombin] complex = irreversible inhibition.**

Moreover : AT can also neutralize the majority of other clotting factors: Xa, IXa, XIa, XIIa, kallikréine. It doesn't neutralize VIIa.

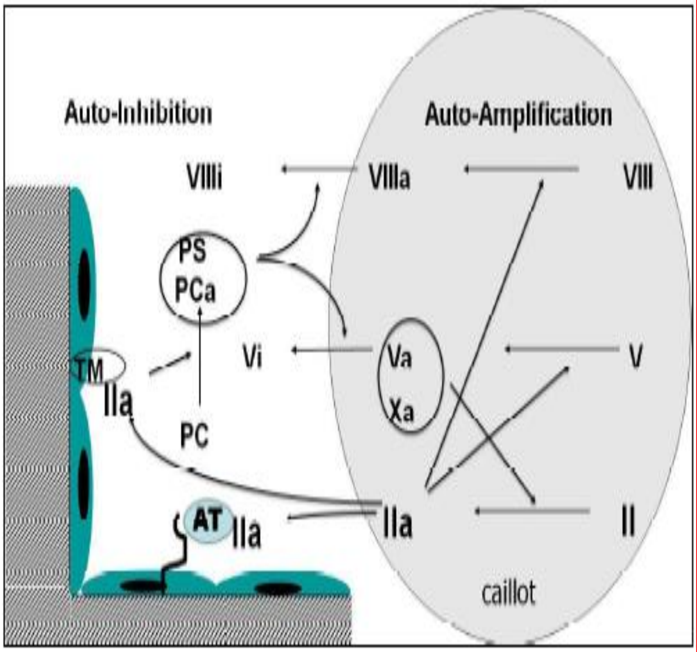

Protein C and S system

Excess thrombin from fibrin clot binds to a specific receptor on endothelium: Thrombomodulin (TM).

After binding to TM, thrombine loses its procoagulant properties and will be able to **activate protein C.**

In the presence of phospholipids, calcium and its cofactor protein S (PS), **activated protein C** binds to the clot and **degrades Va and VIIIa factors**, **decelerating coagulation**.

Extrinsic pathway inhibitor system:
Tissue factor pathway inhibitor (TFPI)

1/ TFPI binds to factor Xa
2/ then binds to [tissue factor – VIIa] complex
=> quaternary complex
Therefore, TFPI **rapidly inhibits the activity of the [tissue factor / VIIa] complex.**

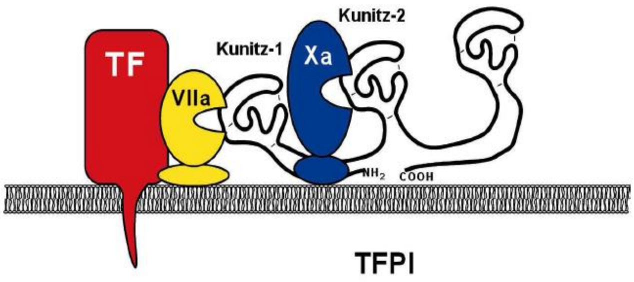

FIBRINOLYSIS = Degradation of intravascular fibrin clot by Plasmin

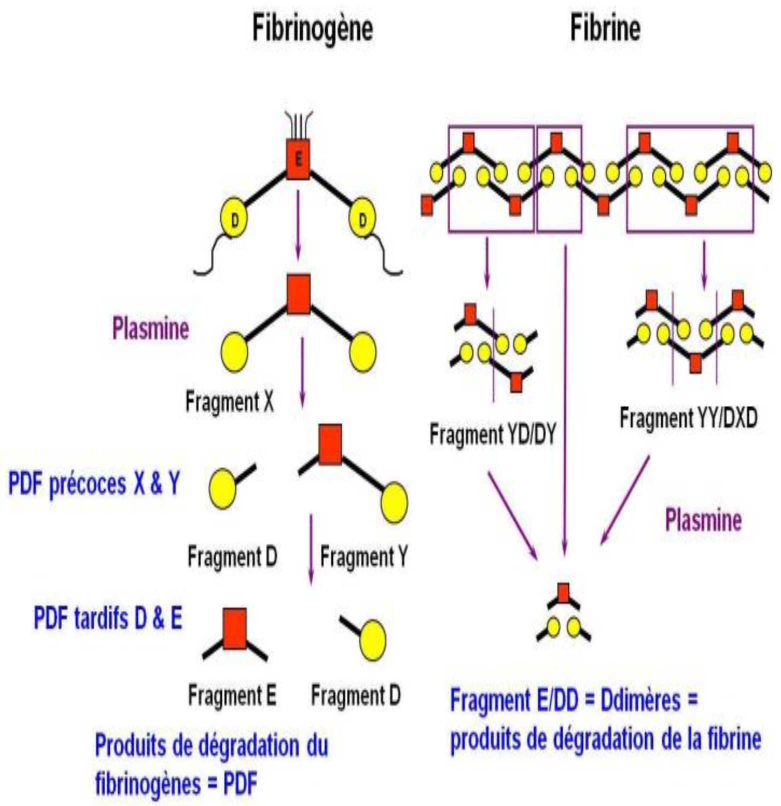

Fibrin degradation by plasmin

Plasmin cuts firbin filaments **into so-called fibrin degradation products**, including Ddimers

Degradation of fibrinogen by plasmin

Plasmin is also capable of degrading fibrinogen, producing **fibrin degradation products (FDP)**

BUT there is no circulationg plasmin in the physiological state. Plasmin results from the activation of plasminogen (inactive).

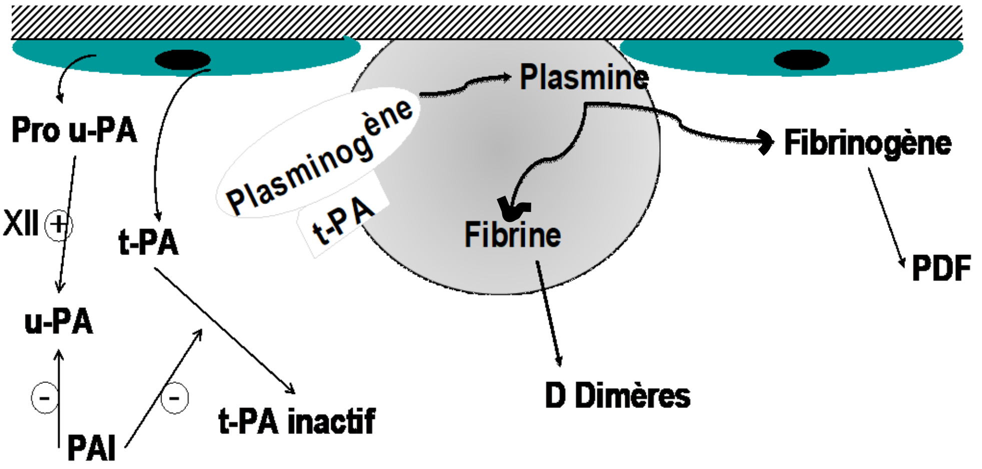

Two main plasminogen activators:

t-PA = tissue plaminogen activator

- Synthesized by the endothelial cell
- Not effective in circulation because it is complexed with its inhibitor PAI-1

On the other hand, since it has a very high affinity for fibrin, it also binds fibrin of the clot. Thus it escapes its inhibitor and, within the clot, it can transform plasminogen into active plasmin.

pro-urokinase (Pro u-PA) is a second plasminogen activation system

Transfusion : What to prescribe urgently in case of hemorhagic shock?

TOTAL BLOOD VOLUME : female = 60-65 mL/kg et male 70-75 mL/kg
1g/dL = 3% hematocrit // 1 PRBC = about 250 mL with 60% hematocrit
To say it simply, 1 PRBC transfusion increases hemoglobin by 1 g/dL

In the absence of immunohematological data :

PRBC TRANSFUSION O RH:1 KEL:-1 => O positive

Except for women from birth to teh end of the reproductive period (= PRBC O negative)

If massive transfusion, availability of PRBC takes precedence over compatibility of blood groups except ABO system

Hemoglobin target between **7 et 9 g/dL**
Particularities : **Coronary, BBlockers, Traumatic Brain Injury : 9-10 g/dL**

Transfuse FFP in association with PRBC with a **ratio of FFP:PRBC between 1/2 et 1/1**

FRESH FROZEN PLASMA

Start **plasma transfusion quickly**, ideally **at the same time as the PRBC BEFORE** biological results

In case of unavailability of ABO isogroup plasma:
AB plasma can be used regardless of the patient's group

Early Pooled Platelets (PP) transfusion, usually during **second transfusion prescription**

Target : Platelets counts **above 50 G/L**

↓ Persistent bleeding

↓ If associated traumatic brain injury

↓

Target: Platelets counts **above 100 G/L**

Should ionized calcium be monitored in hemorrhagic shock?

MASSIVE TRANSFUSION

Ionized hypocalcemia ionisée can occur during a massive transfusion due to citrate used as an anticoagulant in labile blood products (especially FFP). The use of colloids can also play a role.

Existence of low liver blood flow during shock prevents citrate metabolism

Monitoring of **ionized calcium** is recommended in case of massive transfusion.

Ionized calcium concentration must be maintained > 0,9 mmol/L by a calcium chloride supply circulating on a separate venous catheter

BECAUSE

Decrease in ionized calcium, which is biologically active, exposes to increased **cardiovascular failure and even circulatory arrest**.
It could also worsen **hemostatic failure.**

EXACYL® : Tranexamic acid nd hemorrhagic shock

ANTI- fibrinolytic drug Synthetic lysin analog = **Decrease in the transformation of plasminogen to plasmin**

Short half-life: 80–120 min after IV injection

No increased risk of venous or arterial thrombosis

CRASH2

As soon as possible at **1g IV 10 min bolus**

Followed by 1g infused over 8h

In traumatic hemorrhagic shock patients.

Tranexamic acid <u>should not be initiated beyond the 3rd hour</u> following trauma

(Increased mortality if administered after 3rd hour)

Ref : [Effects of tranexamic acid on death, vascular occlusive events, and blood transfusion in trauma patients with significant haemorrhage (CRASH-2): a randomised, placebo-controlled trial – Lancet - 2010]
Réf : [The importance of early treatment with tranexamic acid in bleeding trauma patients: an exploratory analysis of the CRASH-2 randomised controlled trial – Lancet - 2011]

**<u>Fibrinogen</u>** CLOTTAFACT®, LFB; RIASTAP®, CSL Behring<br>Substrate of prothrombin (IIa) and transformed into fibrin for stabilization of the fibrin clot<br>3 g for a 70 kg adult<br>No bolus<br>IV over 10-20 min<br>on peripheric venous acces (risk of fibrinogen accumulation in LA if administred on Central venous catheter)	Recommended for plasma fibrinogen concentration < or = 1.5 g/L, or thromboelastographic (-metric) parameters concluding for functional fibrinogen deficiency.	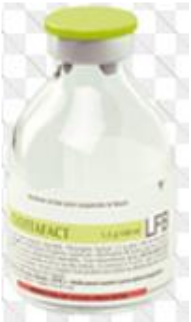
**<u>PCC : prothrombin complex concentrates</u>**<br>KANOKAD® LFB; OCTAPLEX® Octapharma ; CONFIDEX®, CSL Behring<br><u>50 U/kg</u><br>- **II**<br>- **VII**<br>- **IX**<br>- **X**	Only in hemorrhagic shock for **patients who take vitamin K antagonists (VKA)**<br><br>Some European teams use non-activated PCC as a substitute for FFP transfusion in hemorrhagic shock without the condition of VKA treatment. These practices are not currently recommended in France	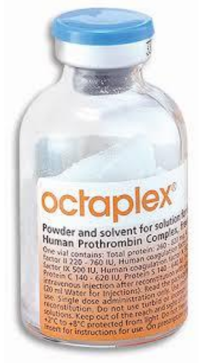
**<u>APCC : activated prothrombin complex concentrate</u>** Factor Eight Inhibitor Bypassing Activity ou FEIBA®, Baxter<br><u>30–50 U/kg</u><br>- **II**<br>- **VII**<br>- **IX**<br>- **X**	Only in hemorrhagic shock for **patients who take direct thrombin inhibitors** (dabigatran, rivaroxaban, apixaban)<br><br>= immediate neutralization of the anticoagulant effect	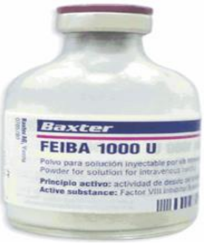
**<u>VII Factor activated</u>**<br>NOVOSEVEN®, Novo Nordisk<br><br>The recommended initial dose is 80 to 200 µg/kg.	In case of hemorrhagic shock, FVIIa should NOT be used as a first-line treatment.<br>It should only be considered if the bleeding cannot be controlled despite:<br>Mechanical hemostatic procedures AND Use of tranexamic acid AND Transfusion of labile blood products AND The transfusion of fibrinogen concentrates AND the correction of severe hypothermia AND the correction of severe acidosis	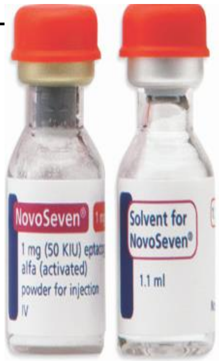

The Polytraumatized patient
Injury assessment and Orientation

Patient who suffered from violent trauma regardless of the apparent lesions: 1 Vittel criterion = Serious Polytrauma

Examen initial du patient	Glasgow < 13<br>SaO_2 < 90%<br>Pression artérielle systolique < 90 mmHg
Circonstances de l'accident	Victime éjectée – projetée - écrasée<br>Décès dans l'accident<br>Chute > 6m – explosions - blast
Prise en charge préhospitalière	Ventilation assistée<br>Remplissage > 1000 mL<br>Catécholamines
Lésions observées ou suspectées	Trauma pénétrant – volet thoracique – trauma bassin<br>Amputation de membre – ischémie aiguë de membre<br>Brûlure – suspicion de lésion médullaire
Caractéristiques du patient	Age > 65 ans<br>Grossesse au 2ème et 3ème trimestre<br>Tares associées

Monitoring
2 peripheric venous access
Blood test
FAST
Chest radiography
Pelvic radiography
Fight against hypothermia

Polytraumatized patient

- Patient hemodinamically unstable → Surgery / Embolization ↔ Body CT scan
- Fluid loading and patient stabilized → Body CT scan
- Patient hemodynamically stable → Body CT scan

PRIORITY in the management of failures:
1/ HEMORRHAGE 2/ TBI 3/ PERIPHERIC INJURY

Objectives of BP, Fluid loading, Catecholamines

"Tachycardia from a loss of 20-25% of the total blood mass and Hypotension from a loss of 40% of the blood mass"

Until the bleeding is uncontrolled

Limit the fluid loading to maintain the BP objectives

Without Trauma Brain injury (TBI)

Tolerate hypotension

Objective **SBP 80–90 mmHg**

or

MAP 60– 65 mmHg

If Trauma Brain Injury (Coma Glasgow Score < or = 8)

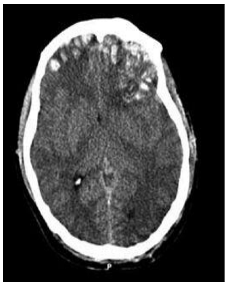

MAP > 80 mmHg, before having the brain monitoring despite the risk of hemorrhagic degradation

First intention

Saline solution

Do not use albumin

No hypotonic fluid solution

Intraosseous infusion > CVC if PVC unavailable

If peripheral venous catheter (PVC) is quickly available

Central venous catheter (CVC) doesn't delay the hemodynamic stabilization and etiological treatment

HES if saline solutions **saline solutions only is non sufficient** for maintening volemia.

Lowest dose

As short a time as possible

To assess the degree of hypoperfusion and tissue hypoxia:

[arterial lactate]

Administer a vasoconstriction therapy if low BP persists (SPB < 80 mmHg) **after initiation of fluid loading**

Norepinephrine in first intention

The administration of Norepinephrine must occur on a central venous catheter (CVC). But ***in the emergency context*** *, and waiting for a central venous catheter*

You must use a peripheric venous catheter

Pelvic trauma and HEMORRHAGIC SHOCK

HEMORRHAGIC SHOCK + Pelvic trauma + hemoperitoneum(FAST)

- Patient hemodynamically unstable despite fluid loading and catecholamines
  - Massive hemoperitoneum → Laparotomy → Arterial embolization
  - Moderate or minimal hemoperitoneum → Arterial embolization
- Patient hemodynamically stable with fluid loading or catecholamines → CT scan → (Arterial embolization)

HEMORRHAGIC SHOCK + Pelvic trauma + without hemoperitoneum (FAST)

- Patient hemodynamically unstable despite fluid loading and catecholamines
- Patient hemodynamically stable with fluid loading or catecholamines → CT scan showing an **active bleeding in pelvis**

→ Arteriography +/- Embolization in Interventional Radiology = WHATEVER THE HEMODYNAMIC STATE IS

Difficult haemostasis surgery has to be reserved only if embolization is not possible.

Splenic trauma and HEMMORHAGIC SHOCK

HEMMORHAGIC SHOCK + Suspicion of splenic injury (anamnesis, effusion on ultrasound)

Unstable patient despite vascular filling and catecholamines

EMERGENCY LAPAROTOMY

Surgical indications
- Hemodynamic instability
- Associated intra-abdominal injuries
- Emergency extra-abdominal surgical injuries
- Contrast extravasation and no quick access to embolization

Patient stable or stabilized by vascular filling +/- catecholamines

Contrast CT

Persistant bleeding
OR
Need for anticoagulation
OR
Need of ventral decubitus position during surgery (e.g. spine)
OR
Need for long surgery
OR
Presence of another abdominal injury requiring surgery

NO

Conservative treatment

- Stable or stabilized patient +
Surveillance in a specialized centre

Splenic embolization in interventionnal radiology

Embolization indications:
- injury on CT scan (e.g. extravasation)
- Only high grade? (III – IV – V)

Hepatic trauma and HEMMORHAGIC SHOCK

Deceleration injury, direct trauma

HEMORRHAGIC SHOCK+ Suspicion of hepatic trauma (anamnesis, effusion on ultrasound)

Unstable patient despite vascular filling and catecholamines

EMERGENCY LAPAROTOMY

Patient stable or stabilized by vascular filling +/- catecholamines

Contrast CT
Arterial – portal – veinous phases

Suspicion of intestinal injury

Contrast extravasation
OR
Clinical signs of bleeding
OU
High grade (IV – V ?)

Arterial embolization in interventionnal radiology

Surgical indications
- Hemodynamic instability
- Associated intra-abdominal injuries
- Emergency extra-abdominal surgical injuries
- Contrast extravasation and no quick access to embolization

Systematic second-look surgery after 24-72H

Arterial embolization indications:
- Stable or stabilized patient AND **arterial active bleeding** on CT or ou pseudoaneurysm
- High grade (IV – V)?
- In addition to abbreviated surgery with hepatic packing

No benefit if cava or portal bleeding

Renal trauma and HEMORRAGHIC SHOCK

HEMORRHAGIC SHOCK+ Suspicion of renal trauma (anamnesis, effusion on ultrasound)
(frequent association with splenic trauma)

Unstable patient despite vascular filling and catecholamines
→ EMERGENCY LAPAROTOMY

Patient stable or stabilized by vascular filling +/- catecholamines
→ Contrast CT

- Renal injury + Hemoperitoneum OR Renal lesion + abdominal compartment syndrome → Indication of decompression surgery → EMERGENCY LAPAROTOMY
- Low grade and intermediate lesion I – II – III → Conservative treatment
- High grade IV - V → Conservative treatment

Conservative treatment= Simple monitoring

Arterial embolization in case of **contrast extravasation on CT** or pseudoaneurysm (delayed bleeding)

If excretory system is affected= extravasation usualy disappears without treatment

Conservative approach if isolated renal trauma **without** contrast arterial extravasation artérielle on CT

Urinary internal diversion indications (ureteral stent or JJ catheter) :

- Obstructing blood clot in the ureter
- Renal contrast extravasation with no ureteral opacification

Other traumas with hemorrhagic risk

FEMORAL FRACTURE

Prehospital DONWAY splint +++

→ **Non surgical orthopedic treatment** (immobilisation)

→ **SURGERY**

PERIPHERAL bleeding WOUND

→ COMPRESSION

+/- TOURNIQUET (arterial wound)
+/- Local hemostatics

SURGERY

LARGE VESSEL WOUND → **VASCULAR SURGERY**

THORAX : Hemothorax :

Hemorrhagic shock/ possible obstructive shock

Secondary to: pleural injury, pulmonary laceration, costal fracture, internal mammary arteries, intercostal arteries, large vessels, splenic trauma + diaphragmatic breach

Systematically DRAIN hemothorax
otherwise : infection risk / local fibrinolysis

Active bleeding of intercostal artery **+ instable patient** → **SURGERY : hemostasis + DRAINAGE**

Active bleeding od intercostal artery + **stable or stabilized patien** → **NTERVENTIONNAL RADIOLOGY OR SURGERY** according to thoracic surgeon's decision

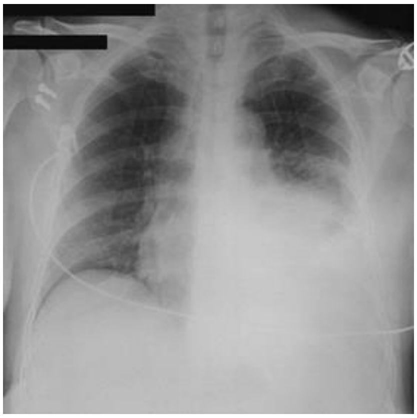

THORAX : Rupture of aortic isthmus

(deceleration injury) Death on site ++

Chest radiography:
Mediastinal widening
Disappearance of aortic knob
Midline shift
Extrapleural hematoma
Left hemothorax

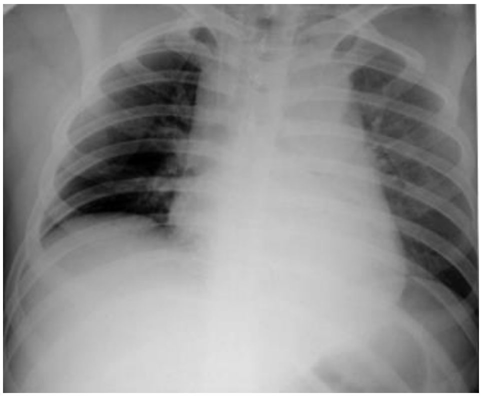

Suspicion rupture of aortic isthmus on chest radiography

Unstable patient despite vascular filling and catecholamines

EMERGENCY SURGERY

Patient stable or stabilized by vascular filling +/- catecholamines

Contrast CT

GRADE 1
Intimale breach
Thrombus
Parietal hematoma

GRADE 2
Intimal flap
Sub-adventitious dissection
Pseudo-aneurysm

GRADE 3
Aortic transection
Hemomediastinum
Pseudocoarctation

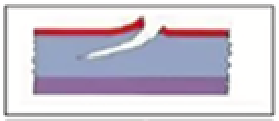

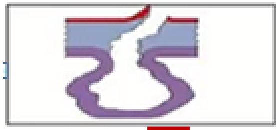

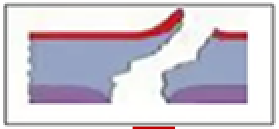

Non surgical treatment
Simple monitoring

ARTERIOGRAPHY (aortic endo-prosthesis)
OR
SURGERY under ECC

Summary : Interventionnal Radiology in trauma patients

Thorax : **stabilized patient**

- Endo-prosthetic if rupture of aortic isthmus
- Intercostal artery embolization

Abdomen and pelvis :

Active **arterial** bleeding

- **HEPATIC**
- **SPLENIC**
- **RENAL**
- **OTHER LOCALISATION**

On CT scan in a **hemodynamcally stabilized** patient
= INTERVENTIONNAL RADIOLOGY for embolization

If persistent instability => EMERGENCY SURGERY

Any pelvic bleeding whatever hemodynamic state = **INTERVENTIONNAL RADIOLOGY for embolization in the absence of significant peritoneal effusion**

Vitamin K antagonists VKA

VKA block the regeneration cycle of reduced vitamin K and prevent the formation of definitive and functional coagulation factors (II, VII, IX and X).
In result, there is an alteration of the coagulation pathway.

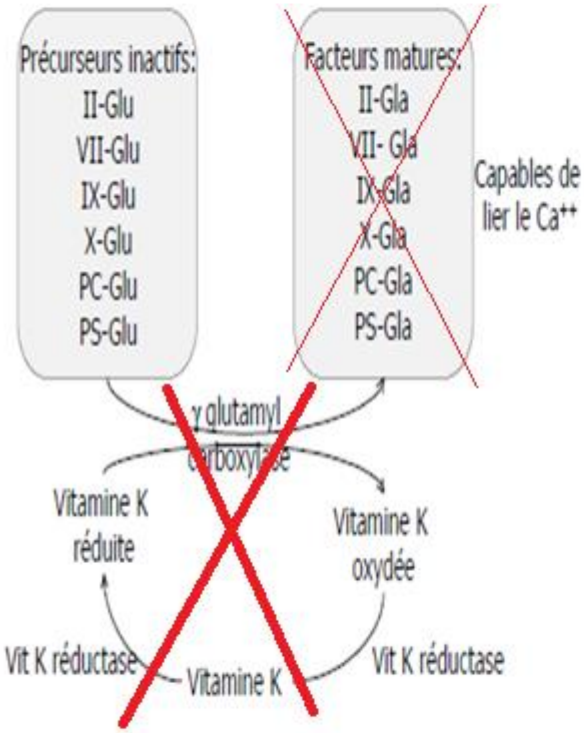

It should be noted that the synthesis of proteins C and S (physiological anticoagulant) is also altered with VKA.

The monitoring of the effectiveness of the VKA is based on INR, which is a transformation of the prothrombin time (PT).
It is only useful for patients under a VKA therapy.

DCI	Nom commercial
WARFARINE	COUMADINE® 2 mg et 5 mg
FLUINDIONE	PREVISCAN ®<br>20 mg
ACENOCOUMAROL	MINI-SINTROM ® 1 mg<br>SINTROM ® 4 mg

Hemorragic shock post trauma in patients under VKA therapy

INR

If the expected time to obtain the result is long (more than 30 to 60 minutes), the use of an INR by micro-method at the patient's bedside is recommended

Stop VKA

\+
Usual treatment of hemorrhagic shock
\+
Hemostasis surgery or interventional radiology

The start of the treatment should not wait for the result of the INR if it cannot be obtained quickly

Prothrombin complex concentrates (PCC)
25 U/kg
OR
Depending on INR

Vitamin K
10 mg
IV

\+

INR 30 min after PCC

If INR > 1,5
Make an **other PCC administration**
adapted to the INR (following the recommendations of the treatment used)

Saignements et prophylaxie péri-opératoire des accidents hémorragiques lors d'un traitement par anti-vitamines K :

La dose nécessaire dépend de l'INR déterminé avant le traitement et de l'INR cible. Le tableau suivant donne les doses approximatives (ml de produit reconstitué/kg de poids corporel) nécessaires pour normaliser l'INR (≤ 1,2 en 1 heure) en fonction de l'INR initial.

INR initial	2 - 2,5	2,5 - 3	3 - 3,5	> 3,5
Dose approximative* (ml OCTAPLEX/kg de poids corporel)	0,9 – 1,3	1,3 – 1,6	1,6 – 1,9	> 1,9

* Une dose unique ne doit pas dépasser 3000 UI (120 ml d'OCTAPLEX).

La correction de la diminution de l'hémostase induite par les anti-vitamines K dure environ 6 à 8 heures.

Direct thrombin inhibitors DTI

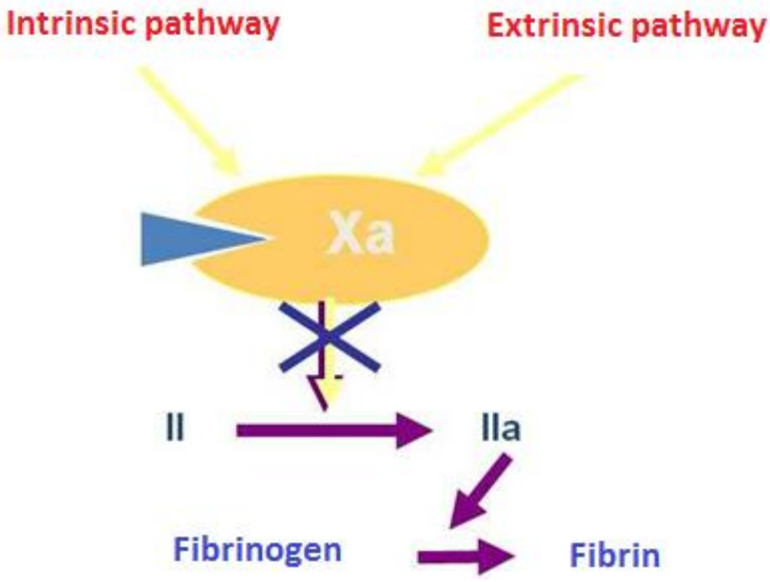

	Dabigatran etexilate *Pradaxa®*	Rivaroxaban *Xarelto®*	Apixaban *Eliquis®*	*Edoxaban* *Lixiana®*
mechanism of action	Direct anti-IIa	Direct anti-Xa		
action delay	immediate effect			
Biodisponibility	6%	80-100%	80%	60%
Elimination	Renal elimination	Partial hepatic, renal and faecal	Partial hepatic and renal (25%)	Renal 35% Faecal 65%
Half life elimination	12-14 h	5-9 h 11-13 h	12-13 h	8-10 heures
Routine plasma monitoring	NONE			
Antidote(s)	Praxbind® (idarucizumab)	Developping : Andexanet Alfa		
	RRT Novoseven® ?	Feiba® PPSB (Kaskadil®, Kanokad®, Octoplex®) Novoseven® ?		

Projet WhatsAR – RAMEN J. – CLAVIER T. – COMPERE V.

Management of post-traumatic hemorrhagic shock in patients treated with Direct thrombin inhibitors DTI (rivaroxaban and apixaban)

Immediate neutralization of the anticoagulant effect of DTI by :
Activated prothrombin complex concentrate
FEIBA 30–50 U/kg
Or
Prothrombin complex concentrate :
PCC 50 U/kg
possibly renewed once every 8 hours

Specific test for the measure of plasmatic concentration of DTI (if available)

Expert opinion

Management of post-traumatic hemorrhagic shock in patients treated with Direct thrombin inhibitors DTI (dabigatran)

Hemorrhage in a critical organ or hemorrhagic shock

↓

Idarucizumab : 5 g IV

↓

unavailable

↓

Activated prothrombin complex concentrate **FEIBA 30–50 U/kg**
Or
Prothrombin complex concentrate : **PCC 50 U/kg**
