# Supplementary material for: Use of the Smartphone App WhatsApp as an E-Learning Method for Medical Residents: Multicenter Controlled Randomized Trial
Source: JMIR Mhealth Uhealth. 2019 Apr 9;7(4):e12825. doi: 10.2196/12825 (PMC6477573; doi:10.2196/12825)
Supplement: Multimedia Appendix 2 [file mhealth_v7i4e12825_app2.pdf]

Cas clinique Semaine 1: <u>BASES EN HEMOSTASE ET REANIMATION DU CHOC HEMORRAGIQUE</u>

Patient de 53 ans, chute d'un échafaudage de 4m. Bilan lésionnel : déformation du membre inférieur gauche (cuisse augmentée de volume). Pas d'autre lésion visible. Il prend du KARDEGIC et du PLAVIX "pour le cœur". Constantes: FC 138/min- PA 87/43 mmHg - Glasgow 15/15 - SpO2 100% sous 6l/min MHC. Vous suspectez un choc hémorragique secondaire à une fracture fermée du fémur gauche.

1 - Chez ce patient en état de choc hémorragique, quels seront vos objectifs tensionnels ?

PAS 80–90 mmHg OU PAM entre 60– 65 mmHg **chez le patient NON traumatisé crânien**

TOLERER hypotension artérielle pour minimiser les risques d'aggravation du saignement tant que l'hémostase chirurgicale et/ou radio-interventionnelle n'est pas réalisée.

A noter : Pour les patients traumatisés crâniens graves (Coma Glasgow Score 8) en choc hémorragique, PAM > 80 mmHg, avant de disposer d'un monitorage cérébral, en dépit du risque d'aggravation du saignement

2 - Quel traitement devez-vous instaurer dans les 3h suivant le traumatisme (+posologie)? Quel est son mécanisme d'action?

Acide tranexamique (EXACYL®) : 1 g IVL sur 10 minutes puis relai 1g IVSE sur 8h.

Mécanisme action : molécule anti-fibrinolytique. Analogue synthétique de la lysine entrainant une diminution de la transformation de plasminogène en plasmine => inhibition de la fibrinolyse.

3 - Il y a-t-il une contre-indication à ce médicament chez ce patient possiblement coronarien ?

NON, Aucune contre-indication dans ce contexte. Bénéfice chez le patient coronarien.

Reference: Effects of tranexamic acid on death, vascular occlusive events, and blood transfusion in trauma patients with significant haemorrhage (CRASH-2): a randomised, placebo-controlled trial – Lancet – 2010. L'acide tranexamique réduit significativement la mortalité toutes causes confondues (RR = 0,91 ; IC 95 % = 0,85–0,97), particulièrement la mortalité par hémorragie (RR = 0,85 ; IC 95 = 0,76– 0,96). Le taux d'évènements thrombotiques n'est pas augmenté par ce traitement, celui d'infarctus du myocarde est même réduit.

Après remplissage (1000 ml de cristalloïdes) il est stabilisé sur le plan hémodynamique. A l'Hemocue, Hb = 7g/dL. Vous apprenez que ce patient est porteur d'un stent actif depuis 6 mois.

4 - Quel va être votre objectif transfusionnel chez ce patient en particulier

Objectif hémoglobine entre 9-10 g/dL.

5 - Quelle sera votre 1ere prescription pour l'EFS (type et quantité de produits sanguins) ?

À titre d'exemple :

-2 à 3 concentrés de culots globulaires CGR.

-2 à 3 plasma frais congelés PFC.

-1 mélange de concentrés plaquettaires MCP vitale devant la dysfonction plaquettaire induite par la prise de Kardegic et de Plavix au long cours et le contexte de choc hémorragique.

Le bilan biologique revient : Hémoglobine 7.6 g/dL, Plaquettes 280 G/L, TP 80%, TCA 1.12, Fibrinogénémie à 0.9 g/L

6 - Quel traitement complémentaire instaurez-vous (+posologie) ? Avec quel objectif ?

Fibrinogène 3 g (posologie pour un adulte de 70 kg), pas de bolus, IVL sur 10-20 min , sur VVP (risque accumulation fibrinogène dans OG si VVC), Objectif Fibrinogénémie > 1.5-2 g/L.

La transfusion de PFC va contribuer également à corriger l'hypofibrinogénémie mais de façon LIMITEE. Les plasmas apportent l'ensemble des protéines plasmatiques en particulier les facteurs de la coagulation et les fractions du complément. La préservation de l'activité des facteurs de coagulation est contrôlée par la mesure de la concentration de facteur VIII et du fibrinogène. La norme française pour le facteur VIII exige qu'elle soit d'au moins 0,7 UI/mL pour le PFC-Se et d'au moins 0,5 UI/mL pour le PFC-IA, le PFC-SD et le plasma lyophilisé. Les plasmas sont les seuls produits capables d'apporter du facteur V, de la protéine S, du plasminogène et de la métalloprotéase clivant le facteur Von Willebrand (ADAMTS 13), ainsi que certaines fractions du complément. Ces facteurs ne sont pas disponibles sous forme purifiée stable parmi les médicaments dérivés du sang.

7- Quel est le mécanisme de l'hypofibrinogenemie ?

Avec les plaquettes, le fibrinogène, précurseur de la fibrine, est un élément essentiel de la constitution d'un thrombus hémostatique solide. Un abaissement de la fibrinogénémie est fréquent dans les chocs hémorragiques.

Le déficit en fibrinogène est particulièrement marqué en cas de **dilution**, et peut avoir aussi d'autres causes, notamment **une fibrinolyse** s'intégrant dans le cadre de la coagulopathie post traumatique :

Transformation du plasminogène en plasmine suite à la libération de l'activateur tissulaire du plasminogène (t-PA) + Inhibition de l'inhibiteur de l'activateur du plasminogène (PAI - 1) = hyperfibrinolyse

Résultat = dégradation de la fibrine par la plasmine ; consommation du fibrinogène = hypofibrinogénémie

Cas clinique Semaine 2 : PRISE EN CHARGE DU CHOC HEMORRAGIQUE POST TRAUMATIQUE

Patient de 20 ans victime d'un AVP moto/VL. A l'examen : PA = 90/50 (PAM 63) mmHg, FC 120/min, Glasgow 11/15, SpO2 100% sous MHC 6/min. EVA 8/10, T° = 35,2°C, pupilles isocores symétriques, déformation du pelvis avec suspicion de fracture du bassin, ecchymose frontale avec notion de traumatisme crânien (perte de connaissance de 2 min).

1-Quelles sont les thérapeutiques devant être débutées en pré-hospitalier ? Quel est l'objectif de pression artérielle moyenne de ce patient ?

Immobilisation de l'axe tète cou tronc

Ceinture de contention pelvienne

2VVP de bon calibre.

Lorsque VVP bon calibre impossible et pré-hospitalier : accès intra- osseux >> KTC

Remplissage vasculaire avec des solutés cristalloïdes en première intention : 1000-1500 ml en débit libre

Analgésie (paracétamol, morphine, kétamine)

Réchauffement : lutte contre hypothermie

Acide tranexamique IVL 1g sur 10 min

Objectif de PAM > 80 mmHg (traumatisé crânien grave avec troubles de la vigilance)

Après 1500 mL de cristalloïdes et mise en place de NORADRENALINE (obj PAM > 80 mmHg) par le SMUR, les constantes sont les suivantes : PA 112/63 (PAM 79) mmHg, FC 114 bpm, Glasgow 13/15, EVA 2/10, SpO2 100% sous 6L/min MHC.

2-Quels examens complémentaires à visée diagnostique réalisez-vous au déchoquage ?

En première intention : **FAST-échographie** (recherche d'un épanchement liquidien intra-abdominal, d'un épanchement gazeux ou liquidien thoracique, évaluation du péricarde et du myocarde, recherche de signes d'HTIC au doppler trans-crânien) + **Radiographie thoracique** (recherche d'un épanchement gazeux ou liquidien thoracique, d'un élargissement du médiastin (rupture de l'isthme ?), de fractures costales) + **Radiographie de bassin** (recherche d'un fracture +/- instable du bassin)

En seconde intention : Au vu de la stabilisation du patient qui présente cliniquement au moins deux atteintes avec risque vital (bassin et traumatisme crânien), il est raisonnable d'organiser rapidement un body-TDM pour le bilan lésionnel. Si point d'appel périphérique (fracture osseuse) envisager des radiographies osseuses ciblées.

3-Réalisez-vous un nouveau remplissage ? L'infirmière vous demande quel soluté de perfusion vous souhaitez mettre en garde veine avant le scanner, que choisissez-vous ? Vous avez à votre disposition : du sérum glucosé G5%, du NaCl 0.9%, du Ringer lactate, des solutés à base d'hydroxyéthylamidons (Voluven ®), des solutés à base d'albumine 20%.

Solutés cristalloïdes non hypotoniques (NaCl 0.9% ou Isofundine) et tant que le saignement n'est pas contrôlé, il faut limiter le remplissage au strict maintien des objectifs de pression artérielle recommandés. Donc ici vu la stabilisation hémodynamique il ne faut pas remplir ce patient (risque de dilution ++ des facteurs de coagulation et des plaquettes/GR).

Aucune place des solutés hypotoniques si traumatisme crânien grave (y compris Ringer Lactate)

HEA que **lorsque l'utilisation des cristalloïdes seuls est jugée insuffisante** pour maintenir la volémie et en l'absence de leurs contre-indications. Dose la plus faible possible et la durée d'administration la plus courte possible. **Donc ici non nécessaire.** Il n'existe pas assez d'études de bonne qualité pour savoir si ces recommandations doivent s'étendre aux autres colloïdes semi-synthétiques.

Aucune place de l'albumine dans la prise en charge des patients en choc hémorragique. DONC AU TOTAL : Garde veine de NaCl 0.9%, pas de remplissage.

Avant le départ au scanner vous récupérez les résultats de la radiographie thoracique (sans particularités), de la radiographie du bassin et vous réalisez une FAST écho qui ne retrouve pas d'épanchement péritonéal. L'Hemocue est à 5,7 g/dl d'Hb.

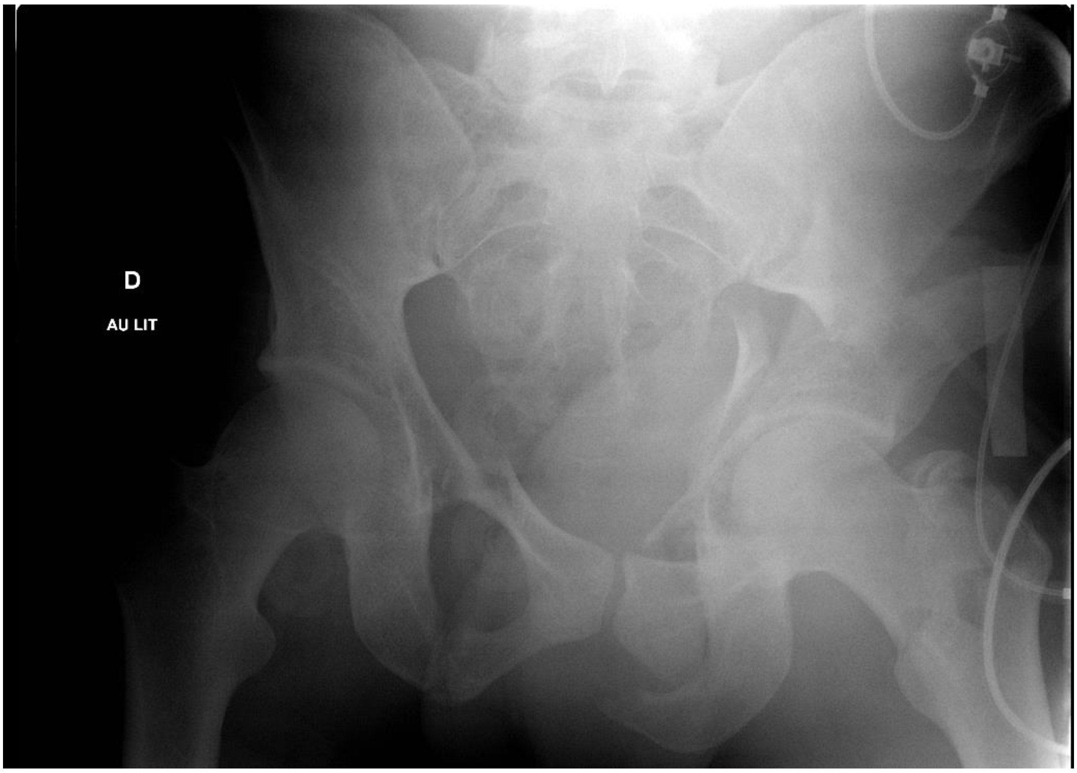

4-Que faites-vous ? Quel geste est formellement contre-indiqué ?

Patient stabilisé sur le plan hémodynamique = TDM injectée corps entier

Transfusion avec un ratio PFC/CGR compris entre 1/ 2 et 1/1

Sondage urinaire formellement contre-indiqué

L'examen réalisé retrouve une rupture de stabilité de l'anneau pelvien, un hématome rétro-péritonéal, une extravasation de produit de contraste iodé faisant suspecter un saignement artériel actif. Il n'y a pas de lésion urologique, ni de lésion viscérale pelvienne. Le reste de l'examen est sans particularité en dehors de quelques pétéchies cérébrales diffuses avec œdème cérébral minime.

5-Que devez-vous envisager sur le plan thérapeutique ?

Situation avec un patient stable ou stabilisé sur le plan hémodynamique sans lésion abdominale (c'est-à-dire sans indication à une chirurgie urgente) et fracture du bassin hémorragique = artério-embolisation immédiatement après le scanner. Pas de signes de gravité clinique au niveau neurologique (Glasgow > 8, amélioration neuro au cours de la prise en charge) donc pas d'indication à une prise en charge neurochirurgicale ou à un monitorage de pression intracrânienne mais surveillance neuro rapprochée et contrôle des ACSOS (cf. enseignements de neuro-réanimation).

Dans le même temps vous récupérez les résultats du bilan biologique prélevé à l'arrivée : Hb 6.1 g/dL, TP 50%, TCA 1.30, fibrinogène 0.9 g/L, PaO2 80mmHg, PaCO2 30 mmHg, HCO3- 16 mmol/L, lactatémie artérielle 5mmol/L, hyperkaliémie 5.6 mmol/L, natrémie 144 mmol/L. Pas de trouble du bilan hépatique.

6- Quel marqueur permet d'évaluer le degré d'hypoperfusion tissulaire et la sévérité du choc hémorragique ?

Concentration du Lactate artériel

Cas clinique Semaine 3 : **<u>CHOC HEMORRAGIQUE CHEZ LE PATIENT SOUS ANTICOAGULANTS ORAUX</u>**

Patient de 75 ans victime d'un AVP piéton versus VL. Ce patient est porteur d'une valve mécanique aortique et est traité par COUMADINE au long cours.

PA = 115/64 mmHg, FC = 110/min, SPO2 = 98% sous O2 3L/min aux lunettes, score de Glasgow à 15/15, patient calme et coopérant.

Au bilan lésionnel, vous retrouvez une défense de l'hypochondre droit et une fracture de l'avant-bras gauche. Il n'y a pas d'argument pour un traumatisme crânien associé.

1 - Quel examen iconographique est à réaliser en urgence ? Quels examens biologiques demandez-vous ?

FAST-échographie : recherche d'un épanchement liquidien intra-abdominal, d'un épanchement gazeux ou liquidien thoracique, évaluation du péricarde et du myocarde, +/- recherche de signes d'HTIC au doppler trans-crânien (ici moins pertinent car examen neurologique normal *a priori*).

Devant l'absence de point d'appel clinique pour une fracture du bassin ou un traumatisme thoracique chez un patient stable sur le plan hémodynamique, conscient et orienté (interrogatoire fiable), il est envisageable de ne pas réaliser de radiographies thorax / bassin (habitudes de centre).

Biologie :

Bilan hémostase-coag : NFS-TP-TCA-Fibrinogène-INR (car traitement par AVK)

Bilan pré-transfu : Gr-Rh x 2 + RAI

GDS-Lactates, CPK, Ionogramme avec urée et créatinine, bilan hépatique (foie de choc ? traumatisme abdo ?).

Troponine (contusion myocardique ?)

ECG systématique chez le traumatisé +++,

Lipase (contusion pancréatique ? témoin de la gravité du traumatisme abdominal)
Selon contexte : alcool, toxiques,…

La FAST ne retrouve pas d'épanchement. L'INR réalisé à l'arrivée est à 2,5.

2 - Quelle est votre attitude diagnostique ?

Stabilité hémodynamique permettant le transfert du patient au scanner pour un scanner injecté corps entier (temps artériel, portal, veineux).

Il est mis en évidence un saignement artériel actif hépatique avec la présence d'un hématome sous-capsulaire du foie. Le patient est toujours stable hémodynamiquement.

3 - Comment gérez-vous l'hémostase biologique chez ce patient ?

Objectif INR < 1.5

Traitement par vitamine K 10 mg IVL.

Traitement par CCP (concentré de complexe prothrombinique) 25 U/kg en absence d'INR. Ici l'INR est à 2,5.

Par exemple selon le RCP du CCP OCTAPLEX, avec un INR à 2.5, il est préconisé d'administrer des CCP entre 0.9 -1.3 mL d'OCTAPLEX par kg de poids corporel.

Traitement habituel du choc hémorragique :

Transfusion de CGR et PFC isogroupe iso rhésus en urgence vitale (ratio 1/1 ou 1/2) si besoin selon taux d'Hb,

Exacyl si traumatisme <3H,

Fibrinogène selon fibrinogénémie,

Expansion volémique et Noradrénaline selon les objectifs tensionnels.

4 - Quelle sera votre attitude thérapeutique étiologique?

Devant un patient stable hémodynamiquement avec la présence d'un saignement actif artériel hépatique : indication à une artériographie avec embolisation en radiologie interventionnelle.

En artériographie, le radiologue ne retrouve pas de saignement actif et ne réalise donc aucune embolisation. Six heures après le traumatisme, le patient se dégrade brutalement sur le plan hémodynamique (PA = 77/34 mmHg, FC = 143/min) avec pâleur, marbrures et douleur abdominale brutale et intense.

5 - Quel est le diagnostic le plus probable ? Quelle est votre attitude thérapeutique en urgence ?

Probable rupture de la capsule hépatique qui faisait hémostase du saignement avec hémopéritoine massif et récidive de l'hémorragie artérielle hépatique.

Renforcer l'hypothèse diagnostique: FAST échographie (épanchement abdominal important)

Remplissage +/- Noradrénaline QSP PAM 60 mmHg (pas plus pour éviter de majorer l'hémorragie)

Transfusion massive (ratio CGR/PFC à 1 :1 ou 1 :2 en débutant par ex par 3-4 CGR + 3-4 PFC +/- 1 MCP ou CPA selon le taux de plaquette initial + Calcium pour maintenir une calcémie ionisée > 0.9 mmol/l).

Vérifier efficacité des PPSB : nouveau bilan complet avec (entre autres) TP et INR.

Chirurgie en urgence pour packing hépatique (« damage control ») puis nouvelle artériographie pour embolisation.

ENGLISH TRADUCTION

Clinical case Week 1: HEMOSTASE BASIS AND RESUSCITATION OF HEMORRAGIC SHOCK

53-year-old patient, fall from a 4-metre scaffold. Injury report: deformation of the left lower limb (thigh increased in volume). No other visible lesions. He takes KARDEGIC and PLAVIX "for the heart". Vitals: HR 138/min- BP 87/43 mmHg - GCS 15/15 - SpO2 100% within 6l/min O2. You suspect an hemorrhagic shock secondary to a closed fracture of the left femur.

1 - In this patient in a state of hemorrhagic shock, what will be your blood pressure objectives?

SBP 80-90 mmHg OR MAP between 60- 65 mmHg in patients WITH NO head injury

Tolerate hypotension to minimize the risk of bleeding aggravation until surgical and/or radio-interventional hemostasis is achieved.

Note: For patients with severe head injury (Coma Glasgow Score 8) with haemorrhage shock, MAP > 80 mmHg, before brain monitoring is available, despite the risk of aggravated bleeding.

2 - What treatment should you provide within 3 hours of the trauma (+ dosage)? What is its mechanism of action?

Tranexamic acid (EXACYL®): 1 g IV over 10 minutes then relay 1g IV over 8 hours.

Mechanism of action: anti-fibrinolytic molecule. Synthetic lysine analog leading to a decrease in the transformation of plasminogen to plasmin => fibrinolysis inhibition.

3 - Is there a contraindication to this drug in this potentially coronary patient?

NO, No contraindications in this patient. Benefits of Tranexamic acid treatment even for the patient with coronary disease.

Reference: Effects of tranexamic acid on death, vascular occlusive events, and blood transfusion in trauma patients with significant haemorrhage (CRASH-2): a randomised, placebo-controlled trial - Lancet - 2010. Tranexamic acid significantly reduces all-cause mortality (RR = 0.91; 95% CI = 0.85-0.97), particularly bleeding mortality (RR = 0.85; 95% CI = 0.76-0.96). The rate of thrombotic events is not increased by this treatment, the rate of myocardial infarction is even reduced.

After vascular filling (1000 ml of crystalloids) he is hemodynamically stabilized. At Hemocue, Hb = 7g/dL. You learn that this patient has an active stent since 6 months.

4 - What will be your transfusion goal for this patient?

Hemoglobin objective between 9-10 g/dL (coronary disease).

5 - What will be your first prescription for transfusion (type and quantity of blood products)?

As an example:

-2 to 3 RBC red blood cell concentrates.

-2 to 3 FFP fresh frozen plasma.

-1 mixture of PC platelet concentrates for platelet dysfunction induced by the long-term use of Kardegic and Plavix and the context of hemorrhagic shock.

The biological resultts are: Hemoglobin 7.6 g/dL, Platelets 280 G/L, PT 80%, aPTT 1.12, Fibrinogenemia at 0.9 g/L

6 - What additional treatment will you implement (+ dosage)? With what objective?

Fibrinogen 3 g (dosage for an adult of 70 kg), no bolus, IV over 10-20 min, on PVC (risk of fibrinogen accumulation in left auricle if CVC), Fibrinogenemia objective > 1.5-2 g/L.

The transfusion of FFP will also help to correct hypofibrinogenemia but in a LIMITED way. Plasma provides all plasma proteins, especially coagulation factors and complement fractions. The preservation of coagulation factor activity is controlled by measuring the concentration of factor VIII and fibrinogen. The French standard for factor VIII requires that it be at least 0.7 IU/mL for FFP-Se and at least 0.5 IU/mL for FFP-IA, FFP-SD and lyophilized plasma. Plasma is the only product able to provide factor V, protein S, plasminogen and metalloprotease cleaving factor Von Willebrand (ADAMTS 13), as well as some fractions of the complement. These factors are not available in stable purified form among blood-derived drugs.

7- What is the mechanism of hypofibrinogenemia?

Along with platelets, fibrinogen, a precursor to fibrin, is an essential component in the development of a solid hemostatic thrombus. A decrease in fibrinogenemia is common in hemorrhagic shocks.

Fibrinogen deficiency is particularly marked in the case of dilution, and may also have other causes, including fibrinolysis as part of post-traumatic coagulopathy:

Transformation of plasminogen to plasmin following release of tissue plasminogen activator (t-PA) + Inhibition of plasminogen activator inhibitor (PAI -1) = hyperfibrinolysis

Result = degradation of fibrin by plasmin; consumption of fibrinogen = **hypofibrinogenemia**

Clinical case Week 2: TRAUMATIC POST HEMORRAGIC SHOCK

20-year-old patient, victim of a motorcycle/vehicle accident. Examination: BP = 90/50 (MAP 63) mmHg, HR 120/min, Glasgow 11/15, SpO2 100% with O2 6L/min. VAS 8/10, T° = 35.2°C, symmetrical isocores pupils, pelvic deformation with suspicion of pelvic fracture, frontal ecchymosis with a notion of head trauma (2 min. loss of consciousness).

1-What are the therapies that should be started in the pre-hospital setting? What is the mean blood pressure target for this patient?

Immobilization of the head-neck-trunk axis

Pelvic compression belt

2 PVC of good caliber.

If PVC impossible and pre-hospital: intra-bone access >> CVC

Vascular filling with crystalloid solutions in first intention: 1000-1500 ml (free flow)

Analgesia (paracetamol, morphine, ketamine)

Warming up: fight against hypothermia

Tranexamic acid IV 1g over 10 min

MAP target > 80 mmHg (severe head injury with impaired consciousness)

After 1500 mL of crystalloids and the introduction of Norepinephrine (obj MAP > 80 mmHg) the constants are: BP 112/63 (PAM 79) mmHg, HR 114 bpm, Glasgow 13/15, VAS 2/10, SpO2 100% with O2 6L/min.

2-What additional diagnostic tests do you perform in the ER ?

First intention: FAST-ultrasound (search for intra-abdominal fluid effusion, gas or thoracic fluid effusion, evaluation of the pericardium and myocardium, search for signs of elevated intracranial pressusre at the trans-cranial doppler) + Chest x-ray (search for gas or thoracic fluid effusion, enlargement of the mediastinum (isthm rupture?), costal fractures) + Pelvis radiography (search for a fractured +/- unstable pelvis)

Second intention: In view of the stabilization of the patient who clinically presents at least two life-threatening conditions (pelvis and head injury), it is reasonable to quickly organize a body-CT for the lesion assessment. If peripheral bone fracture, consider targeted bone radiographs.

3-Do you carry out a new vascular filling? The nurse asks you which infusion solution you want to perfuse during TDM, which one do you choose? You have at your disposal: 5% serum glucose, NaCl 0.9%, Ringer lactate, hydroxyethyl starch-based solutions (Voluven ®), albumin-based solutions 20%.

Non-hypotonic crystalloid solutions (NaCl 0.9% or Isofundine) and until bleeding is controlled, filling should be limited to strictly maintaining the recommended blood pressure objectives. So here, given

the hemodynamic stabilization, this patient should not be filled (risk of dilution ++ of coagulation factors and platelets/RBC).

No place for hypotonic solutes if severe head injury (including Ringer Lactate)

HES only when the use of crystalloids alone is considered insufficient to maintain blood volume and in the absence of their contraindications. The lowest possible dose and the shortest possible administration time. So here not necessary. There are not enough good quality studies to know whether these recommendations should be extended to other semi-synthetic colloids.

No place for albumin in the management of patients in hemorrhagic shock.

IN CONCLUSION: NaCl vein guard 0.9%, no vascular filling.

Before leaving for the CT scan, you collect the results of the chest X-ray (without any particularities), the pelvis X-ray (cf figure) and you perform a FAST echo which does not find any peritoneal effusion. Hemocue is 5.7 g/dl Hb.

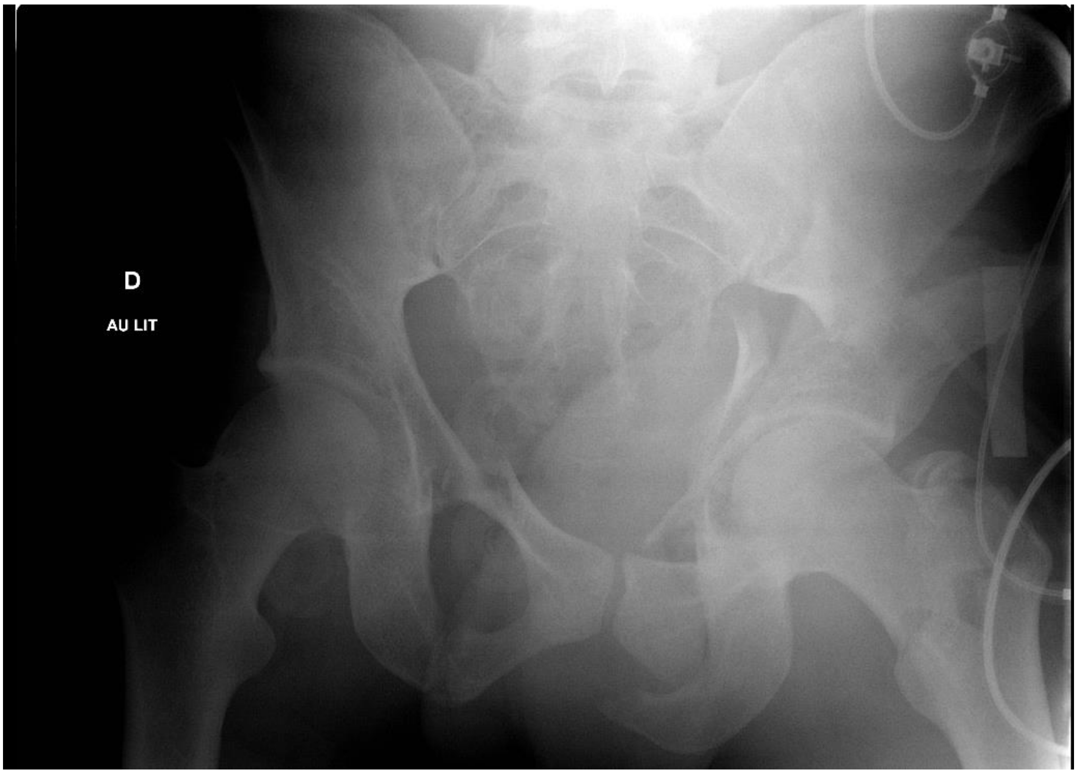

4- What are you doing? What action is formally contraindicated?

Hemodynamically stabilized patient = injected whole body CT

Transfusion with a FFP/RBC ratio between 1/ 2 and 1/1

Formally contra-indicated : urinary catheterization

The CT found a ruptured pelvic ring (instable), a retroperitoneal hematoma, an extravasation of iodinated contrast material suggesting an active arterial bleeding. There is no urological lesion or pelvic visceral lesion. The rest of the examination is normal except for a few diffuse cerebral petechiae with minimal cerebral edema.

5-What therapeutic should you consider ?

Situation with a hemodynamically stable or stabilized patient without abdominal injury (i.e., without indication for urgent surgery) and hemorrhagic fracture of the pelvis = arterioembolization immediately after the CT. No signs of neurological severity (Glasgow > 8, neuro improvement during initial management) so no indication for neurosurgical management or intracranial pressure monitoring but close neuro-monitoring.

At the same time you collect the results of the biological assessment taken at the arrival: Hb 6.1 g/dL, PT 50%, aPTT 1.30, fibrinogen 0.9 g/L, PaO_2 80mmHg, $PaCO_2$ 30 mmHg, HCO_3^- 16 mmol/L, arterial lactatemia 5mmol/L, hyperkalaemia 5.6 mmol/L, natremia 144 mmol/L. No liver function disorders.

6- What marker can be used to assess the degree of tissue hypoperfusion and the severity of hemorrhagic shock?

Concentration of arterial lactate

Clinical case Week 3: HEMORRAGICAL SHOCK IN THE PATIENT WITH ORAL ANTICOAGULANTS

75-year-old patient with a pedestrian versus vehicle accident. This patient has an aortic mechanical valve and is treated with COUMADINE.

BP = 115/64 mmHg, HR = 110/min, SPO2 = 98% with O2 3L/min, Glasgow score at 15/15, patient calm and cooperative.

At the lesion assessment, there is a defence of the right hypochondrium and a fracture of the left forearm. There is no argument for an associated head injury.

1 - What iconographic examination is urgently needed? What biological tests do you require?

FAST-ultrasound: search for intra-abdominal fluid effusion, gas or thoracic fluid effusion, evaluation of the pericardium and myocardium, +/- search for signs of elevated intra-cranial pressusre on trans-cranial Doppler (here less relevant because normal neurological examination).

In the absence of a clinical pelvic fracture or chest injury in a hemodynamically stable, conscious and oriented patient (reliable interview), it is possible not to perform chest / pelvis radiographs (according to the team's habits).

Biology :

Hemostasis coag assessment: NFS-PT-aPTT-Fibrinogen-INR (because treatment with VKA)

Pre-transfusion tests: Gr-Rh x 2 + search for irregular agglutinins

blood gas-Lactates, CPK, Ionogram with urea and creatinine, liver check-up (shock liver ? abdo trauma ?).

Troponin (myocardial contusion?)

Systematic ECG in trauma patients ++++,

Lipase (pancreatic contusion? witness of the severity of the abdominal trauma) According to context: alcohol, toxic,...

FAST does not find any effusion. The INR performed on arrival is 2.5.

2 - What is your diagnostic attitude?

Hemodynamic stability allowing the patient to be transferred to the scanner for a whole body injected scanner (arterial, portal, and venous time).

Active hepatic arterial bleeding is detected with the presence of a subcapsular hematoma of the liver. The patient is still hemodynamically stable.

3 - How do you manage biological hemostasis in this patient?

INR target < 1.5

Treatment with vitamin K 10 mg IV.

Treatment with PCC (prothrombin complex concentrate) 25 U/kg in the absence of INR. Here the INR is 2.5.

For example, for OCTAPLEX PCC, with an INR of 2.5, it is recommended to administer PCC between 0.9 -1.3 mL of OCTAPLEX per kg body weight.

Usual treatment of hemorrhagic shock:

Transfusion of RBC and FFP isogroup iso rhesus in vital emergency (ratio 1/1 or 1/2) if necessary according to Hb level,

Exacyl if trauma <3H,

Fibrinogen according to fibrinogenemia,

Volemic expansion and Norepinephrine according to blood pressure objectives.

4 - What will be your etiological therapeutic attitude?

Hemodynamically stable patient with the presence of an active hepatic arterial bleeding: indication for arteriography with embolization in interventional radiology.

In arteriography, the radiologist does not find any active bleeding and therefore does not perform any embolization. Six hours after the trauma, the patient experienced sudden hemodynamic deterioration (BP = 77/34 mmHg, HR = 143/min) with pallor, mottling and sudden and severe abdominal pain.

5 - What is the most likely diagnosis? What is your emergency therapeutic attitude?

Probable rupture of the hepatic capsule that caused haemostasis of the bleeding with massive haemoperitoneum and recurrence of hepatic arterial hemorrhage.

Reinforce the diagnostic hypothesis: FAST ultrasound (significant abdominal effusion)

Vascular filling +/- Norepinephrine for MAP at 60 mmHg (no more to avoid increasing bleeding)

Massive transfusion (RBC/FFP ratio at 1:1 or 1:2 starting e. g. with 3-4 RBC + 3-4 FFP +/- 1 PC depending on the initial platelet rate + Calcium to maintain ionized calcium > 0.9 mmol/l).

Verify the effectiveness of the PCC: new complete assessment with (among others) PT and INR.

Emergency surgery for hepatic packing ("damage control") then new arteriography for embolization.
