# Supplementary material for: Use of the Smartphone App WhatsApp as an E-Learning Method for Medical Residents: Multicenter Controlled Randomized Trial
Source: JMIR Mhealth Uhealth. 2019 Apr 9;7(4):e12825. doi: 10.2196/12825 (PMC6477573; doi:10.2196/12825)
Supplement: Multimedia Appendix 3 [file mhealth_v7i4e12825_app3.pdf]

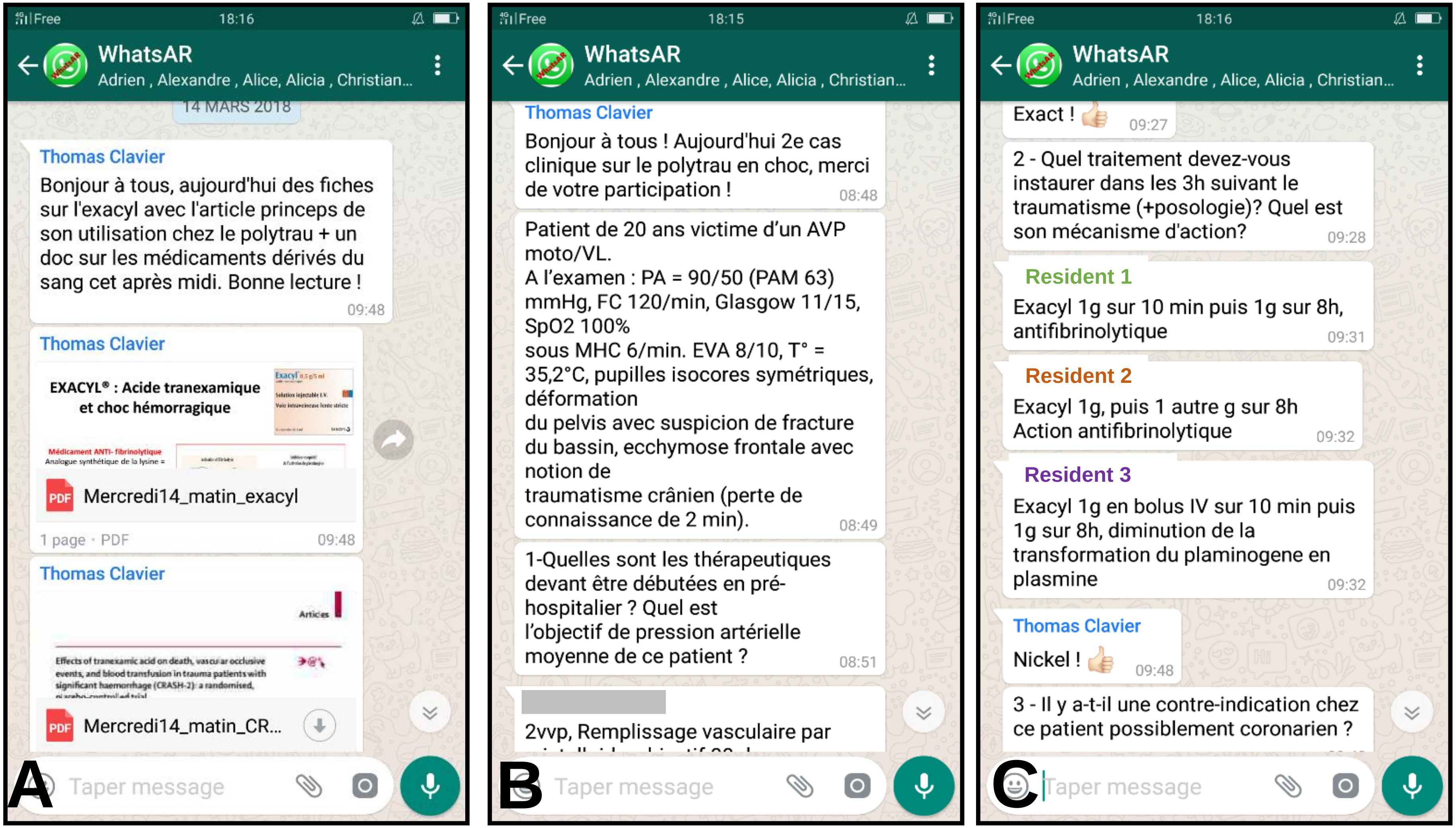

Supplemental Digital Content 1: Several examples of the use of WhatsApp® for learning purposes during the protocol (in French). (A) direct communication of teaching documents (from Monday to Thursday), **(B)** questions from a senior anesthesiologist (TC) to residents (Friday clinical cases), **(C)** answers from the residents (anonymized) with feedback and validation of the senior anesthesiologist.

Transcript in English of WhatsApp images from Supplemental data S2

Fig 3A:

TC : “Hello everyone, today’s topic is tranexamic acid with the Princeps article on its use in the traumatized patient + a paper on blood-derived drugs this afternoon. Good reading!”

Fig 3B:

TC: “Hello everyone! Today’s 2nd clinical case concerns the trauma patient in shock, thank you for your participation!”

TC: “20-year-old patient, victim of a motorcycle accident on the public highway.

Clinical examination: blood pressure at 90/50mmHg (MAP 63 mmHg), heart rate at 120/min, Glasgow score 11/15, Spo2 100% under high concentration O2 mask at 15 L/min, VAS 8/10, Temperature 35.2°C, isocorous and symmetrical pupils, pelvic deformation with suspicion of pelvic fracture, frontal ecchymosis with notion of cranial trauma (loss of consciousness of 2 min).”

TC: “1-What treatments should be started during pre-hospital management? What is the mean arterial blood pressure target for this patient?”

Fig 3C:

TC: “2- What treatment should be started within 3 hours of the trauma (with dosage)? What is its mechanism of action?”

Resident 1: “Exacyl [tranexamic acid] 1g for 10 minutes then 1g for 8 hours, antifibrinolytic.”

Resident 2: “Exacyl [tranexamic acid] 1g then 1 other g over 8 hours. Antifibrinolytic action.”

Resident 3: “Exacyl [tranexamic acid] 1g in IV bolus for 10 min then 1g for 8h, decrease in the transformation of plasminogen into plasmin.”

TC: “Very good!”

TC: “3- Is there a contraindication [to tranexamic acid] in this patient with possible coronary disease ?”
