# Supplementary material for: Use of the Smartphone App WhatsApp as an E-Learning Method for Medical Residents: Multicenter Controlled Randomized Trial
Source: JMIR Mhealth Uhealth. 2019 Apr 9;7(4):e12825. doi: 10.2196/12825 (PMC6477573; doi:10.2196/12825)
Supplement: Multimedia Appendix 4 [file mhealth_v7i4e12825_app4.pdf]

CAS CLINIQUE n° 1

Vous prenez en charge un patient devant être opéré en urgence pour hémopéritoine après un accident de la voie publique.

<table>
<tr><th></th><th>L'attitude envisagée est</th><th>Mais vous apprenez que</th><th>L'attitude envisagée devient</th></tr>
<tr><td>TCS 01</td><td>Administrer de l'acide tranexamique</td><td>Antécédent de thrombose veineuse profonde</td><td rowspan="3">+2 quasi certaine
+1 plus probable
0 ni plus ni moins probable
-1 moins probable
-2 quasi improbable</td></tr>
<tr><td>TCS 02</td><td>Demander en urgence vitale des CGR et des PFC</td><td>L'Hb est à 10g/dL</td></tr>
<tr><td>TCS 03</td><td>Administrer du fibrinogène</td><td>La concentration plasmatique est de 3 g/L</td></tr>
</table>

CAS CLINIQUE n° 2

Vous êtes appelé au SAS de déchocage pour participer à la prise en charge périopératoire d'un adulte traumatisé sévère suite à un AVP. Il a déjà reçu 8 culots globulaires et l'hémodynamique reste instable.

<table>
<tr><th></th><th>L'attitude envisagée est</th><th>Mais vous apprenez que</th><th>L'attitude envisagée devient</th></tr>
<tr><td>TCS 04</td><td>D'opter pour un schéma transfusionnel 1 PFC/1CG</td><td>L'hémoglobine est à 10g/dL</td><td rowspan="3">+2 quasi certaine
+1 plus probable
0 ni plus ni moins probable
-1 moins probable
-2 quasi improbable</td></tr>
<tr><td>TCS 05</td><td>D'opter pour l'administration de fibrinogène + acide tranexamique</td><td>Le temps de céphaline avec activateur est à 1,5 fois le témoin</td></tr>
<tr><td>TCS 06</td><td>De rétablir la volémie avec une Hb cible à 8g/dL</td><td>Le patient a un antécédent d'angor stable</td></tr>
</table>

CAS CLINIQUE n° 3

Vous prenez en charge un patient de 35 ans après un AVP. Il est conscient, GCS à 12 et présente une contracture abdominale + des fractures périphériques

	L'attitude envisagée est	Mais vous apprenez que	L'attitude envisagée devient
**TCS 07**	Administrer du fibrinogène	La concentration plasmatique de fibrinogène est de 1,3 g/L	+2 quasi certaine<br>+1 plus probable<br>0 ni plus ni moins probable<br>-1 moins probable<br>-2 quasi improbable

CAS CLINIQUE n° 4

Vous prenez en charge un patient de 66 ans après une chute d'arbre (4 m). Il est conscient, GCS à 15, FR à 30/min et présente des douleurs pelviennes et une fracture du fémur gauche. Sa FC est à 115/min et sa PA à 102/56 mmHg.

<table>
<tr><th></th><th>L'attitude envisagée est</th><th>Mais vous apprenez que</th><th>L'attitude envisagée devient</th></tr>
<tr><td>TCS 08</td><td>Embolisation du bassin devant une fracture complexe</td><td>Absence de fuite de produit de contraste au body-TDM</td><td rowspan="2">+2 quasi certaine
+1 plus probable
0 ni plus ni moins probable
-1 moins probable
-2 quasi improbable</td></tr>
<tr><td>TCS 09</td><td>Administrer un concentré plaquettaire (MCP ou CPA)</td><td>Le patient est sous anti-aggrégation plaquettaire par Kardegic ® (aspirine)</td></tr>
</table>

CAS CLINIQUE n° 5

Vous prenez en charge un patient de 25 ans après pour blessure thoracique par arme blanche. Le point d'entrée de la lame est antérieur en regard du 3 espace inter-costal droit. Il est conscient, GCS à 12, FR à 40/min, FC 135/min, PA à 80/42 mmHg.

<table>
<tr><th></th><th>L'attitude envisagée est</th><th>Mais vous apprenez que</th><th>L'attitude envisagée devient</th></tr>
<tr><td>TCS 10</td><td>Transfusion de 3 CGR et 3 PFC</td><td>L'Hb est à 9 g/dl, le TP à 60%</td><td rowspan="2">+2 quasi certaine
+1 plus probable
0 ni plus ni moins probable
-1 moins probable
-2 quasi improbable</td></tr>
<tr><td>TCS 11</td><td>Clamper le drain thoracique après la pose</td><td>Le drain a donné 1200 ml de sang frais</td></tr>
</table>

CAS CLINIQUE n° 6

Vous prenez en charge un patient de 73 ans après un AVP piéton / VL. Il a été intubé par le SMUR sur un tableau de GCS initial à 8 avec anisocorie. Il est tachycarde à 130/min.

<table>
<tr><th></th><th>L'attitude envisagée est</th><th>Mais vous apprenez que</th><th>L'attitude envisagée devient</th></tr>
<tr><td>TCS 12</td><td>Administrer 500 ml de remplissage pas NaCl 0;9%</td><td>Il vient de recevoir 200 ml de Mannitol 20% par le SMUR</td><td rowspan="3">+2 quasi certaine
+1 plus probable
0 ni plus ni moins probable
-1 moins probable
-2 quasi improbable</td></tr>
<tr><td>TCS 13</td><td>Réaliser une FAST-échographie avant la TDM</td><td>Sa PA est à 94/42 mmHg</td></tr>
<tr><td>TCS 14</td><td>Emboliser une fracture de bassin avec saignement actif</td><td>Hématome extra-dural frontal gauche avec engagement sous-falcoriel à la TDM</td></tr>
</table>

CAS CLINIQUE n° 7

Vous prenez en charge un patient de 63 ans après une agression. Il est traité par anti-vitamines K (Previscan ®) et par IEC. Il présente un épistaxis abondant et des douleurs costales droite avec fractures de cotes et hémothorax de moyenne abondance à la TDM.

<table>
<tr><th></th><th>L'attitude envisagée est</th><th>Mais vous apprenez que</th><th>L'attitude envisagée devient</th></tr>
<tr><td>TCS 15</td><td>Antagoniser les AVK par PPSB à 25 UI/kg</td><td>Il est traité par AVK depuis 2 mois pour une embolie pulmonaire grave</td><td rowspan="2">+2 quasi certaine
+1 plus probable
0 ni plus ni moins probable
-1 moins probable
-2 quasi improbable</td></tr>
<tr><td>TCS 16</td><td>Administrer 2g de Calcium</td><td>Le patient a reçu 2 GCR</td></tr>
</table>

CAS CLINIQUE n° 8

Vous prenez en charge un patient de 78 ans après une chute d'échelle dans son garage où il est resté au sol durant 6h. Il est traité par Eliquis ® (Apixaban) pour une FA ancienne. Il est stable sur le plan hémodynamique (FC 110/min, PA 98/53 mmHg) mais présente une douleur de l'hypochondre gauche.

<table>
<tr><th></th><th>L'attitude envisagée est</th><th>Mais vous apprenez que</th><th>L'attitude envisagée devient</th></tr>
<tr><td>TCS 17</td><td>Antagoniser l'Apixaban par PPSB (concentrés de complexe prothrombinique non activé)</td><td>Vous avez à disposition dans votre hôpital du concentré de complexe prothrombinique activé FEIBA®</td><td rowspan="3">+2 quasi certaine
+1 plus probable
0 ni plus ni moins probable
-1 moins probable
-2 quasi improbable</td></tr>
<tr><td>TCS 18</td><td>Réaliser un body-TDM</td><td>La FAST-échographie retrouve un hémopéritoine de moyenne abondance</td></tr>
<tr><td>TCS 19</td><td>Splénectomie en urgence</td><td>Fracture de rate grade 2 sans saignement actif à la TDM</td></tr>
</table>

CAS CLINIQUE n° 9

Vous prenez en charge un patient de 27 ans après un AVP VL haute cinétique. Il a été intubé à l'arrivée du SMUR pour une détresse respiratoire aigue et des troubles de la conscience avec état de choc. Sa PA est à 65/28 mmHg et sa FC à 169/min à l'arrivée au déchocage.

<table>
<tr><th></th><th>L'attitude envisagée est</th><th>Mais vous apprenez que</th><th>L'attitude envisagée devient</th></tr>
<tr><td>TCS 20</td><td>Débuter de la Noradrénaline</td><td>La SpO2 est à 89% en FiO2 100%</td><td rowspan="2">+2 quasi certaine
+1 plus probable
0 ni plus ni moins probable
-1 moins probable
-2 quasi improbable</td></tr>
<tr><td>TCS 21</td><td>Administrer du NovoSeven ® (facteur VII activé)</td><td>Une première transfusion de 4 CGR + 4 PFC est en cours</td></tr>
</table>

CAS CLINIQUE n° 10

Vous prenez en charge une patiente de 42 ans après un AVP. Elle présente une fracture des 2 fémurs et une douleur abdominale diffuse. L'hémoglogine est à 8 g/dl, la FC à 130/min et la PA à 87/47 mmHg. Dans son traitement on retrouve du Xarelto ® (Rivaroxaban) pour une phlébite récente.

	L'attitude envisagée est	Mais vous apprenez que	L'attitude envisagée devient
**TCS 22**	Antagoniser le Rivaroxaban par concentré de complexe prothrombinique activé FEIBA®	Le TP est à 76% et le TCA à 1,23	+2 quasi certaine<br>+1 plus probable<br>0 ni plus ni moins probable<br>-1 moins probable<br>-2 quasi improbable
**TCS 23**	Transfuser un concentré plaquettaire	Le taux de plaquettes est à 132000/mm3	

CAS CLINIQUE n° 11

Vous prenez en charge une patiente de 81 ans après une chute d'un dans son jardin (réception sur une barre métallique). Elle présente une fracture fermée du fémur gauche et une douleur lombaire gauche. Le body-TDM retrouve une fracture rénale grade 4, la patiente est stable sur le plan hémodynamique.

<table>
<tr><th></th><th>L'attitude envisagée est</th><th>Mais vous apprenez que</th><th>L'attitude envisagée devient</th></tr>
<tr><td>TCS 24</td><td>Prise en charge immédiate au bloc de sa fracture du fémur</td><td>La température de la patiente est de 34,8 °C</td><td rowspan="3">+2 quasi certaine
+1 plus probable
0 ni plus ni moins probable
-1 moins probable
-2 quasi improbable</td></tr>
<tr><td>TCS 25</td><td>Embolisation de la fracture rénale</td><td>Fuite de produit de contraste au temps artériel du body TDM</td></tr>
<tr><td>TCS 26</td><td>Remplissage de 500 ml de NaCl 0,9%</td><td>La PA est à 98/56 mmHg</td></tr>
</table>

CAS CLINIQUE n° 12

Vous prenez en charge un patient de 38 ans qui a chuté de moto-cross (non casqué). Il a été intubé par le SMUR devant un état de choc avec troubles de la conscience. Sa PA est à 96/55 mmHg sous noradrénaline 0,05 µg/kg/min après 1500 ml de remplissage, sa FC à 110/min. Il présente une plaie du scalp de 15 cm avec saignement abondant.

<table>
<tr><th></th><th>L’attitude envisagée est</th><th>Mais vous apprenez que</th><th>L’attitude envisagée devient</th></tr>
<tr><td>TCS 27</td><td>Réaliser un body-TDM</td><td>L’hémodynamique reste stable sous noradrénaline</td><td rowspan="3">+2 quasi certaine
+1 plus probable
0 ni plus ni moins probable
-1 moins probable
-2 quasi improbable</td></tr>
<tr><td>TCS 28</td><td>Suturer la plaie du scalp</td><td>Le patient est anisocore</td></tr>
<tr><td>TCS 29</td><td>Artériographie pour fracture hépatique grade IV</td><td>Il présente également une fracture du bassin avec suffusion de produit de contraste. La TDM cérébrale est normale.</td></tr>
</table>

Clinical case 1

You are treating a patient who needs an emergency surgery for hemoperitoneum after a road traffic accident.

<table>
<tr><th></th><th>You plan to :</th><th>But you learn :</th><th>Your original decision becomes :</th></tr>
<tr><td>TCS 01</td><td>Administer tranexamic acid</td><td>History of deep vein thrombosis</td><td rowspan="3">+2 almost certain
+1 more likely
0 unchanged
-1 less likely
-2 unlikely</td></tr>
<tr><td>TCS 02</td><td>Request Packed Red Blood Cells and Fresh Frozen Plasma in “vital emergency”</td><td>Hb level is 10g/dL</td></tr>
<tr><td>TCS 03</td><td>Administer fibrinogen</td><td>Plasma Fibrinogen concentration is 3 g/L</td></tr>
</table>

Clinical case 2

You are part of the team in charge of the perioperative management of a severe traumatized adult following a traffic road accident. He has already been transfused with 8 packed red blood cells (PRBC) but the patient remains hemodynamically unstable.

	You plan to :	But you learn :	Your original decision becomes :
**TCS 04**	Opting for a transfusion blood ratio 1 FFP/1PRBC	Hb level is 10g/dL	+2 almost certain<br>+1 more likely<br>0 unchanged<br>-1 less likely<br>-2 unlikely
**TCS 05**	Administer tranexamic acid and fibrinogen	The APTT is à 1,5 times that of a "normal" control sample	
**TCS 06**	Stabilize a Hb level above 10g/dL	The patient has a history of stable angina	

Clinical case 3

You are taking care of a 35 year-old patient after a road traffic accident. He is conscious, Coma glasgow scale (CGS) is 12. Abdominal examination revealed a defence and he has peripheral bone fractures.

	You plan to :	But you learn :	Your original decision becomes :
**TCS 07**	Administer fibrinogen	Plasma Fibrinogen concentration is 1,3 g/L	+2 almost certain<br>+1 more likely<br>0 unchanged<br>-1 less likely<br>-2 unlikely

Clinical case 4

You are taking care of a 66 year-old patient who that fell down from a tree (heights up to 4m)
He is conscious. Coma glasgow scale is 15. Respiratory rate is 30/min. He has a pelvic pain and an obvious femur fracture.
HR is 115/min and BP is 102/56 mmHg.

	You plan to :	But you learn :	Your original decision becomes :
**TCS 08**	Arterial embolization for pelvic fracture	There is no contrast extravasation on body CT scan	+2 almost certain<br>+1 more likely<br>0 unchanged<br>-1 less likely<br>-2 unlikely
**TCS 09**	Transfusion of Pooled platelets	The patient takes aspirin for angina	

Clinical case 5

You are taking care of a 25 year-old patient after an aggression with penetrating chest trauma (knife). The blade entry point is anterior, on the right hemithorax (third intercostal space).
He is conscious. Coma glasgow scale is 12. Respiratory rate is 40/min, HR is 135/min, BP is 80/42 mmHg.

<table>
<tr><th></th><th>You plan to :</th><th>But you learn :</th><th>Your original decision becomes :</th></tr>
<tr><td>TCS 10</td><td>Transfusion of 3 PRBC and 3 FFP</td><td>Hb level is 10g/dL and PT is 60%</td><td rowspan="2">+2 almost certain
+1 more likely
0 unchanged
-1 less likely
-2 unlikely</td></tr>
<tr><td>TCS 11</td><td>Clamping the chest tube after implantation</td><td>The chest tube drained 1200 ml of blood</td></tr>
</table>

Clinical case 6

You are taking care of a 73 year-old patient after a road traffic accident. He has been intubated by the medical team because of an alterated level of consciousness (coma glasgow scale = 8) and an anisocoria. HR is 130/min.

<table>
<tr><th></th><th>You plan to :</th><th>But you learn :</th><th>Your original decision becomes :</th></tr>
<tr><td>TCS 12</td><td>Administration of fluid loading with saline solution (500 ml NaCl 0;9%)</td><td>He just got 200 ml of Mannitol 20% solution</td><td rowspan="3">+2 almost certain
+1 more likely
0 unchanged
-1 less likely
-2 unlikely</td></tr>
<tr><td>TCS 13</td><td>Perform a Focused Assessment Sonography in Trauma (FAST) before the body CT scan</td><td>BP is 94/42 mmHg</td></tr>
<tr><td>TCS 14</td><td>Perform arterial embolization of pelvic fracture with active bleeding</td><td>There is a left frontal extradural hematoma with subfalcine herniation of the brain</td></tr>
</table>

Clinical case 7

You are taking care of a 63 year-old patient after an agression. His usual medication is VKA (Previscan®).
He has an abundant epistaxis and right costal pain.
CT scan show rib fractures and a moderate haemothorax .

<table>
<tr><th></th><th>You plan to :</th><th>But you learn :</th><th>Your original decision becomes :</th></tr>
<tr><td>TCS 15</td><td>Antagonization VKA thanks to the administration of PCC (25 UI/kg)</td><td>He has been treated for 2 months for a massive pulmonary embolism</td><td rowspan="2">+2 almost certain
+1 more likely
0 unchanged
-1 less likely
-2 unlikely</td></tr>
<tr><td>TCS 16</td><td>Administer Calcium</td><td>He received 2 PRBC</td></tr>
</table>

Clinical case 8

You are taking care of a 78 year-old patient after a fall of 3 feet from a scale. He spent 6 hours on floor. His usual medication is Eliquis ® (Apixaban) for a permanent atrial fibrillation.
He is hemodynamically stable. HR is 110/min and BP is 98/53 mmHg. He has a left upper-quadrant pain.

	You plan to :	But you learn :	Your original decision becomes :
**TCS 17**	Antagonization apixaban thanks to the administration of PCC	You got activated prothrombin complex concentrate FEIBA® in your unit	+2 almost certain<br>+1 more likely<br>0 unchanged<br>-1 less likely<br>-2 unlikely
**TCS 18**	Perform a body CT scan	The Focused Assessment Sonography in Trauma (FAST) shows a moderate hemoperitoneum	
**TCS 19**	Emergency splenectomy	Spleen rupture (grade 2) without active contrast extravasation on CT scan	

Clinical case 9

You are taking care of a 27 year-old patient envolved in a high speed car accident. He has been intubated by the medical team for a respiratory distress, an alterated state of consciousness and he is hemodynamically unstable. BP is 65/28 mmHg and HR is 169/min at hospital arrival.

<table>
<tr><th></th><th>You plan to :</th><th>But you learn :</th><th>Your original decision becomes :</th></tr>
<tr><td>TCS 20</td><td>Initation of Norepinephrine</td><td>SpO2 is 89% with FiO2 100%</td><td rowspan="2">+2 almost certain
+1 more likely
0 unchanged
-1 less likely
-2 unlikely</td></tr>
<tr><td>TCS 21</td><td>Administer NovoSeven ® (activated VII factor)</td><td>First transfusion of 4 PRBC and 4 FFP is occuring</td></tr>
</table>

Clinical case 10

You are taking care of a 42 year-old patient after a road traffic accident. She has a diffuse abdominal pain and bilateral femur fractures.
Hb level is 8 g/dL, HR is 130/min and BP is 87/47 mmHg.
Her usual medication contains Xarelto® (Rivaroxaban) for recurrent venous thromboembolism.

	You plan to :	But you learn :	Your original decision becomes :
**TCS 22**	Antagonization of rivaroxaban by activated prothrombin complex concentrate FEIBA®	PT is 76% and APTT is 1,23	+2 almost certain<br>+1 more likely<br>0 unchanged<br>-1 less likely<br>-2 unlikely
**TCS 23**	Transfusion of pooled platelet	Platelet level is 132000/mm3	

Clinical case 11

You are taking care of a 81 year-old patient after a fall in her garden. She falled on a metal material. She has a close left femoral fracture and a left lumbar pain. The body CT scan shows a left kidney rupture (grade 4). She is hemodynamically stable.

<table>
<tr><th></th><th>You plan to :</th><th>But you learn :</th><th>Your original decision becomes :</th></tr>
<tr><td>TCS 24</td><td>Emergency surgery for the femoral fracture</td><td>Temperature is 34,8 °C</td><td rowspan="3">+2 almost certain
+1 more likely
0 unchanged
-1 less likely
-2 unlikely</td></tr>
<tr><td>TCS 25</td><td>Kidney embolization</td><td>Arterial extravasation contrast on CT scan</td></tr>
<tr><td>TCS 26</td><td>Fluid loading (500 ml saline solution NaCl 0,9%)</td><td>BP is 98/56 mmHg</td></tr>
</table>

Clinical case 12

You are taking care of a 38 year-old patient after a helmetless motobike accident. He has been intubated by the medical team in front of an alterated state of consioussness. He is hemodynamically unstable. BP is 96/55 mmHg with Norepinephrine 0,05 µg/kg/min. He already received a fluid loading (1500 mL). HR is 110/min.

He has a scalp wound (15 cm) with major bleeding.

<table>
<tr><th></th><th>You plan to :</th><th>But you learn :</th><th>Your original decision becomes :</th></tr>
<tr><td>TCS 27</td><td>Perform a body CT scan</td><td>He is hemodynamically stable under Norepinephrine</td><td rowspan="3">+2 almost certain
+1 more likely
0 unchanged
-1 less likely
-2 unlikely</td></tr>
<tr><td>TCS 28</td><td>Suture the scalp wound</td><td>There is an anisocoria</td></tr>
<tr><td>TCS 29</td><td>Hepatic artery embolization for hepatic rupture (grade IV)</td><td>He also have a pelvic fracture with constrast extravasation and the brain CT scan is normal</td></tr>
</table>
