# Supplementary material for: Use of the Smartphone App WhatsApp as an E-Learning Method for Medical Residents: Multicenter Controlled Randomized Trial
Source: JMIR Mhealth Uhealth. 2019 Apr 9;7(4):e12825. doi: 10.2196/12825 (PMC6477573; doi:10.2196/12825)
Supplement: Multimedia Appendix 5 [file mhealth_v7i4e12825_app5.pdf]

QCM Evaluation WhatsAR

1 : Chez le traumatisé crânien, quel est l'objectif de pression artérielle moyenne à la phase initiale ?

a. < 80 mmHg
b. Il n'existe pas d'objectif de PAM précis spécifique au traumatisme crânien
c. > 80 mmHg
d. > 120 mmHg
e. 60-70 mmHg

Réponse : C

2 : Vos objectifs transfusionnels en cas de choc hémorragique compliquant un polytraumatisme sans traumatisme crânien:

a. Hémoglobine = 6-7 g/dl
b. Hémoglobine = 7-9 g/dl
c. Fibrinogène > 3 g.L
d. Hémoglobine > 10 g/dl
e. Fibrinogène > 1.5g.L

Réponses : B, E

3. Quelle sont les 3 éléments de la triade létale chez un patient en état de choc hémorragique ?

a. Coagulopathie
b. Hypothermie
c. Acidose métabolique
d. Hyperthermie
e. Hyperkaliémie

Réponses : A,B,C

4. La transfusion de culots globulaires chez le patient traumatisé en état de choc hémorragique:

a. Doit être précédée des prélèvements des groupes, du phénotypage rhésus et de la recherche d'agglutinines irrégulières (RAI)
b. Peut se faire avec des culots globulaires de groupe O négatif
c. Impose systématiquement d'avoir les résultats des RAI avant le test de Beth-Vincent
d. Se fait dans le cadre d'une transfusion programmée
e. Doit être réalisée de façon conjointe avec une transfusion de PFC

Réponses : A,B,E

5. Lors d'un état de choc hémorragique :

a. Un traitement par Beta bloquant pré existant peut masquer la tachycardie reflexe
b. Une bradycardie peut survenir en cas d'hypovolémie extrême
c. Une vasodilatation périphérique compense initialement la chute du débit cardiaque
d. Une tachycardie réflexe est plus fréquemment présente chez la personne âgée que la personne jeune
e. Une tachycardie rélexe compense initialement la chute du débit cardiaque liée à l'hypovolémie

Réponses : A,B,E

6. L’acide tranexamique:

a. Réduit la transfusion et la mortalité du patient en choc hémorragique
b. A sa place dès la prise en charge pré hospitalière
c. Est un antidote de l’héparine
d. Est un mélange de facteurs de coagulation
e. Est un anti fibrinolytique

Réponses : A,B,E

7. Concernant les étiologies de choc hémorragique chez le polytraumatisé, quelle(s) est(sont) la(les) proposition(s) vraie(s) ?:

a. Un traumatisme médullaire peut être responsable d'une hémorragie massive
b. La fracture de rate est indétectable au scanner
c. La fracture transversale diaphysaire de jambe est une étiologie possible
d. Une fracture du bassin peut être responsable d'une hémorragie massive
e. La rupture de l'isthme aortique concerne l'aorte abdominale

Réponse : D

8. Les étiologies de saignement chez un traumatisé responsable d'un choc hémorragique peuvent être :

a. Rupture isthmique de l'aorte
b. Fracture de la diaphyse fémorale
c. Fracture de rate
d. Fracture de la diaphyse humérale
e. Hématome sous dural aigu

Réponses : A,B,C

9. Un jeune homme de 22 ans sans antécédents présente un accident de moto casqué, à 50 km/h. Il n'a pas perdu connaissance, se plaint de douleurs abdominales, et présente une déformation du fémur gauche sans ouverture cutanée. Pas de détresse respiratoire, Glasgow coma score 14/15. L'abdomen est sensible avec une défense de l'hypochondre gauche. Sa fréquence cardiaque est de 145/min, sa pression artérielle de 90-65 mmHg, l'hémocue initial de 13.2g/dl

Quelles thérapeutiques débutez-vous en pré hospitalier?

a. Remplissage vasculaire
b. Attelle de Donway
c. Favoriser l'hypothermie a visée neuroprotectrice
d. Commande de 2 culots globulaires pour transfusion préhospitalière
e. Acide tranexamique

Réponses : A,B,E

10. Chez un patient traumatisé, le bilan lésionnel scannographique retrouve un saignement actif sur la rate, un hémopéritoine important, une fracture de la diaphyse fémorale gauche. Au retour du scanner, la fréquence cardiaque passe à 140/min avec une hypotension artérielle à 80/50 mmHg. Le patient va au bloc opératoire pour une splénectomie d'hémostase en urgence.

Pour la gestion de l'hémodynamique, devant cet état de choc hémorragique, vous faites :

a. Une transfusion précoce de culots globulaire
b. Un remplissage massif par cristalloïdes pour un objectif de PA systolique > 100 mmHg
c. Une transfusion conjointe de 1 PFC pour 1 à 2 culots globulaires transfusés
d. La mise sous NORADRENALINE en cas d'hypotension réfractaire au remplissage
e. Une transfusion de plaquettes avant réception du bilan d'hémostase

Réponses : A,C,D

11. Vous accueillez un patient de 24 ans, AVP voiture ceinturé avec choc frontal dans un arbre à environ 80 km/h. A l'arrivée, FC à 112/min, PA 104/58 mmHg, GCS 14/15, SpO2 99% sous O2 6L/min au MHC. Le body-TDM retrouve l'image suivante. Quelles sont les bonnes réponses ?

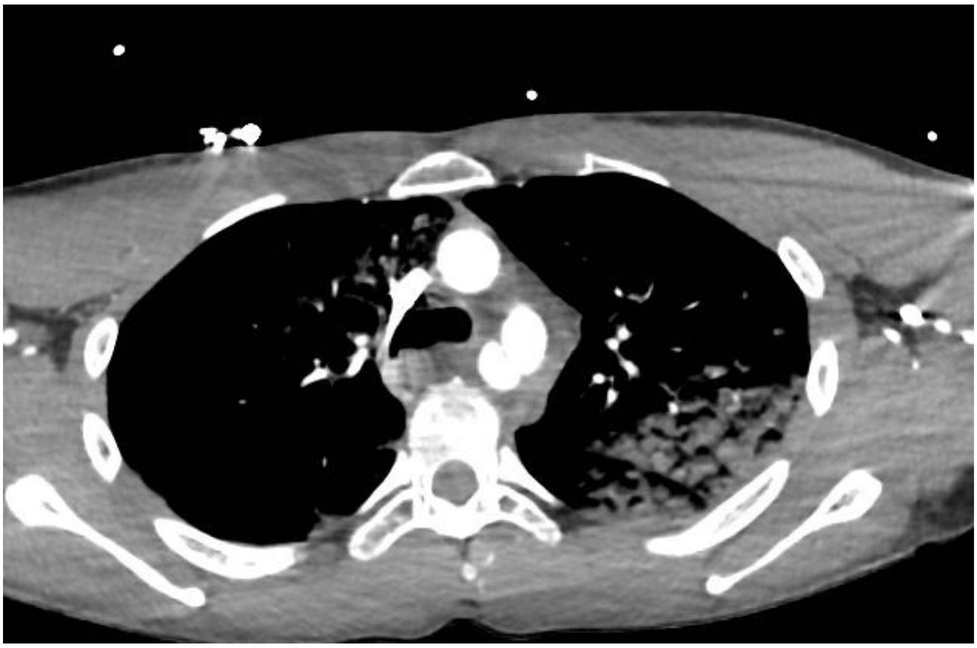

a. Il présente une dissection aortique de type B
b. Il présente une rupture de l'isthme aortique
c. Introduction de Noradrénaline pour objectif de PAM > 80 mmHg

d. Surveillance en milieu scopé, prise en charge chirurgicale si aggravation hémodynamique
e. Il est parfois possible de traiter cette lésion par endoprothèse

Réponses : B,E

12. Vous accueillez un patient de 74 ans, hémorragie active sur plaie du scalp et traumatisme complexe de la face après chute dans les escaliers (FC 120/min, PA 102/48 mmHg). Il est traité par AVK, vous n'avez pas encore son INR.
Quelle est votre stratégie d'antagonisation des AVK en urgence ?

a. Concentrés de complexe prothrombinique (CCP) 25 UI/kg + Vitamine K 10 mg IV
b. Attente de l'INR pour adapter la dose de concentrés de facteur à l'INR + Vitamine K 10 mg IV
c. Concentrés de complexe prothrombinique activés (FEIBA) 25 UI/kg + Vitamine K 10 mg IV
d. Concentrés de complexe prothrombinique (CCP) 50 UI/kg + Vitamine K 10 mg IV
e. Concentrés de complexe prothrombinique activés (FEIBA) 50 UI/kg + Vitamine K 10 mg IV

Réponse : A

13. Vous accueillez une patiente de 69 ans, choc hémorragique sur fracture de rate grade 4 après chute de vélo (FC 143/min, PA 91/43 mmHg). Elle est traitée par Apixaban pour une FA ancienne.
Quelle sont les stratégies possibles pour l'antagonisation de l'Apixaban?

a. Concentrés de complexe prothrombinique (CCP) 25 UI/kg
b. Perfusion d'Idarucizumab 5g IVL
c. Concentrés de complexe prothrombinique activés (FEIBA) 15-25 UI/kg
d. Concentrés de complexe prothrombinique (CCP) 50 UI/kg
e. Concentrés de complexe prothrombinique activés (FEIBA) 30-50 UI/kg

Réponse : D,E

14. Un homme de 54 ans, traité par AVK (Previscan ®) pour une phlébite récente est admis après un AVP VL / 2 roues. La TDM retrouve un hématome sous-capsulaire hépatique et une fracture du rein droit grade 3.
Vous antagonisez les AVK, quel est votre INR cible après antagonisation ?

a. INR < 1
b. INR < 1,2
c. INR < 1,5
d. INR < 1,8
e. INR < 2

Réponse : C

15. Vous accueillez au déchocage un homme de 22 ans, AVP moto non casqué avec traumatisme crânien (GCS = 6 à l'arrivée). Le body-TDM retrouve un hématome sous-dural droit et une fracture de rate grade 4. Quel est votre objectif de taux de plaquettes ?

a. > 20 000 / mm^3
b. > 50 000 / mm^3
c. > 80 000 / mm^3
d. > 100 000 / mm^3
e. > 150 000 / mm^3

Réponse : D

16. Quels sont les éléments impliqués dans l'hémostase primaire ?

a. Les plaquettes
b. Le facteur X
c. Le fibrinogène
d. La plasmine
e. Le calcium

Réponses : A, C, E

17. Quelles sont les indications à un drainage pyélique par sonde JJ en cas de trauma hémorragique du rein ?

a. Défaillance hémodynamique nécessitant l'introduction de Noradrénaline
b. Caillotage des voies urinaires
c. Fuite de produit de contraste au niveau du rein sans opacification urétérale
d. Fracture de rein grade 4 ou 5
e. Atteinte rénale bilatérale

Réponses : B,C

18. Vous accueillez au déchocage une femme de 32 ans, AVP scooter casqué avec traumatisme crânien (GCS = 6 à l'arrivée) et fracture des 2 fémurs. Sa PA est à 91/55 mmHg, sa FC à 133/min. Elle n'a pas encore reçu d'expansion volémique.
Quel soluté de remplissage choisissez-vous ?

a. Soluté glucosé 5%
b. NaCl 0,9%
c. Ringer Lactate
d. Hydroxy-Ethyl Amidon (Voluven ®)
e. Albumine 4%

Réponse : B

19. Parmi les atteintes traumatiques hémorragiques suivantes, lesquelles relèvent en 1ere intention d'une artério-embolisation chez le patient stabilisé sur le plan hémodynamique ?

a. Fracture de rate
b. Fracture hépatique
c. Fracture de fémur
d. Fracture de bassin
e. Plaie artérielle périphérique

Réponses : A,B,D

20. A propos de ce test ultime de concordance ultime ABO de Beth Vincent au lit du malade, quelle(s) est(sont) la(les) proposition(s) vraie(s) ?:

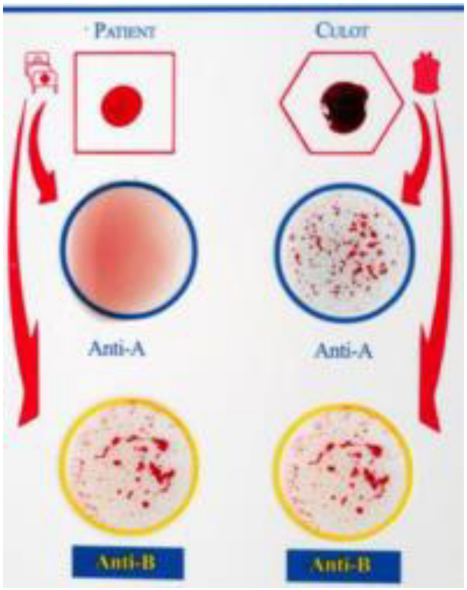

a. Ce test évalue la présence d'agglutinines irrégulières (AI)
b. Le culot globulaire peut être transfusé
c. Le culot globulaire est de groupe AB
d. Le culot globulaire est de groupe O
e. Le patient est du groupe A

Réponses : C

21. Quelle(s) est(sont) la(les) proposition(s) vraie(s) ?:

a. Lors d'une brèche vasculaire, il y a une vasodilatation réflexe
b. Lors d'une brèche vasculaire, l'adhésion, l'activation, puis l'agrégation des plaquettes sont trois étapes de l'hémostase primaire
c. L'hémostase primaire permet la transformation du fibrinogène en fibrine
d. L'hémostase primaire permet la formation d'un clou plaquettaire
e. Le fibrinogène est un des acteurs de l'hémostase primaire

Réponses : B,D,E

22. Quelle(s) est(sont) la(les) proposition(s) vraie(s) ?:

a. La génération de thrombine est explosive par auto-amplification après l'initiation de la coagulation
b. La génération de fibrine est ralentie par auto-inhibition après l'initiation de la coagulation
c. La coagulation permet la transformation d'un clou plaquettaire en un réseau de fibrine soluble
d. Le calcium est un cofacteur nécessaire à la coagulation
e. Les facteurs VIII, IX, XI, XII, font partie de la voie intrinsèque

Réponses : A,D,E

23. Quelles sont les réponses vraies ?

a. L’antithrombine est le principal activateur de la coagulation
b. La thrombomoduline se lie à la thrombine et entraine une activation de la protéine C
c. La plasmine dégrade la fibrine
d. La plasmine dégrade le fibrinogène
e. Le plasminogène dégrade la fibrine

Réponse : B,C,D

24. Parmi les facteurs suivant, lesquels sont vitamine K dépendants ?

a. II
b. V
c. VII
d. VIII
e. X

Réponses : A,C,E

25. Quelles sont les deux hypothèses de la coagulopathie du polytraumatisé ?

a. Coagulopathie de déplétion
b. Coagulopathie de consommation
c. Inhibition de la protéine C
d. Activation de la protéine C
e. Séquestration de la protéine C

Réponses : B,D

26. Lors d’une transfusion massive en urgence vitale immédiate chez un homme, vous demandez :

a. Culot globulaire iso groupe iso rhésus
b. Culot globulaire RH : 1 KEL : – 1
c. Culot globulaire RH : 1 KEL : 1
d. PFC iso groupe
e. PFC AB

Réponses : B,E

27. L’objectif de taux d’hémoglobine chez un patient coronarien en choc hémorragique est :

a. Entre 6 - 7 g/ dL
b. Entre 7 - 8 g/dL
c. Entre 8 - 9 g/dL
d. Entre 9 - 10 g/dL

e. Pas de recommandation précise chez cette population

Réponse : D

28. Faut – il monitorer la concentration de calcium ionisé chez les patients en choc hémorragiques (plusieurs réponses possibles) ?

a. Oui, car le calcium permet d'optimiser l'hémostase.
b. Oui, car une hypocalcémie peut survenir lors d'une transfusion massive.
c. Non, car le métabolisme du citrate est préservé en cas de défaillance hémodynamique.
d. Oui, car les dyscalcémies peuvent entrainer des troubles de conduction cardiaque.
e. Oui, car la transfusion massive peut entrainer une hypercalcémie par un apport excessif de calcium dans les produits sanguin labiles.

Réponses : A, B, D

29. L'acide tranexamique:

a. Est un médicament anti-fibrinolytique.
b. Est un produit dérivé du sang.
c. Doit être initié à la posologie de 3 g sur 10 minutes en IVL.
d. Est contre-indiqué chez la femme enceinte.
e. Est contre-indiqué chez le coronarien.

Réponses : A

30. Le concentré de complexes prothrombiniques activés (FEIBA®) :

a. Est un médicament dérivé du sang.
b. Contient les facteurs II, VII, IX, X.
c. Contient les facteurs IIa, VIIa, IXa, Xa.
d. Sont destinés à l'antagonisation des AVK en première intention.
e. Sont destinés à l'antagonisation des NACO en première intention.

Réponses : A,C,E

MCQ WhatsAR Evaluation

1 : What is the mean arterial pressure (MAP) target after a traumatic brain injury (TBI) at the initial stage?

a. < 80 mmHg
b. There is no specific MAP target after TBI
c. > 80 mmHg
d. > 120 mmHg
e. 60-70 mmHg

Answer: C

2. Your transfusion thresholds in case of hemorrhagic shock in trauma patient without TBI:

a. Hemoglobin = 6-7 g/dl
b. Hemoglobin = 7-9 g/dl
c. Fibrinogen > 3 g.L
d. Hemoglobin > 10 g/dl
e. Fibrinogen > 1.5g.L

Answers: B, E

3. What are the 3 elements of the hemorrhagic shock's lethal triad?

a. Coagulopathy
b. Hypothermia
c. Metabolic acidosis
d. Hyperthermia
e. Hyperkalemia

Answers: A,B,C

4. Red blood cells transfusion in case of hemorrhagic shock in polytrauma patients:

a. Must be preceded by test for ABO group, Rhesus phenotype and irregular agglutinins research.
b. Can be done with group O RhD negative red blood cells
c. Systematically requires the result of the irregular agglutinins research before performing the Beth-Vincent test
d. Is done as part of a scheduled transfusion
e. Must be performed associated with FFP

Answers: A,B,E

5. In a state of hemorrhagic shock:

a. Beta blockers may mask reflex tachycardia
b. Bradycardia may occur in cases of severe hypovolemia
c. Peripheral vasodilation initially occurs in order to maintain cardiac output
d. Reflex tachycardia is more common in the elderly than in young people

e. Reflex tachycardia initially compensates for the drop of cardiac output associated with hypovolemia
Answers: A,B,E

6. Tranexamic acid:
a. Reduces transfusion and patient mortality in hemorrhagic shock
b. Is used as soon as primary care is provided
c. Is an antidote to heparin
d. Is a mixture of coagulation factors
e. Is an antifibrinolytic drug
Answers: A,B,E

7. Which of the following statements about hemorrhagic shock etiologies in trauma patients is/are True?
a. Spinal cord injury can cause massive bleeding
b. The spleen fracture is undetectable on the scanner
c. Diaphyseal transverse leg fracture is a possible etiology
d. A fractured pelvis can cause massive bleeding
e. The rupture of the aortic isthmus concerns the abdominal aorta
Answer: D

8. Sources of bleeding in blunt trauma patient with hemorrhagic shock can be:
a. Isthmic rupture of the aorta
b. Diaphyseal femur fracture
c. Spleen fracture
d. Diaphyseal humerus fracture
e. Acute subdural hematoma
Answers: A,B,C

9. A 22-year-old man with no previous record has a helmeted motorcycle crash at 50 km/h. He did not lose consciousness, complains of abdominal pain, and has a deformity of the left femur with intact overlying skin. No respiratory distress, Glasgow coma scale (GCS) 14/15. He has abdominal pain and defense of the left hypochondrium. His heart rate is 145 bpm, his blood pressure is 90-65 mmHg, his initial Hemocue is 13.2g/dl
Which therapeutics do you start in primary care?
a. Vascular filling
b. Donway Splint
c. Therapeutic hypothermia for neuroprotective purposes
d. Prehospital transfusion of 2 units of packed red blood cells
e. Tranexamic acid
Answers: A,B,E

10. Assessment by CT scan in a blunt trauma patient finds active splenic bleeding, significant hemoperitoneum, a fracture of the left femoral shaft. On the way back from the CT scan, the heart rate increases to 140bpm with a low blood pressure of 80/50 mmHg. The patient is reffered urgently to the operating room for an emergency hemostasic splenectomy.
For hemodynamic management of this hemorrhagic shock, you do:

a. Early transfusion of red blood cells
b. A massive crystalloid filling for a systolic BP lens > 100 mmHg
c. A joint transfusion of 1 FFP for 1 to 2 units of packed red blood cells
d. The use of norepinephrine in case of hypotension refractory to vascular filling
e. A platelet transfusion before receiving the hemostasis test

Answers: A,C,D

11. A 24-year-old patient just had a motor vehicle accident with frontal crash into a tree at 80 km/h. On arrival, HR at 112bpm, BP 104/58 mmHg, GCS 14/15, SO2 99% with high concentration oxygen mask 6L/min. CT finds the following image. Wich of the following statements is/are true ?

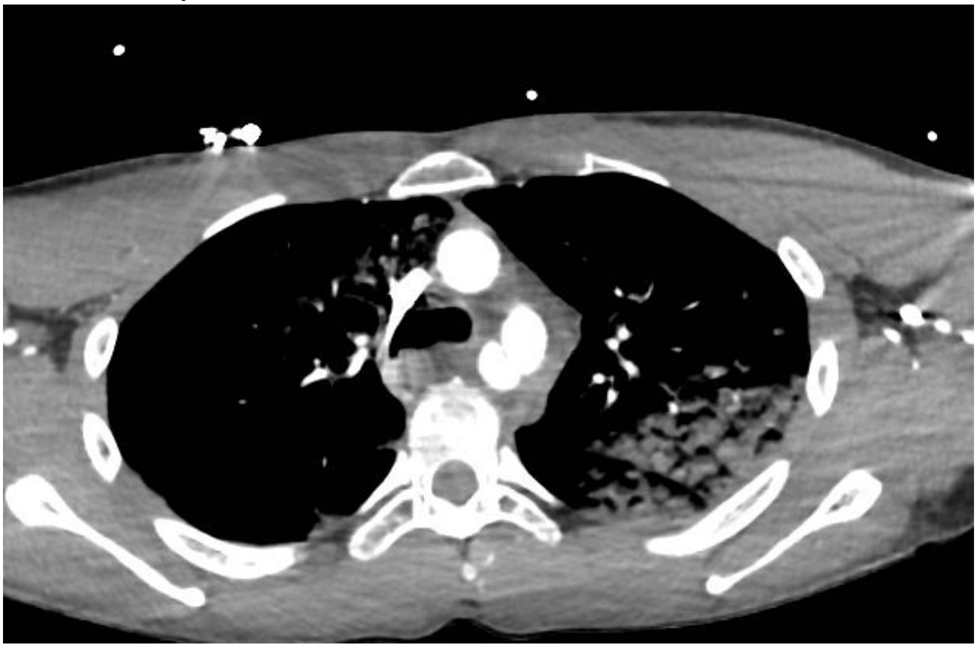

a. This is a type B aortic dissection
b. This is a traumatic rupture of the aortic isthmus
c. Introduction of norepinephrine for MAP target > 80 mmHg
d. Continuous monitoring, surgical management if hemodynamic worsening
e. This lesion can sometimes be treated with vascular stenting

Answers: B,E

12. You have a 74-year-old patient with active scalp wound hemorrhage and complex facial trauma after falling down the stairs (HR 120bpm, BP 102/48 mmHg). He's being treated by VKA, you don't have his INR yet.
What is your strategy to antagonize VKA urgently?

a. Prothrombin complex concentrates (PCC) 25 IU/kg + Vitamin K 10 mg IV

b. Waiting for the INR to adapt the dose of clotting factor concentrates to the INR + Vitamin K 10 mg IV
c. Activated prothrombin complex concentrates (aPCC) 25 IU/kg + Vitamin K 10 mg IV
d. Prothrombin complex concentrates (PCC) 50 IU/kg + Vitamin K 10 mg IV
e. Activated prothrombin complex concentrates (aPCC) 50 IU/kg + Vitamin K 10 mg IV
Answer: A

13. You have a 69-year-old patient with a grade 4 ruptured spleen hemorrhagic shock after a bicycle fall (HR 143bpm, BP 91/43 mmHg). She takes Apixaban for atrial fibrillation. What are the possible strategies for the antagonization of Apixaban?
a. Prothrombin complex concentrates (PCC) 25 IU/kg
b. Idarucizumab 5g IV slow infusion
c. Activated prothrombin complex concentrates (aPCC) 15-25 IU/kg
d. Prothrombin complex concentrates (PCC) 50 IU/kg
e. Activated prothrombin complex concentrates (aPCC) 30-50 IU/kg
Answer: D,E

14. A 54-year-old male, treated with VKA (Previscan ®) for recent phlebitis is admitted after an motor vehicle accident (car vs motorcycle). CT finds a subcapsular hematoma of the liver and a grade 3 right renal laceration.
You antagonize VKA. What is your target INR after antagonization?
a. INR < 1
b. INR < 1.2
c. INR < 1.5
d. INR < 1.8
e. INR < 2
Answer: C

15. You have a 22-year-old man, motorcycle crash without helmet with traumatic brain injury (GCS = 6 on arrival). What is your target for platelet count?
a. > 20,000 / mm3
b. > 50,000 / mm3
c. > 80,000 / mm3
d. > 100,000 / mm3
e. > 150,000 / mm3
Answer: D

16. What are the elements involved in primary hemostasis?
a. Platelets
b. Factor X
c. Fibrinogen
d. Plasmin
e. Calcium

Answers: A, C, E

17. What are the indications for ureteral probe in case of traumatic renal bleeding?

a. Hemodynamic failure requiring norepinephrine
b. Obstructing blood clot in the ureter
c. Renal contrast extravasation without ureteral opacification
d. Grade 4 or 5 kidney rupture
e. Bilateral renal impairment

Answers: B,C

18. You have a 32-year-old woman, helmeted motorcycle crash traumatic brain injury (GCS = 6 on arrival) and fracture of both femur. HR 133bpm, BP 91/55 mmHg. She has not received yet any IV volume expansion. Which vascular filling solution do you choose?

a. 5% Glucose solution
b. 0,9% saline (NaCl)
c. Ringer Lactate
d. Hydroxy-Ethyl Starch (Voluven ®)
e. Albumin 4%

Answer: B

19. Which of the following traumatic hemorrhagic disorders are first intentionally treated by arterioembolization in the hemodynamically stabilized patient?

a. Splenic fracture
b. Liver fracture
c. Femoral fracture
d. Pelvic fracture
e. Peripheral arterial bleeding

Answers: A,B,D

20. About this ABO blood group test (Beth Vincent), which of the following statements is/are true?

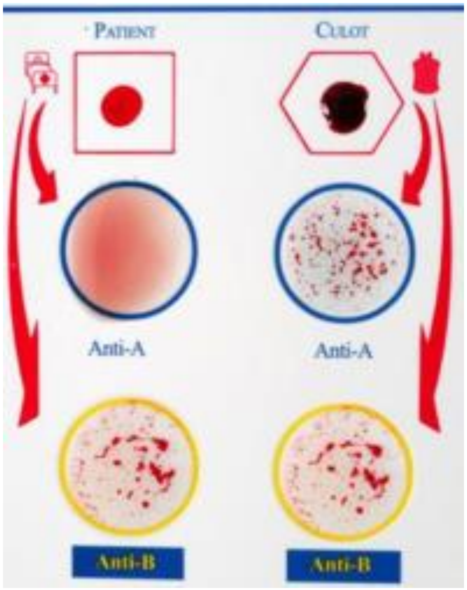

a. This test assesses the presence of irregular agglutinins
b. Red blood cells can be transfused
c. Blood type group of the RBCC is AB
d. Blood type group of the RBCC is is O
e. Blood type group of the RBCC is is A
Answers: C

21. Which of the following statements is/are true?
a. In vascular injury, there is a reflex vasodilation
b. In vascular injury, platelets adhesion, activation and aggregation are the 3 steps of primary hemostasis
c. Primary hemostasis allows the transformation of fibrinogen into fibrin
d. Primary hemostasis is the formation of the primary platelet plug.
e. Fibrinogen is one of many actors in primary hemostasis
Answers: B,D,E

22. Which of the following statements is/are true?
a. Thrombin generation is explosive by self-amplification after coagulation initiation
b. Fibrin generation is slowed by self-inhibition after coagulation initiation
c. Coagulation allows the transformation of a platelet plug into a network of soluble fibrin
d. Calcium is a cofactor required for coagulation
e. Factors VIII, IX, XI, XI and XII are part of the intrinsic pathway
Answers: A,D,E

23. Which of the following statements is/are true?
a. Antithrombin is the main coagulation activator
b. Thrombomodulin binds to thrombin and causes protein C activation
c. Plasmine degrades fibrin
d. Plasmine degrades fibrinogen

e. Plasminogen degrades fibrin

Answer: B,C,D

24. Which of the following factors are vitamin K dependent?

a. II

b. V

c. VII

d. VIII

e. X

Answers: A,C,E

25. What are the two hypotheses of trauma-induced coagulopathy?

a. Depletion of coagulation factors

b. Consumption coagulopathy

c. Inhibition of protein C

d. Activation of protein C

e. Sequestration-induced loss of protein C receptors

Answers: B,D

26. In case of an immediate life-threatening situation with massive transfusion urgently needed, you ask:

a. Red blood cell iso group / iso rhesus

b. Red blood cell RH : 1 KEL : - 1

c. Red blood cell RH : 1 KEL : 1

d. PFC iso group

e. PFC AB group

Answers: B,E

27. Hemoglobin level in hemorrhagic shock for patients at risk for myocardial infarction is:

a. Between 6 - 7 g/ dL

b. Between 7 - 8 g/dL

c. Between 8 - 9 g/dL

d. Between 9 - 10 g/dL

e. No specific recommendation for this population

Answer: D

28. Should plasma ionized calcium concentration be monitored in hemorrhagic shock (several possible responses)?

a. Yes, because calcium is indispensable for optimizing patient's coagulation

b. Yes, because hypocalcemia may occur during a massive transfusion.

c. No, because the citrate metabolism is preserved in case of hemodynamic failure.

d. Yes, because dyscalcemia can lead to cardiac conduction disorders.

e. Yes, because massive transfusion can lead to hypercalcemia by excessive calcium intake through labile blood products.

Answers: A, B, D

29. Tranexamic acid:

a. Is an anti-fibrinolytic drug.
b. Is a product blood-derived product.
c. Must be initiated at a dose of 3g IV slow infusion (10 minutes).
d. Is contraindicated in pregnant women.
e. Is contraindicated in coronary artery disease.

Answers: A

30. Activated prothrombin complex concentrate (aPCC):

a. Is a blood-derived drug.
b. Contains factors II, VII, IX, X.
c. Contains factors IIa, VIIa, IXa, IXa, Xa.
d. Are intended for antagonization of first-line VKA.
e. Are intended for first-line antagonization of new oral anticoagulants.

Answers: A,C,E
