# Supplementary material for: Use of the Smartphone App WhatsApp as an E-Learning Method for Medical Residents: Multicenter Controlled Randomized Trial
Source: JMIR Mhealth Uhealth. 2019 Apr 9;7(4):e12825. doi: 10.2196/12825 (PMC6477573; doi:10.2196/12825)
Supplement: Multimedia Appendix 6 [file mhealth_v7i4e12825_app6.pdf]

Grille d'évaluation du contenu pédagogique et de satisfaction de l'enseignement

Partie 1 : *Chaque item noté de 0 à 10*

1. Quelle note globale de satisfaction donneriez-vous à l'enseignement que vous avez reçu ? (0 à 10)

2. Comment estimez-vous la qualité des documents pédagogiques proposés ? (0 très mauvais à 10 Très bons)

3. Estimez-vous que l'enseignement reçu répond correctement à l'objectif de former des DESAR sur la prise en charge du choc hémorragique du polytraumatisé ? (0 Pas du tout à 10 parfaitement)

4. Avez-vous trouvé la présence de cas cliniques utile ? (0 Totalement inutile à 10 Très utile)

5. Avez-vous trouvé que les organisateurs étaient disponibles ? (0 Totalement indisponibles à 10 Très disponibles)

Partie 2 : *4-5 propositions possibles pour chaque item*

6. Combien de temps avez-vous passé à lire les documents et à participer aux enseignements ? (aucune participation/lecture, entre 1 et 5h, entre 5 et 10h, plus de 10h)

7. Quel proportion de documents avez-vous consulté (Aucun, moins de 50%, plus de 50%, la totalité) ?

8. Avez-vous trouvé que le niveau des documents était : (beaucoup trop facile, facile, adapté, difficile, trop difficile, je n'ai pas consulté les documents)

9. Avez-vous trouvé les QCM de l'évaluation sur le choc hémorragique : (beaucoup trop faciles, faciles, adaptés, difficiles, trop difficiles)

10. Avez-vous trouvé les TCS de l'évaluation sur le choc hémorragique : (beaucoup trop faciles, faciles, adaptés, difficiles, trop difficiles)

Partie 3 : *spécifique pour les étudiants du groupe WhatsApp*

a. Trouvez-vous que l'utilisation de WhatsApp pour l'enseignement médical soit pertinent ? (OUI/NON)

b. Seriez-vous prêt à participer à nouveau à un enseignement médical via la plateforme WhatsApp ? (OUI/NON)

c. Avez-vous trouvé que le fait d'avoir régulièrement des notifications WhatsApp de ce projet était gênant ? (OUI/NON)

Partie 4 : *Commentaire libre et suggestions*

Survey satisfaction

Part 1: Each item rated from 0 to 10

1. What is your overall satisfaction rating for the teaching you received? (0 à 10)
2. How do you rate the quality of the proposed educational documents? (0 = very bad to 10 = Very good)
3. Do you consider that the teaching received is in line with the objective of training on the management of hemorrhagic shock in trauma? (0 Not at all to 10 perfectly)
4. Did you find the presence of clinical cases useful? (0 = Totally useless to 10 = Very useful)
5. Did you find that the organizers were available? (0 = Totally unavailable at 10 = Very available)

Part 2 :

6. How much time did you spend reading the documents and participating in the teachings? (no participation/reading, between 1 and 5 hours , between 5 and 10 hours, more than 10 hours)
7. What proportion of documents did you consult (None, less than 50%, more than 50%, all)?
8. Did you find the level of the documents to be: (too easy, easy, adapted, difficult, very difficult, too difficult, I did not consult the documents)
9. What is your opinion about the MCQ for the hemorrhagic shock assessment? (too easy, easy, adapted, difficult, very difficult, too difficult)
10. What is your opinion about the SCT for the hemorrhagic shock assessment? (too easy, easy, adapted, difficult, very difficult, too difficult)

Part 3: Specific to WhatsApp group

a. Do you find the use of WhatsApp for medical education relevant? (YES/NO)

b. Would you be willing to participate again in a pedagogic program via the WhatsApp platform? (YES/NO)

c. Did you find it embarrassing to have regular WhatsApp notifications of this project? (YES/NO)

Part 4: Free Comments and Suggestions
